# Fragility fractures in Europe: burden, management and opportunities

**DOI:** 10.1007/s11657-020-0706-y

**Published:** 2020-04-19

**Authors:** Fredrik Borgström, Linda Karlsson, Gustav Ortsäter, Nicolas Norton, Philippe Halbout, Cyrus Cooper, Mattias Lorentzon, Eugene V. McCloskey, Nicholas C. Harvey, Muhamamd K. Javaid, John A. Kanis, for the International Osteoporosis Foundation, Cyrus Cooper, Jean-Yves Reginster, Serge Ferrari, Philippe Halbout

**Affiliations:** 1 Medical Management Centre, Department of Learning Informatics, Management and Ethics, Karolinska Institute, Solna, Sweden; 2 Quantify Research, Stockholm, Sweden; 3 International Osteoporosis Foundation, Nyon, Switzerland; 4 MRC Lifecourse Epidemiology Unit, University of Southampton, Southampton, UK; 5 National Institute for Health Research (NIHR) Musculoskeletal Biomedical Research Unit, University of Oxford, Oxford, UK; 6 Mary MacKillop Health Institute, Catholic University of Australia, Melbourne, Australia; 7 Geriatric Medicine, Department of Internal Medicine and Clinical Nutrition, Institute of Medicine and Clinical Nutrition, Sahlgrenska Academy, University of Gothenburg, Gothenburg, Sweden; 8 Centre for Metabolic Bone Diseases, University of Sheffield Medical School, Beech Hill Road, Sheffield, S10 2RX UK; 9 MRC and Arthritis Research UK Centre for Integrated Research in Musculoskeletal Ageing, Mellanby Centre for Bone Research, University of Sheffield, Sheffield, UK

**Keywords:** Disability-adjusted life years, Fragility fracture, Fracture costs, Treatment gap, Quality-adjusted life years

## Abstract

**Summary:**

This report provides an overview and a comparison of the burden and management of fragility fractures in the largest five countries of the European Union plus Sweden (EU6). In 2017, new fragility fractures in the EU6 are estimated at 2.7 million with an associated annual cost of €37.5 billion and a loss of 1.0 million quality-adjusted life years.

**Introduction:**

Osteoporosis is characterized by reduced bone mass and strength, which increases the risk of fragility fractures, which in turn, represent the main consequence of the disease. This report provides an overview and a comparison of the burden and management of fragility fractures in the largest five EU countries and Sweden (designated the EU6).

**Methods:**

A series of metrics describing the burden and management of fragility fractures were defined by a scientific steering committee. A working group performed the data collection and analysis. Data were collected from current literature, available retrospective data and public sources. Different methods were applied (e.g. standard statistics and health economic modelling), where appropriate, to perform the analysis for each metric.

**Results:**

Total fragility fractures in the EU6 are estimated to increase from 2.7 million in 2017 to 3.3 million in 2030; a 23% increase. The resulting annual fracture-related costs (€37.5 billion in 2017) are expected to increase by 27%. An estimated 1.0 million quality-adjusted life years (QALYs) were lost in 2017 due to fragility fractures. The current disability-adjusted life years (DALYs) per 1000 individuals age 50 years or more were estimated at 21 years, which is higher than the estimates for stroke or chronic obstructive pulmonary disease. The treatment gap (percentage of eligible individuals not receiving treatment with osteoporosis drugs) in the EU6 is estimated to be 73% for women and 63% for men; an increase of 17% since 2010. If all patients who fracture in the EU6 were enrolled into fracture liaison services, at least 19,000 fractures every year might be avoided.

**Conclusions:**

Fracture-related burden is expected to increase over the coming decades. Given the substantial treatment gap and proven cost-effectiveness of fracture prevention schemes such as fracture liaison services, urgent action is needed to ensure that all individuals at high risk of fragility fracture are appropriately assessed and treated.

**Electronic supplementary material:**

The online version of this article (10.1007/s11657-020-0706-y) contains supplementary material, which is available to authorized users.

## Introduction

The objective of this report is to provide information on the current and future burden of osteoporosis and associated fragility fractures as well as to describe current management of the disease. Results are presented for the five largest EU countries (France, Germany, Italy, Spain and the UK) as well as Sweden, referred to as the EU6. This report was developed by the International Osteoporosis Foundation (IOF) and led by a steering committee of scientific experts assigned by the IOF. The report forms the basis of policy reports prepared by IOF for each of the EU6 countries [1–7] .

To facilitate an assessment and a comparison of the burden and management of fragility fractures, a series of metrics was defined by a steering committee and thereafter quantified by a group of analysts at Quantify Research (reflected in the authorship). The metrics were classified into two broad categories with subcategories. The first category was burden of disease with epidemiology, economic cost and patient burden as subcategories. The second category was management of disease with service provision and service uptake as subcategories. The first part of this report provides a summary of the most important findings. An appendix that follows provides more detailed information on each metric, particularly on the analytic methods.

### Osteoporosis

Osteoporosis, which means porous bone, is a disease that weakens the bones and increases the risk of fragility fractures, where bones can break from low level impact or stress that would not normally break a healthy bone. Since bones become more porous and fragile with age, the disease is mainly found in the older population, and is more common among women than men [8].

Bone mineral density (BMD) is the measurement used to determine whether an individual has osteoporosis. The operational definition of osteoporosis is based on the T-score for BMD in women [9, 10] and is defined as a value for BMD 2.5 SD or more below the young female adult mean (T-score less than or equal to − 2.5).

The clinical relevance of osteoporosis lies in the associated fragility fractures; until such an event occurs, there are usually no symptoms [8]. In the Western World, about 1 in 3 women and 1 in 5 men above 50 years of age will fracture during their remaining life time [11]. After the age of 50 years, most sites of fracture can be considered characteristic of osteoporosis. Fractures at the hip and vertebrae are among the most common and serious sites of osteoporotic fracture. Fragility fractures of the humerus, forearm, ribs, tibia (in women, but not including ankle fractures), pelvis and other femoral fractures after the age of 50 years are fractures associated with low BMD [12, 13].

Worldwide, osteoporosis causes more than 9 million fractures a year, meaning there is a fragility fracture every 3 s [14]. Those who have had their first osteoporotic fracture have a higher risk for further fractures. The risk of fracture also increases with age, and as average life expectancy around the world rises, more individuals are expected to sustain fragility fractures.

The fracture-related monetary cost of fragility fractures in the 27 countries of the EU (EU27) has been estimated at €37 billion in 2010 [15], with 26,300 life years lost and 1.16 million quality-adjusted life years (QALYs) lost on a yearly basis [15]. With changing demography, these costs are expected to increase considerably by the year 2030.

Despite significant impacts on health and quality of life for the older population, there is a general lack of awareness of osteoporosis, including many health care agencies, which results in suboptimal care. Indeed, most individuals at high risk are never identified nor given appropriate treatment, which gives rise to further fragility fractures and worsening of health status.

The primary outcomes of interest in this report were fractures considered to be related to low BMD [12]. These include clinical vertebral fractures, fractures of distal forearm, pelvis-sacrum, ribs-sternum, clavicle, humerus and proximal femur. Fractures of the hands, feet, ankle, skull and facial bones were excluded. The report also focuses on specific fracture sites: hip fracture, clinical vertebral fracture and major osteoporotic fracture (MOFs). MOF is a grouping of the most common fractures comprising hip, clinical vertebral, distal forearm and proximal humerus fractures. The term ‘other’ osteoporotic fractures in this report refers to osteoporotic fractures that are not MOFs unless specifically defined. The majority of vertebral fractures are subclinical (75%) and recognised on radiographs by a change in shape of the vertebral body [10]. In the present report, clinical vertebral fractures coming to medical attention are considered rather than these morphometric fractures.

## Epidemiology of fragility fractures

### Prevalence of osteoporosis

About one-tenth of women age 60 years, one-fifth of women age 70, two-fifths of women age 80 and two-thirds of women aged 90 years have osteoporosis and an increased risk of fragility fracture [16]. Worldwide, approximately 200 million women have osteoporosis [17] defined as a value for femoral neck BMD 2.5 SD or more below the young female adult mean (T-score less than or equal to − 2.5) [10]. Note that the BMD threshold applies to men as well as women.

**Fig. 1:**
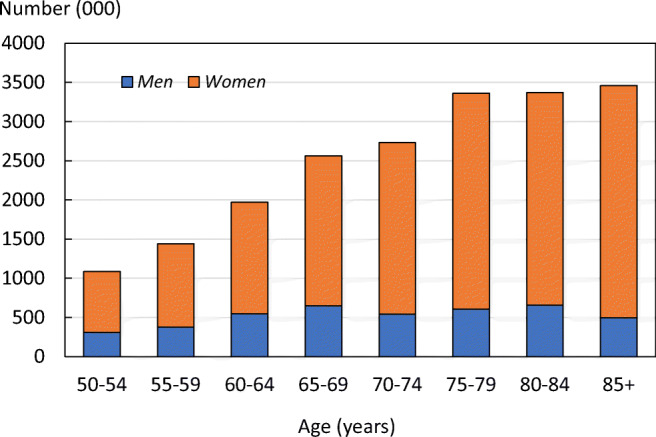
Prevalence of osteoporosis in the EU6 by age and sex

In 2015, there were an estimated 20 million individuals with osteoporosis in the EU6. Of those, 15.8 million were women and 4.2 million were men. The number of women with osteoporosis increased markedly with age (Fig. 1). The prevalence of osteoporosis at the age of 50 years or more, as judged by femoral neck BMD, was 6.8% in men and 22.5% in women.

Country-specific estimates for individuals with the disease age 50 years or older in women ranged from 21.8% (UK) to 23.1% (Italy). For men, the number with osteoporosis ranged from 6.7% (Germany) to 7.0% (Italy). For country-specific details and methods, see the Appendix (1: Prevalence of osteoporosis).

Osteoporosis represents one of the greatest health risks for individuals age 50 years or more, even when compared to hypercholesterolaemia and hypertension (two major contributors to heart disease), which affect 54% and 44% of people age 50 years or more, respectively [18].

### Number of fractures

There were estimated to be 2.7 million new fragility fractures in the EU6 in 2017—equivalent to 7332 fractures/day (or 305/h) (Table 1). Almost twice as many fractures occurred in women (66%) compared to men. Hip, vertebral and distal forearm/proximal humerus fractures accounted for 19.6, 15.5 and 17.9% of all fractures, respectively. Other fragility fractures accounted for 49% of the fracture burden.

**Table 1:** Estimated number of incident fragility fractures in the EU6 by site in 2017

Fracture site	Women	Men	Men and women
Hip	381,732	144,738	526,470
Spine	267,194	148,089	415,283
Proximal humerus/distal forearm	303,021	175,020	478,041
Other	819,029	437,397	1,256,426
All	1,770,976	905,244	2676,220

**Fig. 2:**
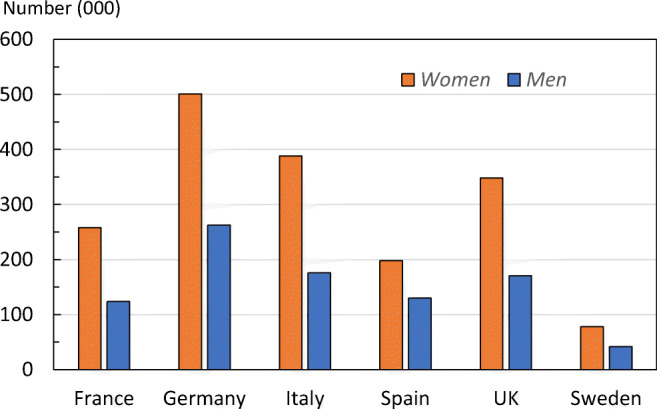
Number (thousands) of new fragility fractures by country in 2017

The number of new fragility fractures in 2017 by country is shown in Fig. 2. Germany had the highest number of fractures in both men and women—approximately 765,000 incident fractures in total, predominately reflecting the large population size and comparatively high fracture incidence.

When fracture numbers were expressed as a rate of the population at risk, there was a greater than two-fold range in risk that varied from 15/1000 in France to 32/1000 in Sweden (Table 2).

**Table 2:** The number of new fragility fractures in 2017 in men and women by country, the population at risk (men and women aged 50 years or more) and the crude incidence (/1000 of the population)

Country	New fractures (000)	Population at risk (000)	Rate/1000
France	381.6	24,672	15
Germany	764.9	33,399	23
Italy	563.4	26,282	21
Spain	327.6	16,510	20
UK	519.0	24,048	22
Sweden	119.7	3787	32
EU6	2676.2	128,699	21

A detailed breakdown of number of fractures by site and country is given together with the methods in the Appendix (2: Lifetime risk of fragility fractures).

### Lifetime risk of fragility fracture

**Fig. 3:**
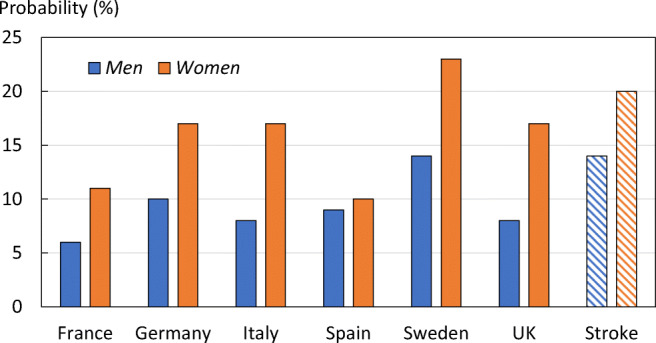
Lifetime risk of hip fracture from the age of 50 years, by country and sex, and the equivalent risk for stroke

The remaining lifetime risk of sustaining a hip fracture for women at the age of 50 years varied between 9.8% for Spain to 22.8% for Sweden (Fig. 3). The corresponding risk range for men was 6.1% (France) to 13.7% (Sweden). The lifetime risk of hip fracture at age 50 years was comparable to the lifetime risk of a stroke in Europe for both women (20%) and men (14%) [22].

**Fig. 4:**
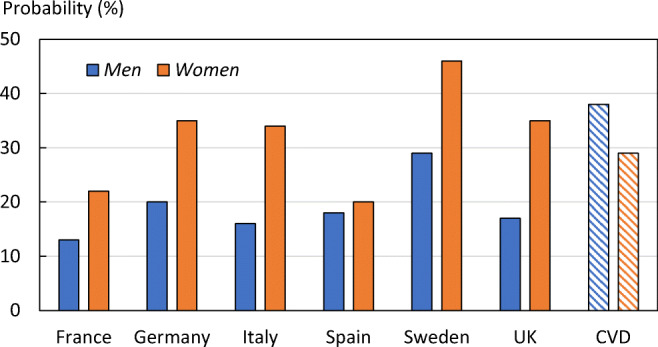
Lifetime risk of fragility fracture from the age of 50 years, by country and sex, and the equivalent risk for cardiovascular disease (CVD). Source: National fracture incidences and own calculations

The remaining lifetime probability of a MOF was highest in Sweden (46.3% for women and 28.7% for men (Fig. 4). Lifetime risk of major osteoporotic fracture was comparable to that of cardiovascular disease (CVD) in Europe, which affects 29% of women and 38% of men [19]. For methods and numerical data by fracture site and country, see the Appendix (2: Lifetime risk of fragility fractures).

### Fracture projections

There is a marked difference in the risk of fracture between countries [20]. Northern European countries have the highest fracture rates observed worldwide. The reasons for the difference in fracture risk are unknown but cannot be explained by differences in bone density. Plausible factors include differences in body mass index, low calcium intake, reduced sunlight exposure and perhaps the most crucial factor, high socio-economic status, which in turn may be related to low levels of physical activity [21, 22]. Regardless of differences in fracture risk, the number of fractures in all countries is expected to increase due to an increasingly ageing population.

**Fig. 5:**
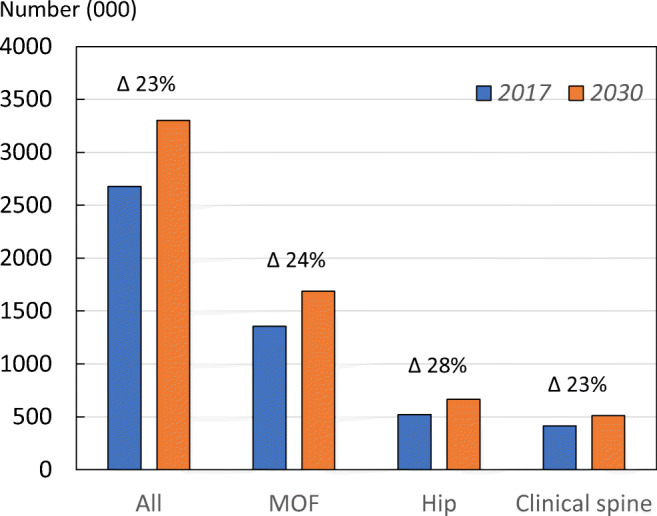
Estimated number of fragility fractures by fracture category in 2017 and 2030. Numbers denote the percentage change for all fragility fractures, major osteoporotic fractures (MOF), hip and clinical spine fractures

To estimate the annual number of new fractures between 2017 and 2030, national data on fracture incidence by type and sex were combined with demographic projections over time (see Appendix, 3: Fracture projections). The total number of all fragility fractures in the EU6 is projected to increase from 2.7 million in the year 2017 to 3.3 million in 2030; an increase of 23.3% (Fig. 5). In total, 66.2% of fragility fractures were sustained by women in 2017. The total number of MOF was 1.4 million and expected to increase by 24%. For hip fracture (*n* = 526 thousand) and clinical spine fracture (*n* = 416 thousand), the increases projected were 28% and 23%, respectively.

**Fig. 6:**
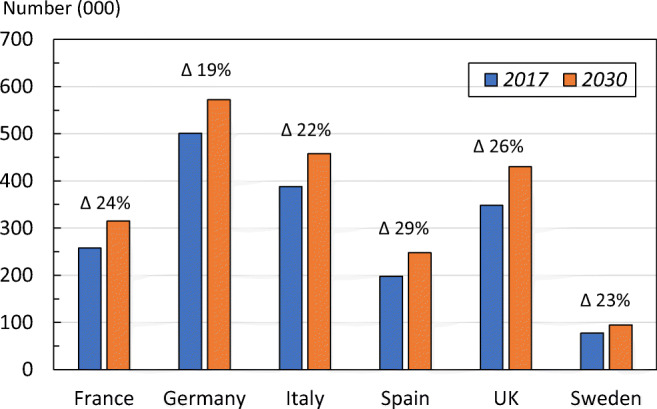
Number of fragility fractures by country in the EU6 and the projected numbers in 2030

Variations in projections were seen between countries (Fig. 6). For example, the highest percentage increase in all osteoporotic fractures was noted in Spain (28.8%) and the lowest in Germany (18.5%), due to differences in projected populations over time up to 2030. Country-specific details for hip, vertebral fractures and MOFs are given in the Appendix (3: Fracture projections).

### Imminent risk of fracture

**Fig. 7:**
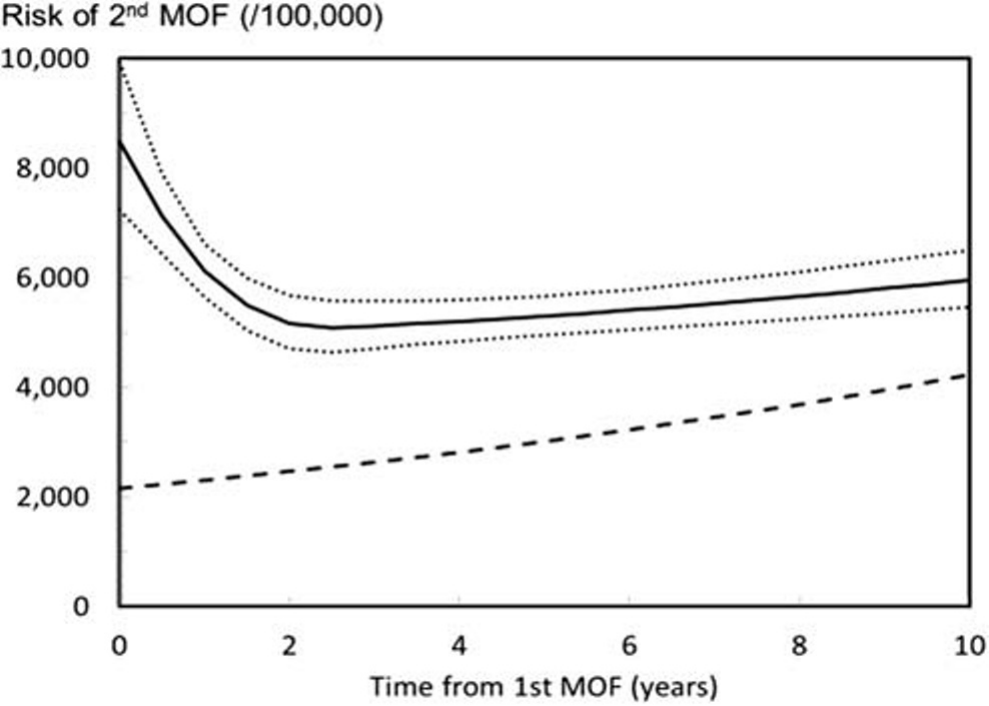
Risk per 100,000 (95%CI) of a second MOF after a first MOF for a woman at the age of 75 years at her first fracture [23]. The dashed line represents the risk of first MOF in the age- and sex-matched population

Individuals who have already suffered a fragility fracture are at a greater risk for further fractures both at the same site and elsewhere. This additional risk of refracture is highest immediately after a fracture [23]. Figure 7 shows the risk per 100,000 women at the age of 75 years following a MOF. The high subsequent fracture risk observed during the first two years following the fracture has been referred to as the period of imminent risk [23, 24]. The existence of an imminent risk period signals that there is an opportunity to optimize the benefits of fracture prevention treatments if patients could be identified and managed as soon as possible after fracture.

Available evidence shows that similar patterns of imminent fracture risk are observed in all countries where this has been explored [25–31]. However, there is little information to assess whether there are differences in imminent fracture risk between countries. Findings from Sweden are given in the Appendix (4: Imminent fracture risk).

The empirical 10-year probability of MOF was consistently higher in those with a sentinel clinical vertebral fracture within the past two years than the FRAX probability in the population of the same age with any previous fracture, but the relative risk (observed/expected probability) varied by age. For example, the relative risk at the age of 50 years for a woman with a clinical vertebral fracture within the previous 2 years was 2.5; for the age of 80 years, the ratio was 1.2 (Table 3).

**Table 3:** Ten-year probability of a major osteoporotic fracture (MOF) for Icelandic women at different ages, categorized by previous fracture [32]

	10-year probability of MOF		
Age	Cohort with clinical vertebral fracture 0–2 years ago	Cohort with any previous fracture in adult life	Ratio
50	29.0	11.7	2.47
60	36.1	19.4	1.86
70	41.9	27.6	1.52
80	42.5	34.2	1.24
90	34.7	33.3	1.04

The impact of the adjustment in the EU6 countries is illustrated in Table 4 which shows the impact of a recent clinical vertebral fracture on conventional FRAX probabilities.

**Table 4:** Ten-year probability of a major osteoporotic fracture (MOF) in women with a prior clinical vertebral fracture at an undetermined time and within the past two years according to country. Age set to 60 years, BMI 25 kg/m^2^, no additional risk factors [32]

	Probability MOF (%)	
Country	Undetermined time	Within the past 2 years
France	9.4	17
Germany	12	22
Italy	12	22
Spain	7.0	13
Sweden	21	39
UK	16	30

Thus, 10-year FRAX probabilities can be adjusted in the presence of a recent vertebral fracture and are likely be useful in treatment decision-making. Similar adjustments for recent fractures at other sites are a requirement for the future.

## Economic cost of fragility fractures

### Fracture costs and length of hospital stay

Fragility fractures incur both short-term and long-term costs for the health care sector and for society. These costs differ between fracture sites, and to some extent reflect the severity of fracture, in particular the need for hospital admission. Hip fractures are the most severe fracture site, and almost always lead to hospitalization and high costs. The length-of-hospital-stay is an important cost component and, within country, has also been shown to have implications for how patients fare over their remaining life time [33].

In the EU6, the average length-of-hospital-stay for hip fracture ranged from 11.6 days in Sweden, to 20.5 days in the UK (Table 5). Methods are given in the Appendix (5: Length of hospital stay).

**Table 5:** Mean length of hospital stay (LOS) and standard deviation (SD) following a hip fracture

Country	LOS (days)		Source
	Mean	SD	
France	12	8.0	[34]
Germany	14.5 (2.6)	2.6	[35]
Italy	19.0 (25.3)	25.3	[36]
Spain	11.8 (7.9)	7.9	[37]
Sweden	11.6 (8.7)	8.7	[33]
UK	20.5 (20.0)	21.6	[38]

The unit fracture costs differed substantially between countries and fracture sites (Table 6). Hip fractures were the costliest fracture type in all countries, whilst distal forearm fractures were the least costly. Fracture costs were generally high in Sweden and Germany, and the lowest in Spain. For more details, see the Appendix (6: Fracture-related costs).

**Table 6:** Mean cost of fracture (€ 2017) in the year following fracture at the sites shown

Country	Hip	Vertebral	Distal forearm
France	12,856	3205	1468
Germany	20,884	11,080	1275
Italy	21,307	4713	1301
Spain	9724	1928	533
Sweden	16,406	14,474	4028
UK	20,650	4028	2568

### Annual fracture-related costs

If current trends in fracture prevention continue, as the general population grows and lives for longer, the hospital and societal cost of fragility fractures will continue to increase.

**Fig. 8:**
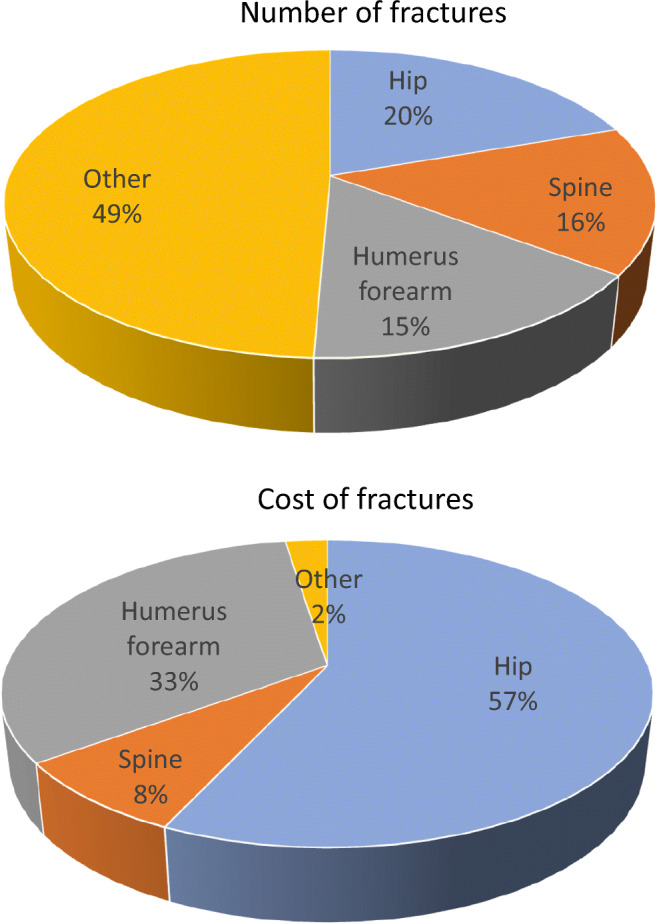
Number and cost of fragility fractures in the EU6 expressed as a percentage of the totals. Note: The estimates conservatively assume no long-term costs for ‘other fractures’

The fracture-related costs in the EU6 amounted to €37.5 billion in the year 2017. Hip fractures accounted for the majority of the total cost (57%) whereas they accounted for 20% of fragility fractures (Fig. 8).

The direct cost of fractures in each EU6 country is given in Table 7. Costs comprise the annual cost of fractures in 2017 (incident fractures), those arising from fractures before 2017 (prior fractures) and the cost of institutional care.

**Table 7:** The direct cost (million Euro) of fractures in 2017 (incident fractures), those arising from fractures before 2017 (prior fractures) and the cost of institutional care in each EU6 country

Country	Incident fractures	Prior fractures	Institutional care	Total
France	3748	219	1404	5371
Germany	8176	414	2680	11,270
Italy	5951	299	3179	9429
Spain	2150	137	1915	4202
UK	2955	372	1919	5246
Sweden	1199	81	690	1970

**Fig. 9:**
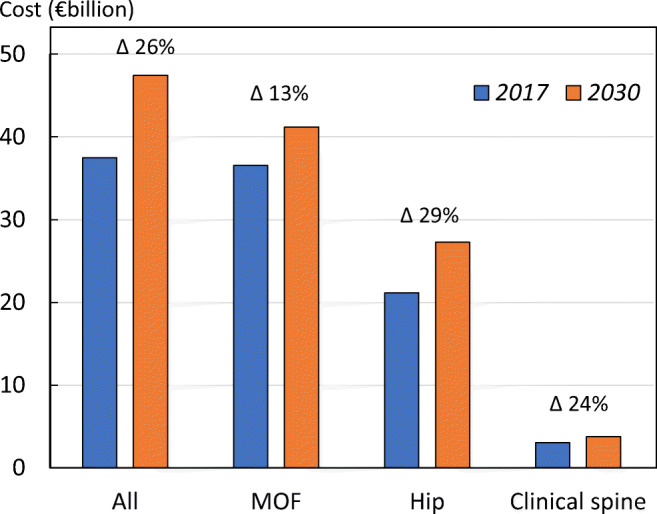
Annual cost of fractures by site in the EU6 for 2017 and projected increase by 2030

In 2010, fracture-related costs in the EU6 were estimated to total €29.6 billion [39]. Fracture-related costs for the EU6 in 2017 were now estimated to total €37.5 billion (an increase of 27% since 2010), and are projected to increase to €47.4 billion in 2030 (an increase of 27% since 2017) (Fig. 9).

**Fig. 10:**
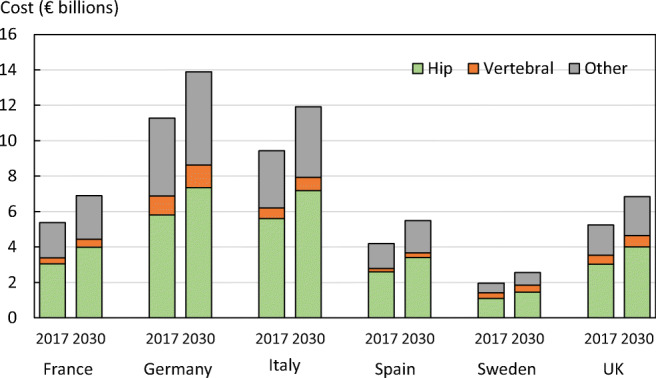
Cost of fragility fractures in 2017 and that expected in 2030 by country and fracture site

As expected, costs will increase due to the increase in fracture cases. The fracture-related costs in the EU6 are projected to increase by 27% from a total €37.5 billion in the year 2017 to €47.4 billion in 2030. Cost projections to 2030 are shown for each country by fracture site in Fig. 10. The dominant cost was for hip fracture. The fracture-related cost estimates provided are conservative, since costs from other fracture sites were not included in the estimation.

There were small variations in the percentage increase in cost by country. The greater increases were noted in Spain (+30.6%), the UK (+30.2) and Sweden (+29.4%) and lower increments in Germany (+23.2%), Italy (+26.2%) and France (+26.4%).

Cost for incident fractures in a given year and long-term cost (due to fractures that arose in previous years), as well as the cost of residing in nursing homes, are detailed in the Appendix (7: Annual cost of fractures).

## Patient burden

### Quality-adjusted life years

The use of QALYs is a method of measuring the burden of a disease where a year of an individual’s life is weighted by the average health-related quality of life (HRQoL) that a person had during that year. For example, 1 QALY is equal to one year spent in perfect health; 0.5 QALYs can be thought of as either half a year spent in perfect health followed by death, or one year lived at 50% of perfect health. QALYs are regularly used in economic analyses because they provide decision makers with a method for quantifying and comparing burden across diseases.

QALYs lost due to fragility fractures were estimated from fracture-based HRQoL, fracture risks and death rates [40–42]. Methods are summarised in the Appendix (8: Quality-adjusted life years). Estimates of the QALY loss were generated from 2017 up to year 2030, based on population projections, to show the expected change in QALY loss for the near future.

**Fig. 11:**
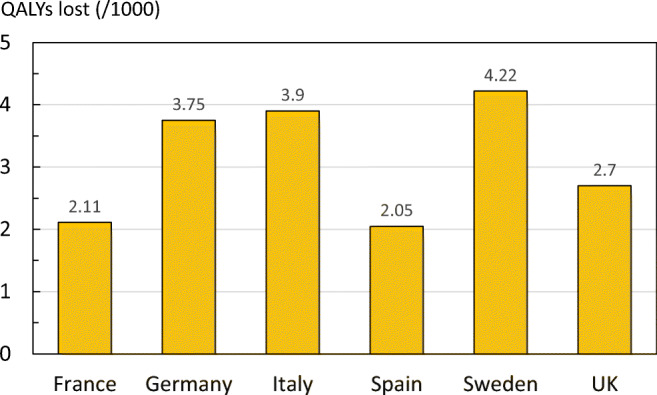
Quality of life years (QALYs) lost in 2017 due to fragility fractures per 1000 of the population age 50 years or more in countries of the EU6

The total health burden in 2017 due to fragility fractures in EU6 was at 1.02 million QALYs. 66% of the QALY loss was due to fractures occurring in women. The QALY loss in absolute numbers was highest in Germany due to the size of the population combined with comparatively high risk of fractures. The lowest QALY loss was observed in Sweden due to the small population size compared to the other countries. On a per capita basis, Sweden had the largest burden (4.22 lost QALYs per 1000 people age 50 years and above) and France the lowest (2.11 lost QALYs per 1000) (Fig. 11). The differences were driven, in large part, by differences in the risk of fractures and age distribution between countries.

**Fig. 12:**
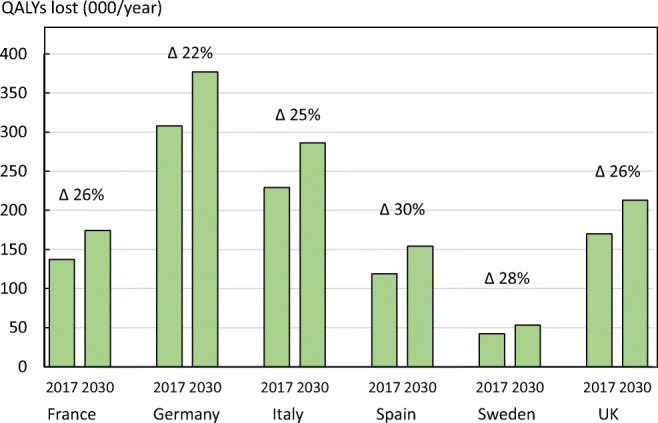
Quality of life years (QALYs) lost due to fragility fractures in countries of the EU6 in 2017 and 2030

The QALY burden is expected to increase by 25.6% in the year 2030 but varied by country (Fig. 12).

### Disability-adjusted life years

The DALY (or disability-adjusted life year) is the World Health Organization’s (WHO) standard method of measuring the burden of a disease. DALYs are the sum of years of life lost (YLL) and the years lost due to disability (YLD) [46]. A single DALY can be thought of as one year of ‘healthy life’ lost. Summing the DALYs across an entire population provides the gap between the current health status of a population and an ideal disease-free population, i.e. the burden [43]. Including this measure of burden allows for comparison of the burden of different diseases, both within and between countries.

When using the WHO standard method, the total DALYs related to fragility fractures in year 2016 for the EU6 (ages of 50 to 100 years) were more than 2.6 million DALYs. Average YLDs per 1000 people (15.1) far exceeded the YLLs per 1000 (5.5), indicating that living with a disability due to fracture drives DALY loss in osteoporosis.

**Fig. 13:**
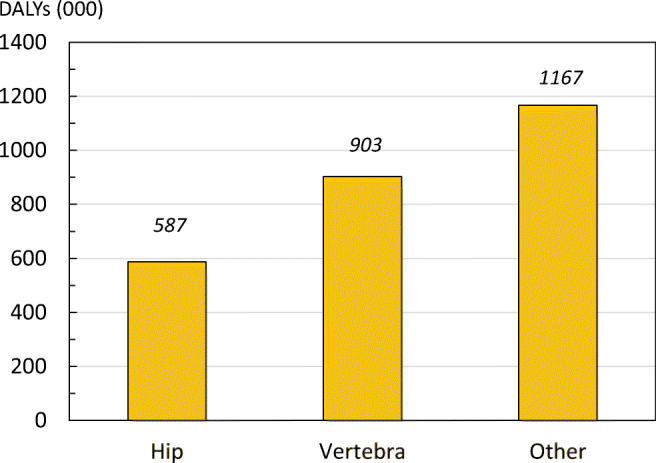
Total DALY distribution by fracture site

The DALY burden was less for hip fracture than for vertebral fracture which, in turn was less than for other fragility fractures (Fig. 13). This dominance of other fragility fractures over hip fractures arose from the combination of a high incidence at early ages, and the large number of years spent with disability from other fractures compared with hip fracture.

**Fig. 14:**
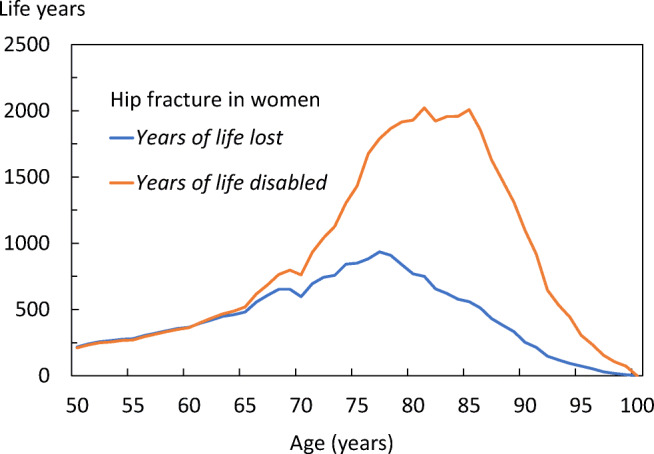
Total DALYs by age for hip fractures in women

**Fig. 15:**
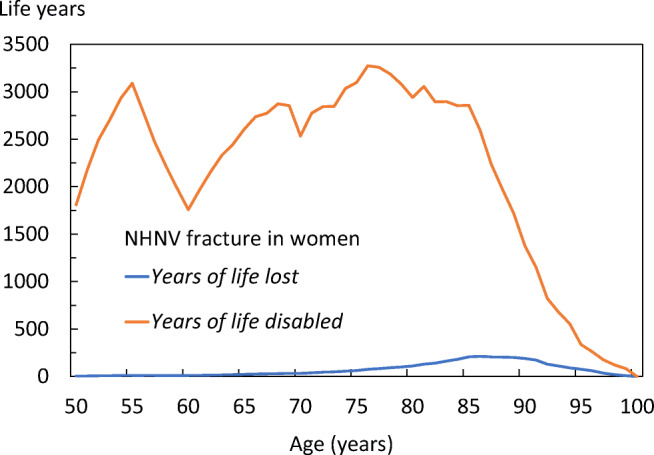
Total DALYs by age for non-hip, non-vertebral (NHNV) fractures in women

The age distributions of YLLs and YLDs differed by fracture site. In women with hip fractures (Fig. 14), the YLLs peaked at the age of 77 years, whilst the YLDs peaked at age 81 years, reflecting that most hip fractures occur around 77 years. The YLDs for non-hip, non-vertebral fractures in the female population (Fig. 15), peaked early and was sustained over age, with very low YLLs, indicating that prevalence of non-hip, non-vertebral (NHNV) fractures is high but with limited consequences for mortality when compared with to that following hip fracture. The equivalent data for men are given in the Appendix (9: Disability-Adjusted life years).

**Fig. 16:**
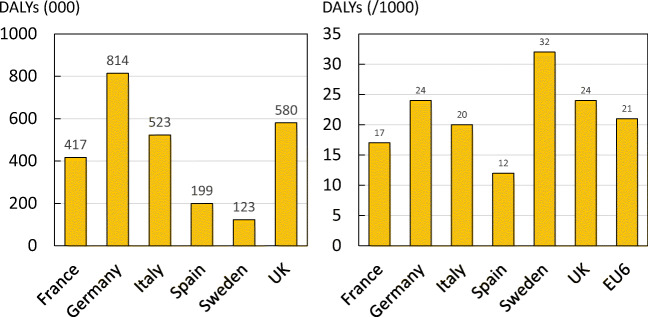
Total DALYs by country (left panel) and DALYs per 1000 individuals by country (right panel)

The total DALY for each country varied greatly due to differences in population demography and fracture risk (Fig. 16). The average DALY loss per 1000 individuals was estimated to be 21 DALYs, with Sweden showing the highest rate (32 DALYs) and Spain showing the lowest (12 DALYs).

**Fig. 17:**
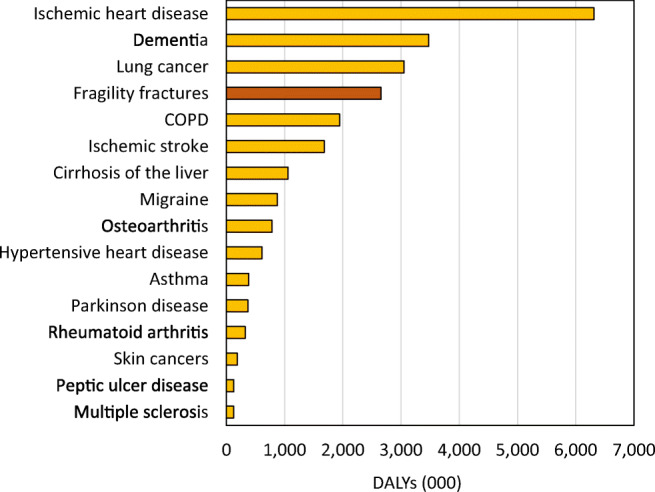
DALYs by disease in EU6 in 17 selected non-communicable diseases

The DALYs related to fragility fractures can be compared to corresponding estimates for other diseases. In Fig. 17, fragility fracture-related DALYs are compared to 16 other common non-communicable diseases in the EU6 [44]. Among these, fragility fractures are placed as the fourth most burdensome, outranked only by ischemic heart disease, dementia and lung cancer.

The DALY burden by disease category varied between countries due to differences in age distribution, risk of fracture and death. The DALY burden also varied by disease category. In Sweden, for example, the DALY burden of fractures was higher than that for dementia whereas in Spain the burden related to dementia, lung cancer and COPD surpassed that for fractures. For more details, see the Appendix (9: Disability-adjusted life years and 11: DALY comparison across diseases)*.* The metrics also provide details of the DALY distribution by fracture site.

From a national perspective, the DALY loss rate can be an important measure for motivating policy decisions and the prioritization of funds towards osteoporosis treatment. From an international perspective, the high values suggest a need for better treatment policy and practice.

### Loss of productivity

Most fragility fractures occur in older retired patients. If, however, individuals sustain a fracture whilst still employed they will likely need to take time off from work to recover from the fragility fracture. In Sweden, for example, about 20% of fractures occur at pre-retirement age [11]. Work absence both impacts the individual’s income and creates a societal cost due to the loss of productivity.

To measure this loss of productivity, data collected in the International Costs and Utilities Related to Osteoporotic Fractures Study (ICUROS) [41, 45, 46] were used to estimate the number of sick days taken by non-retired individuals from the ages of 50 to 65 years in the year following an osteoporotic fracture. Since Germany was not included in the 11 countries that made up the ICUROS study, a combination of the other 5 countries, as well as Austria and Estonia, termed ICUROS Europe, was used as a substitute measure for the EU6. Average sick days were combined with fracture projection data to estimate the total sick days taken due to fragility fractures in 2017, by non-retired individuals. Because there are no appropriate data on the proportion of the population that work beyond the age of 65 years, a retirement age was set at 65 years for all countries in the calculations. For more details, see Appendix (11: Productivity loss).

Hip fractures resulted in the highest number of sick days taken in the first year after fracture (42 days), followed by vertebral fractures (20 days) and other MOFs (12 days). Sick days taken in 2017, by non-retired individuals in the EU6 totalled 7,615,719 days. The other MOFs (distal forearm and proximal humerus fracture) arose more often than hip or clinical vertebral fractures, and therefore resulted in the highest number of sick days.

**Fig. 18:**
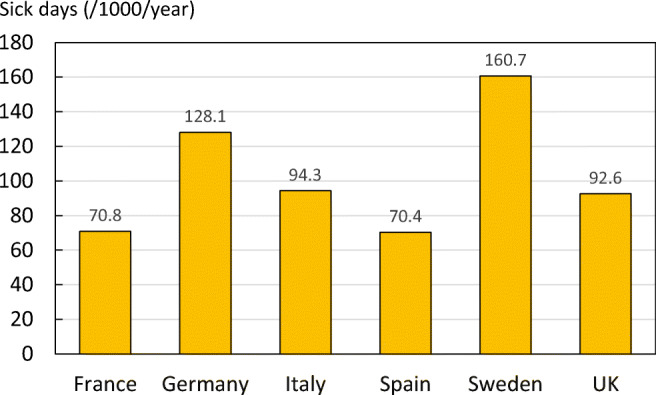
Average sick days taken after fragility fracture per 1000 individuals’ age 50–65 years, by country

When sick days taken due to fragility fracture were expressed per 1000 people age 50 to 65 years in all countries, Sweden had the highest estimate of the EU6 countries (Fig. 18). There were no significant differences between sick leave taken by men and women with hip fractures, nor between sick leave taken by hip fracture patients with or without previous fracture.

### Caregiver burden

Another significant burden associated with fragility fractures and other diseases is the burden imposed on informal caregivers such as family members. Continued care provided at home can put physical, emotional and financial strain on relatives who need to take care of osteoporotic fracture patients [15, 47]. To measure the average burden placed on informal caregivers per year, survey responses from the ICUROS [41, 45, 46] were also used to determine the caregiver burden due to osteoporotic fracture. It was measured in terms of hours of care per year provided by relatives of fracture cases in ICUROS Europe (a substitute measure for the EU6), as well as selected countries. For methods and estimates by fracture type, see the Appendix (12: Caregiver burden).

**Fig. 19:**
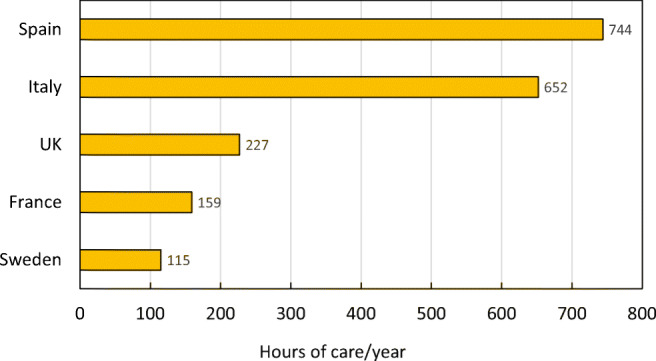
Average annual hours of care by relatives after hip fracture by country

Hip fractures were associated with the largest caregiver burden (370 h per year), followed by vertebral fractures (263 h per year) and other MOFs (130 h per year). Hours of care provided by relatives varied greatly by country. In countries where cross-generational support is more established, the impact of fragility fractures on caregivers is generally higher [48]. Accordingly, Spain and Italy had the highest caregiver burden, with averages of 756 h and 882 h a year, per 1000 individuals, spent caring for patients with osteoporotic hip fractures, respectively. France (138 h) and Sweden (191 h) had considerably lower averages (Fig. 19). There were no significant differences in care from relatives between men and women, nor between patients with or without a previous fracture.

### Independent living

One major burden caused by fragility fractures is the long-term impact on independence. The fracture can result in a loss of mobility, the ability to take care of oneself, and may require the individual to move into long-term care (LTC) or care services [49]. The ICUROS provided survey responses for the percentage of individuals who needed to move into LTC as a direct result of an osteoporotic fracture. For methods, see the Appendix (13: Independent living).

**Fig. 20:**
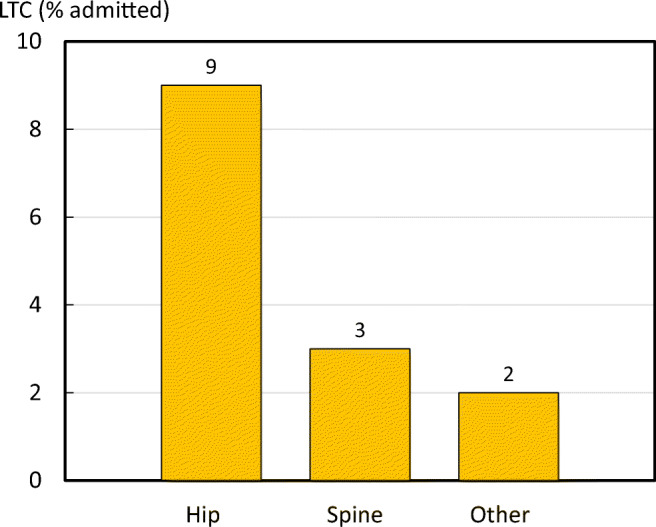
Percentage of patients admitted to long-term care (LTC) within 12 months after a fracture by fracture site (ICUROS Europe). Other refers to other fragility fractures

LTC use varied greatly, depending on the fragility fracture and the age of the individual. Hip fractures result in the largest proportion of people moving to LTC in ICUROS Europe (Fig. 20).

**Fig. 21:**
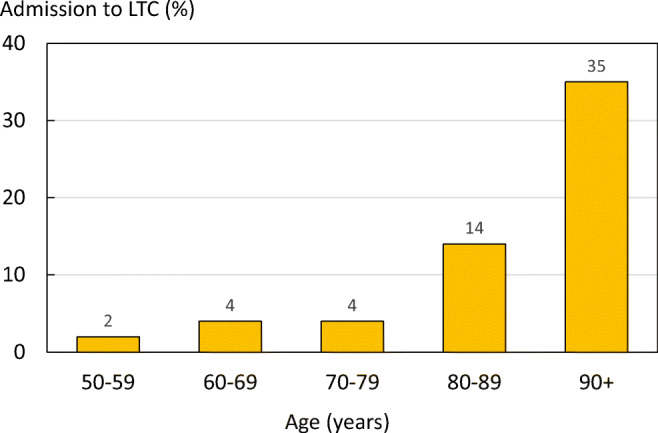
Percentage in long-term care (LTC) at one year after hip fracture, by age group

The percentage of patients moving into LTC following a hip fracture increased significantly with age, from 2.1% at ages 50–60 years to 35.3% at ages 90–100 years (Fig. 21).

## Fracture prevention

### Pharmacological treatment gap

The treatment gap (i.e. the number of women that are treated compared to the proportion of the population that could be considered eligible for treatment) in osteoporosis has been estimated for the European Union using international sales data on volume (standard units) and price (€) from IMS Health for year 2010 [15, 50]. Applying the same methodology, an update of the treatment gap was conducted using IMS sales data for year 2017. The analysis included data on sales related to all osteoporosis drugs (bisphosphonates, denosumab, parathyroid hormone and peptides, selective oestrogen receptor modulators (SERMs) and strontium ranelate). Menopausal hormone treatment (MHT) was not included.

The treatment gap was estimated from the difference between the number of patients treated with an osteoporosis drug using IMS sales data and the number of patients in the population considered to be eligible for an osteoporosis treatment. Further details are given in the Appendix (14: Pharmacological treatment gap). In line with European guidelines [51], patients eligible for treatment have a country- and age-specific MOF fracture probability equivalent to a woman with a prior fragility fracture based on the FRAX algorithm. The calculation of the treatment gap assumes that all treatments are given to patients above the intervention threshold. The approach does not take account of differences in treatment guidelines between countries.

**Fig. 22:**
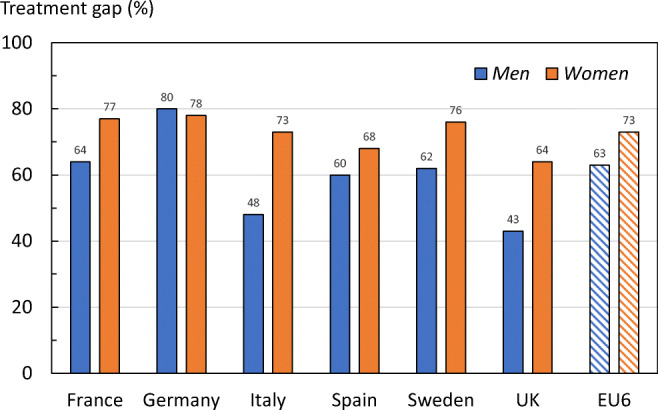
Treatment gap in men and women by country in 2017

The average treatment gap (percent eligible patients not treated) in EU6 in year 2017 was 73% for women and 63% for men (Fig. 22). The higher gap in women was the case in all countries with the exception of Germany which had the highest treatment gap. Only 20% of eligible men and 22% of women in Germany would receive a pharmacologic intervention. The treatment gap varied between countries. The highest treatment gap for women was in Germany, whereas the UK had the smallest treatment gap (64%) in women and in men (43%).

**Fig. 23:**
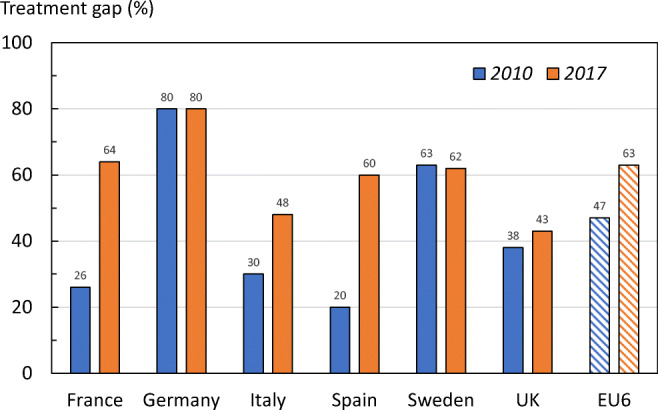
The treatment gap (percent eligible patients not treated) in men from the EU6 in 2010 and 2017

**Fig. 24:**
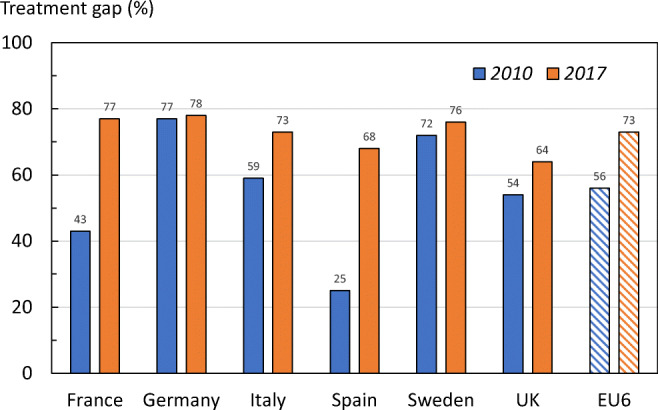
The treatment gap (percent eligible patients not treated) in women from the EU6 in 2010 and 2017

Changes in the treatment gap between 2010 and 2017 are shown for men (Fig. 23) and women (Fig. 24). Compared to the analysis from year 2010, there was a marked increase in the treatment gap for the EU6 (17% and 16% points for women and men, respectively). This increase was mainly driven by large changes in France and Spain. The adverse changes in treatment gap were most marked in France (38 percentage points increase in men and 34 percentage points in women), and Spain (by 40 and 43 percentage points increase in men and women, respectively). The treatment gap increased to a lesser extent in Italy and was relatively stable in Germany, Sweden and the UK.

### Post-fracture treatment gap

**Fig. 25:**
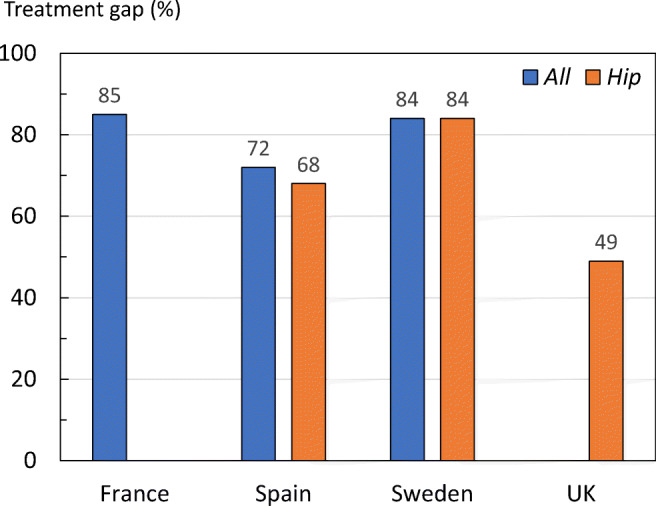
Percentage (%) of women (50 years and above) not treated within a year of an osteoporotic fracture or a hip fracture

An alternative approach for assessing the treatment gap is to estimate the proportion of patients starting a pharmacological treatment after a fracture. Available estimates were gathered from a mix of literature, public reports (France [52] and the UK [53]), data on file at UCB (Spain) and data on file at Quantify Research (Sweden). The percentage of women who did not receive osteoporosis-specific pharmacological treatment within a year of an osteoporotic fracture is shown in Fig. 25. The analytic methods vary between the estimates making direct comparisons difficult. However, the post-fracture treatment gap can be considered large irrespective of country. With the exception of the UK, no more than 30% of women receive a treatment following a fracture. In the UK, the treatment gap was markedly lower after hip fracture (49%). For more details, see the Appendix (15: Fracture treatment gap).

A more detailed analysis, using the Swedish National Patient Register (NPR) and the Swedish National Prescription Register, was conducted to explore differences in the treatment gap for different subpopulations. Patients were defined as treatment-naïve if they had not collected any prescriptions for anti-osteoporotic medications during the three years prior to the fracture.

**Fig. 26:**
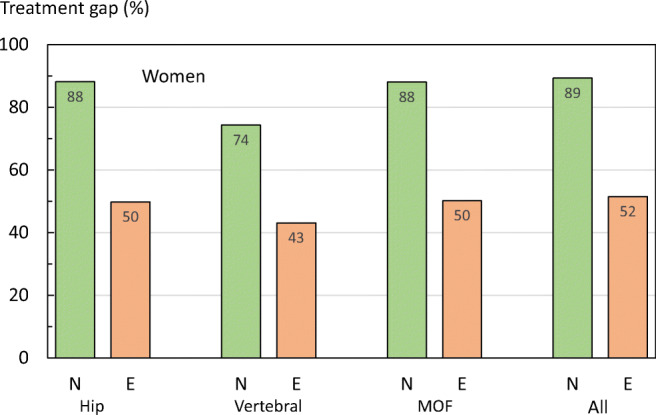
Percentage of women untreated within one year of fracture by site of fracture and prior exposure to osteoporosis treatment in Sweden. N, treatment-naïve; E, prior exposure

**Fig. 27:**
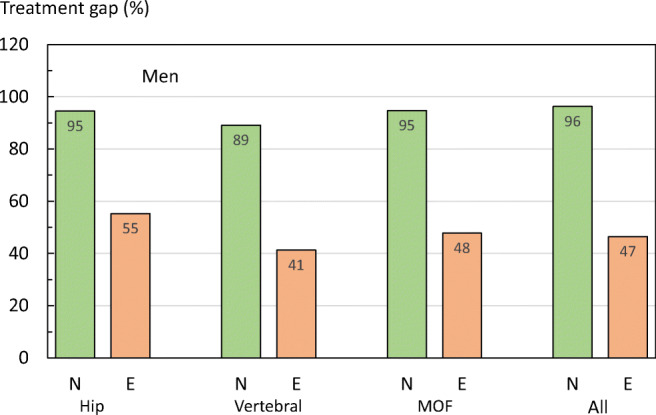
Percentage of men untreated within one year of fracture by site of fracture and prior exposure to osteoporosis treatment in Sweden. N, treatment-naïve; E, prior exposure

At the time of fracture, most women (89%) and men (97%) were treatment naïve. Figure 26 shows the pattern of treatment following a fracture by treatment exposure in women. Within the year following a hip fracture, a MOF or any fragility fracture, only 11% to 12% of treatment-naïve women started treatment for osteoporosis. Following a vertebral fracture, 26% of treatment-naïve women started treatment. A similar pattern was observed in the male population although treatment gaps were in general higher (Fig. 27). About 5% of treatment-naïve men were treated following a hip fracture, or a MOF. Following a vertebral fracture, 11% of treatment-naïve men started treatment.

In men and women who had previously been exposed to therapies for osteoporosis, the treatment gap was substantially lower than in treatment-naïve patients. These finding illustrate important issues in that a new treatment is rarely offered to (or taken up by) patients after fracture and, even in patients previously exposed to osteoporosis treatment, only about half receive a treatment within the next year.

A limitation of this analysis is that the Swedish National Patient Register (SNPR) does not cover drugs dispensed at the hospital (mainly intravenous and subcutaneous administered medications), which are estimated to comprise 4% of medicines sold [54]. This likely leads to a slight overestimation of the treatment gap. For more details, see the Appendix (16: Treatment gap by fracture type).

### Fracture risk assessment

Although osteoporosis is defined in terms of BMD, there are several other factors that are associated with an increased risk of fracture that are not captured by BMD. This has led to the development of risk models, which incorporate several risk factors to improve the identification of patients at high risk [55].

There are several existing models for risk assessment in Europe; however, the most widely used is FRAX [56]. FRAX, released in 2008, is a computer-based algorithm that calculates the probability of fracture in individuals using age, body mass index, BMD (optionally) and risk factors such as whether the patient had a prior fragility fracture, their parental history of hip fracture, whether they smoke, drink, have rheumatoid arthritis and other factors that increase the risk for osteoporosis [57]. FRAX models are currently available for 68 countries and are publicly available on the official FRAX website [58]. There are also several other fracture risk assessment models available.

Table 8 provides a summary of the access to FRAX and other risk assessment models in the EU6. Country-specific FRAX models exist in all 6 countries. Alternative assessment models are also recommended for use in Germany, Italy and the UK. The German DVO model, developed in 2006, is a Germany-specific risk assessment model which requires the use of BMD measurements [59, 60]. DeFra is an Italy-specific extension of the FRAX model, which allows for comparison of the BMD in different fracture sites and the inclusion of more variables [61]. QFracture® in the UK was developed in 2009, and uses variables that are available through healthcare records in the UK; it does not include BMD [62]. For more details, see the Appendix (18: Fracture risk assessment).

**Table 8:** Risk models and guidelines available in the countries of interest

Countries	FRAX model available	Other models	National guidance	Comments	Source
France	Yes	–	Yes		[63]
Germany	Yes	DVO Model	Yes		[59]
Italy	Yes	FRAHS, DeFra	Yes	FRAHS: FRAX-based	[64–66]
Spain	Yes	–	Yes		[67]
Sweden	Yes	–	Yes		[68]
UK	Yes	QFracture	Yes		[69]

Specific guidelines for the use of FRAX and other risk models are noted on official national health service websites for all countries except for Italy. The Italian Ministry of health does not recommend specific risk models but suggests that risk models may be useful in assessing the probability of fragility fracture. Other organizations like the Italian Society for Orthopaedics and Traumatology recommended FRAX or DeFra.

**Fig. 28:**
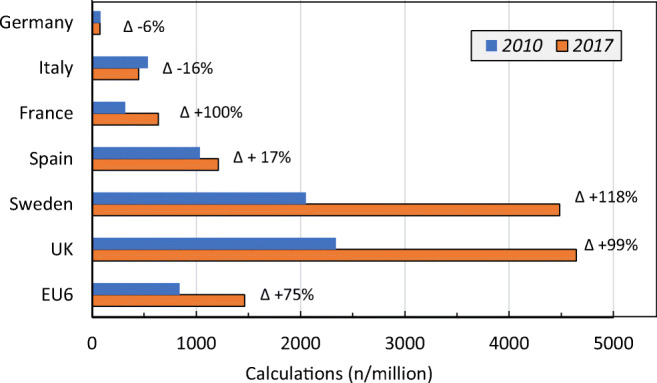
FRAX calculations by URL source per million in the general population November 2010– October 2011 and April 2017– March 2018

The uptake of FRAX in 2010 and 2017 is shown in Fig. 28 as the number of calculations/million persons in the general population. The UK and Sweden had the highest usage of FRAX, whereas the lowest uptakes were seen in Germany and Italy. Considering all countries in the EU6, the usage use of FRAX increased by almost 74% in 2017 compared to 2010. The highest increase was seen in the UK, France and Sweden (~ 100%), whereas in both Germany and Italy, the usage of FRAX decreased was reduced in 2017 compared to 2010. In both Germany and Italy, the usage of FRAX decreased in 2017 compared to 2011. The decrease in the use of FRAX in both Italy and Germany may relate to the availability of other risk models such as the German specific DVO model and DeFra in Italy. For more details, see the Appendix (18: Use of FRAX).

### Fracture liaison services

A fracture liaison service (FLS) is a multi-disciplinary health care delivery model for secondary fracture prevention. FLS aims to systemically identify, treat and refer all eligible patients within a local population who have suffered a fragility fracture with the aim of reducing their risk of subsequent fractures. The FLS concept was first introduced in teaching hospitals in Scotland and has grown in popularity around the world due to its effectiveness in preventing secondary fractures [70]. A growing body of published evidence suggests that FLSs are a cost-effective care delivery model that has the potential to reduce the risk of refracture, increase the number of high-risk patients being treated and improve adherence to treatment. [71–77].

A recently published systematic literature review and meta-analysis based on 159 scientific publications studied several important outcomes of fracture liaison services [78]. Albeit, with a variety of study designs used, all the studies attempted to estimate the impact of a FLS compared to the absence of such a program. The meta-analysis indicated that FLS improved the rate of fractured patients getting BMD tests, starting treatment and adhering to treatment by about 20% (Table 9). The results also showed a significant reduction in the refracture rates.

**Table 9:** Meta-analysis results for outcomes of FLS [78]

Outcome measure	Effect of FLS (absolute change)	95% CI	Duration of follow-up (months)	Number of studies
BMD testing	+ 24%	(0.18 to 0.29)	3–26	37
Treatment initiation	+ 20%	(0.16 to 0.25)	3–72	46
Adherence	+ 22%	(0.13 to 0.31)	3–48	9
Refracture	− 5%	(− 0.08 to − 0.03)	6–72	11

Even though the meta-analysis showed an overall positive impact of FLSs, it did not consider that there are different types of FLS models which is likely to be associated with different outcomes. For example, some FLS only identify patients and inform them without taking any further actions whereas other more complete FLS identify, investigate, treat and monitor the patient. In another recent study, the evidence of different FLS model types (A to D) on fracture risk, DXA referrals, and other patient outcomes were reviewed [77]. The most complete FLS model (type A) was associated with reduction in refracture risk (hazard ratio [HR] 0.18–0.67 over 2–4 years), increased assessment of BMD (relative risk [RR] 2–3), increased treatment initiation (RR 1.5–4.25) and adherence to treatment (65–88% at 1 year).

Along with the literature focusing on the impact of FLSs, several studies have analysed the cost-effectiveness and cost savings of providing FLSs. Estimates in Sweden and the UK for the economic impact of FLSs are shown in Table 10. For more details, see the Appendix (19: Fracture liaison service impact).

**Table 10:** Country-specific studies on the economic impact of FLS

Country	Type	Estimate	Source
Sweden	ICER (cost-eff)	€ 14,029 (per QALY gained)	[79]
UK (hip patients)	ICER (cost-eff)	€22,700-€26,600 (per QALY gained)	[80]
UK	Cost savings	€23,800/lifetime/1000 patients	[74]

The large variation between different types of FLS and their evaluation complicates the assessment of the overall benefits of FLS and merits of a specific FLS model. Initiatives that promote standardised outcome frameworks for assessing FLS and increased collaboration between providers include the Capture the Fracture® and the UK FLS-Database Audit [81, 82].

### Capture the Fracture®

One effort to encourage cooperation between FLS providers is *Capture the Fracture® (*CtF), a global initiative of IOF to ‘facilitate the implementation of coordinated, multi-disciplinary models of care for secondary fracture prevention’ [73]. CtF has created a set of internationally endorsed standards and guides for best practice and has assembled the largest network of individual FLS providers in the world. CtF provides resources, tools and educational programmes to bridge the gap between FLS providers and helps in the creation of new FLS.

This growing network of FLS providers is mapped on their website (https://www.capturethefracture.org/map-of-best-practice-page)spain and provides a rating of the existing service providers in a given area. To be included in the CtF network, the provider must undergo a standardised external audit to determine the quality of their services. Table 11 shows the star ratings for registered FLS providers in the countries of interest. A value of 4, 3 and 2 was applied to gold, silver and bronze, respectively and a 1 to providers currently under review. Spain and the UK lead in terms of the number of registered FLS, whereas Spain, the UK and Sweden score highly in the average score/FLS.

**Table 11:** Number of Capture the Fracture FLS ratings by country and scores [73]

Country	Total	Gold	Silver	Bronze	Other	Score	Score/FLS
France	20	0	3	9	8	35	1.75
Germany	2	0	1	0	1	4	2.0
Italy	13	1	3	2	7	24	1.8
Spain	65	13	13	22	17	152	2.3
Sweden	5	0	4	1	0	14	2.8
UK	25	6	11	1	7	66	2.6
EU6	130	20	35	35	40	285	2.2

There is currently no publicly available information on how many fragility fractures are referred to an FLS within the EU6 countries. A survey sent to a selected number of FLSs in the EU6, enrolled in IOF’s *Capture the Fracture* network, asked for the percentage of hospitals and general practitioners (GPs), on a national level, that have a system to refer fractured patients. The responses varied between an average of 2.8% in Italy, to 37.5% in Sweden for hospital referrals and 1–10% for GP referrals. In the UK, the National Osteoporosis Society has estimated that 55% of the UK population has access to a FLS. For more details, see the Appendix (20: Capture the fracture).

### Closing the FLS gap

Given the available evidence showing the potential benefits of FLSs and the sub optimal coverage of such models in the EU6, it is as relevant to highlight the FLS treatment gap. When applying the information on fracture epidemiology, costs, current FLS coverage previously described in this report and evidence of FLS outcomes based on Wu et al. [78], it is possible to assess the potential impact a complete coverage of FLS could have on the burden of fragility fractures.

It is estimated that, 19,262 number of subsequent fragility fractures could be avoided every year by extending the access to FLS for all citizens above 50 years of age in EU6. The reduction in the annual fracture-related cost associated with these fractures is €285.4 million. Adding the additional cost related to increased FLS resources and drug administration the net impact is an increased cost of €39.7 million but at a gain of 8858 quality-adjusted life years (Table 12). The cost per QALY gained of an FLS extension would be €3108, an estimate that can be considered cost-effective in all countries and probably underestimated because of conservative assumptions on the costs related to other osteoporotic fractures. The variation between countries is mainly driven by differences in fracture risk and cost of osteoporosis drugs.

**Table 12:** Potential reduced burden by closing the FLS gap

Country	Fractures avoided (per year)	Fractures avoided per 1000 FLS patients	Reduction in annual fracture-related cost (million €)	Net impact on annual burden (million €)	Net impact per patient (€)	Reduction in annual burden (QALYs)
France	2665	10.0	−38.0	20.0	75.0	1036
Germany	5423	13.9	−75.4	8.2	21.0	2335
Italy	2868	7.2	−55.7	−4.8	−12.0	1602
Spain	1249	5.4	−18.4	20.0	86.0	584
Sweden	1371	22.7	−22.4	−2.3	−38.0	596
UK	5686	16.2	−75.5	−1.4	−4.0	2705
EU6	19,262	11.3	−285.4	39.7	16.2	8858

## Executive summary

Osteoporosis is a disease that weakens the bones and increases the risk of fragility fractures, where bones can break from a fall from a standing height or less. In Western Europe, about 1 in 3 women and 1 in 5 men at or above the age of 50 years will fracture during their lifetime. The number of fragility fractures and cases of osteoporosis is increasing worldwide, creating an increasing burden to society.

This report provides an overview and a comparison of the burden and management of fragility fractures in six European countries (France, Germany, Italy, Spain, Sweden, UK), hereafter referred to as EU6.

### Key findings

- The total number of fragility fractures in the EU6 is estimated to increase from 2.7 million in 2017 to 3.3 million in 2030; an increase of 23.3%.
- The annual fracture-related costs in the EU6 are projected to increase from a total €37.5 billion 2017 to €47.4 billion in 2030; an increase of 27%.
- The number of disability-adjusted life years (DALYs) per 1000 individuals’ age 50 years or more in EU6 due to fragility fractures was estimated at 21 years. This is a higher estimate compared to some other chronic diseases such as stroke (13 DALYs per 1000) and chronic obstructive pulmonary disease (COPD) (15 DALYs per 1000).
- The risk of refracture is highest immediately after a fracture. This has been referred to as the period of imminent risk; this phenomenon suggests that there is an opportunity to optimize the benefits of fracture prevention by treating patients as soon as possible after occurrence of a fracture.
- The treatment gap (defined as the percent eligible individuals not receiving treatment with osteoporosis drugs) in EU6 in year 2017 is estimated to be 73% for women and 63% for men. Compared to analysis from the year 2010, this is a marked increase from 56% in women and 47% in men.
- The proportion of patients starting a pharmacological treatment in the year after a fracture is low. In France, Sweden and Spain, 85%, 84% and 72% of fracture patients remained untreated 1 year after fracture, respectively.
- A fracture liaison service (FLS) is a multi-disciplinary health care delivery model for secondary fracture prevention. This health care delivery model has become more common in recent years, but its coverage is still low.
- A growing body of evidence suggests that FLS are cost-effective care delivery models that have the potential to increase the number of high-risk patients being treated, improve adherence to treatment and reduce the risk of refracture.
- A FLS provides an opportunity to improve early post-fracture patient identification and reduce the treatment gap.
- If FLS could be further expanded to reach all fracture patients in the EU6, 19,262 additional fractures every year would be avoided, and fracture-related costs would be reduced by €285.5 million.

### Key results by country

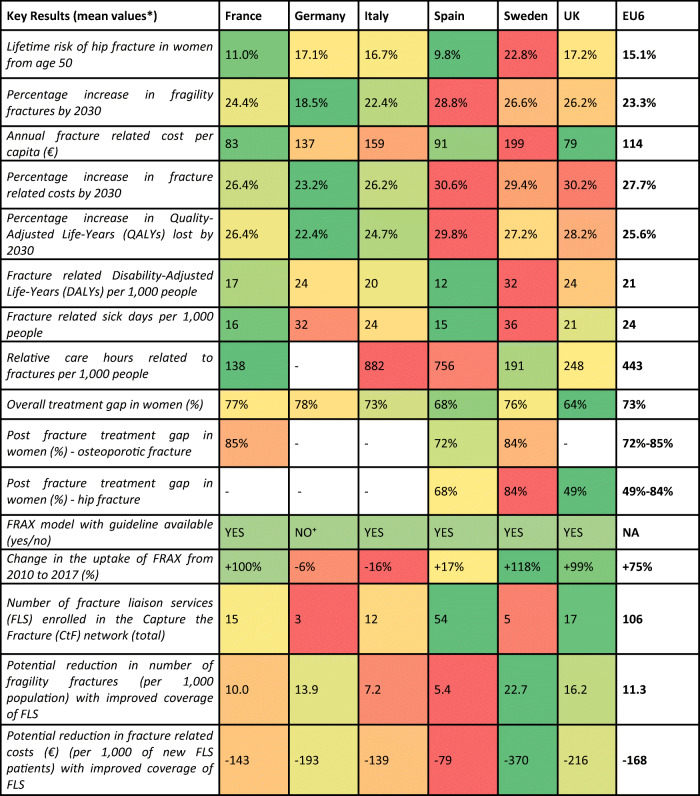



*Mean value if not otherwise stated

^+^FRAX is available in Germany, but no guideline currently endorses its use

Colours indicate ranking among countries (from green = best to red = worst)

## Electronic supplementary material

ESM 1 (DOCX 999 kb)

## Metrics

The accompanying supplementay material contains the individual metrics which served as the background research and evidence for the above report. The metrics include details about the estimation and reporting methods as well as additional research and source material.

## Consultation panel

### France

Bernard Cortet: University Hospital Lille, France
Thierry Thomas: Rheumatology Department, University Hospital St. Etienne, France
Laurent Grange: Department of Rheumatology, University Hospital Grenoble, France

### Germany

Claus Glüer: Department of Radiology and Neuroradiology, University Medical Center Schleswig-Holstein, Kiel University, Germany
Andreas Kurth: Department of Traumatology, Orthopedics and Hand Surgery, Community Hospital Mittelrhein gGmbH, Germany
Peyman Hadji: Department of Bone Oncology, Endocrinology and Reproductive Medicine, Krankenhaus Nordwest, Germany
Thorsten Freikamp: Federal Self-help Association for Osteoporosis (BfO), Germany

### Italy

Maria Luisa Brandi: Department of Endocrinology and Metabolic Diseases and Director of the Operative Unit of Diseases of Mineral and Bone Metabolism, Medical School, University of Florence, Italy
Stefano Gonnelli: Department of Internal Medicine and Director of the School of Specialization in Emergency Medicine and Urgency, University of Siena, Italy
Giuseppe Sessa: Department of Orthopedics and Traumatology and Orthopedic Clinic of the Vittorio Emanuele Polyclinic, University of Catania, Italy

### Spain

Josep Blanch Rubio: Department of the Institut Blanch de Reumatologia, Spain
Adolfo Diez-Perez: Department of Internal Medicine at the Hospital del Mar, Autonomous University of Barcelona, Spain
Maria A Robles Palacios: Asociación Española con la Osteoporosis y la Artrosis, Spain
Santiago Palacios: Instituto Palacios, Salud y Medicina de la Mujer, Spain

### Sweden

Mattias Lorentzon: Department of Geriatric Medicine, Institute of Medicine, University of Gothenburg, and Osteoporosis Clinic at the Sahlgrenska University Hospital, Sweden
Lisa Keisu Lennerlöf: Osteoporosforbundet, Swedish Osteoporosis Association, Sweden

### UK

Cyrus Cooper: MRC Lifecourse Epidemiology Unit, University of Southampton, UK and Professor of Musculoskeletal Science at the NIHR Musculoskeletal Biomedical Research Unit, University of Oxford, UK.
Fizz Thompson: National Osteoporosis Society, UK
Celia L Gregson: Musculoskeletal Research Unit, Bristol Medical School, University of Bristol, UK
