# Supplementary material for: Fragility fractures in Europe: burden, management and opportunities
Source: Arch Osteoporos. 2020 Apr 19;15(1):59. doi: 10.1007/s11657-020-0706-y (PMC7166207; doi:10.1007/s11657-020-0706-y)
Supplement: Supplementary file 1 — (DOCX 999 kb) [file 11657_2020_706_MOESM1_ESM.docx]

**Appendix of metrics**

This appendix contains the individual metrics which served as the background research and evidence for the main report. The metrics include details about the estimation and reporting methods as well as additional research and source material.

**Metric Summary**

A list of the metrics detailed in this appendix are given below, which served as the background research and evidence for the main report. Click on the metric title to jump to the specific metric.

|  | Metric Title | **Short description** | **Outcome(s) units** |
| --- | --- | --- | --- |
| 1. | Prevalence of osteoporosis | Calculated estimates for the current osteoporosis prevalence in the EU6 by gender and age | No. people with osteoporosis, % of people age 50+ with osteoporosis |
| 2. | Lifetime risk of fragility fractures | Micro-simulation model estimates fracture risk for men and women aged 50+, by country and fracture type | % of people who will fracture in their lifetime |
| 3. | Fracture projections | Current and projected estimates for the number of fractures by country and fracture type from 2017 to 2030 | Number of fractures |
| 4. | Imminent fracture risk | Swedish register data shows where the risk of subsequent fracture is highest, by gender | Hazard ratio for the risk of subsequent fracture |
| 5. | Length of hospital stay | Swedish register data on the average lengths of hospital stay after a fragility fracture by gender and fracture type | Number of days spent hospitalized after fracture |
| 6. | Fracture-related costs | Estimates from literature for average the cost of fractures and hospital stay lengths, by country, fracture type and gender | No. days in hospital after fracture, cost of fracture (€) |
| 7. | Annual cost of fractures | Estimates from literature for the current and projected annual costs related to fragility fractures, from 2017 to 2030, by country and fracture type | Annual total costs from fractures (€), % of costs by fracture type |
| 8. | Quality-Adjusted Life-Years | A burden model is used to estimate the QALY loss in each country by gender and fracture type | Annual QALY’s lost due to fragility fractures |
| 9. | Disability-Adjusted Life-Years | The DALYs associated with osteoporosis were calculated by combining the years lost due to disability and the years of life lost, by country, fracture type and age | Total DALYs associated with fragility fractures in 2016, DALYS per 1000 people in the general population |
| 10. | DALY comparison across diseases | DALYs due to osteoporosis were compared to DALYs from other diseases, taken from IHME data | Total DALYs in 2016, DALYS per 1000 people in the general population |
| 11. | Productivity loss | Estimates from observational study data (ICUROS) for the number of sick days taken after a fracture, by country and fracture type | Number of sick days taken per year after a fracture |
| 12. | Caregiver burden | Estimates from observational study data (ICUROS) for the average hours of informal care provided after a fracture, by country and fracture type | Hours of informal care provided per year after a fracture |
| 13. | Independent living | Estimates from observational study data (ICUROS) for the percentage of fracture patients in long-term care after a fracture, by country, fracture type, gender and age | % of patients in long-term care by 12 months after fracture |
| 14. | Pharmacological treatment gap | Using IMS sales data, estimates were derived for the % of eligible individuals who don’t receive treatment after fracture in the EU6 countries | % of fracture patients who do not receive treatment |
| 15. | Fracture treatment gap | A structured literature review provided estimates for the country-specific treatment gap for patients with hip fractures or any fragility fracture | % of fracture patients who do not receive treatment |
| 16. | Treatment gap by fracture type | Swedish register data were used to estimate the treatment gap after a fracture, by fracture type and gender | % of fracture patients who do not receive treatment |
| 17. | Fracture risk assessment | Discusses the availability of fracture risk assessment models such as FRAX in the EU6 | Indicator for risk-model availability |
| 18. | Use of FRAX | Estimates for the change in FRAX risk model usage in the EU6, from 2011 to 2017, based on the number of calculations per million in the general population | Number of risk assessment calculations per million in the general population |
| 19. | Fracture Liaison Service impact | Estimates from literature for the impact of FLS on patient outcomes, and the cost effectiveness of FLS | % change in procedures, re-fracture and mortality, duration of follow-up (months), cost (€) per QALY gained by using FLS |
| 20. | Capture the Fracture | Discusses the design of a network known as Capture the Fracture which catalogues and reviews fracture liaison service (FLS) providers | Number of FLS, FLS rating score |

**1: Prevalence of Osteoporosis**

**Background**

The operational definition of osteoporosis is based on the T-score for bone mineral density (BMD) in women [1] and is defined as a value for BMD 2.5 SD or more below the young female adult mean (T-score less than or equal to –2.5 SD). BMD is usually measured using dual-energy X-ray absorptiometry (DXA). The reference site for measurement is at the femoral neck [2]. The aim of this metric was to assess the prevalence of osteoporosis in France, Germany, Italy, Spain, Sweden and the UK, jointly referred to as EU6.

**Methods**

Ideally, osteoporosis prevalence would be based on country-specific BMD data. In absence of such data in all relevant countries, it is assumed that the mean femoral neck BMD is similar across countries at the age of 50 years and so too is the rate of bone loss with age. [2, 3]. These assumptions have been used elsewhere [4-8]. The prevalence estimates were then applied to the population demography in each EU6 country, comprising approximately 131 million people aged 50 years or older [9].

**Results**

In 2015, there were 20 million individuals with osteoporosis in the EU6 (Fig. A 1). Of those, 15.8 million were women and 4.2 million were men. Country-specific prevalence estimates for individuals aged 50 years and older in women ranged from 21.8% to 23.1%, with an EU6 mean of 22.5% (Table A 1). For men, the prevalence ranged from 6.7% to 7.0%, with an EU6 mean of 6.8%. The prevalence of osteoporosis was highest in Italy, for both men (7.0%) and women (23.1%). Table A 2 presents the number (in thousands) of men and women with osteoporosis in the EU6.


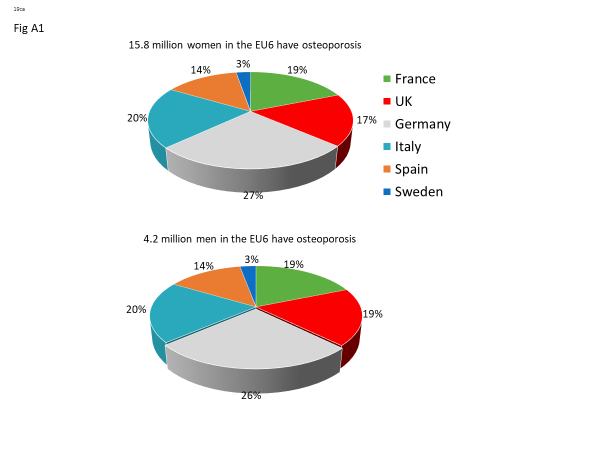


**Fig. A1** The prevalence of osteoporosis in the EU6 [2, 3, 9].

**Table A 1** Prevalence (%) of osteoporosis for men and women age 50 years and older per country

| **Country** | **Women (%)** | **Men (%)** |
| --- | --- | --- |
| France | 22.7 | 6.9 |
| UK | 21.8 | 6.8 |
| Germany | 22.5 | 6.7 |
| Italy | 23.1 | 7.0 |
| Spain | 22.5 | 6.8 |
| Sweden | 22.5 | 6.9 |
| **EU6** | **22.5** | **6.8** |

**Table A 2** Number (in thousands) of men and women (M=men, W=women) with osteoporosis according to age in the EU6 using female-derived reference ranges at the femoral neck

|  | France | | UK | | Germany | | Italy | | Spain | | Sweden | | EU6 | | |
| --- | --- | --- | --- | --- | --- | --- | --- | --- | --- | --- | --- | --- | --- | --- | --- |
| Age | W | M | W | M | W | M | W | M | W | M | W | M | W | M |  |
| 50-54 | 137 | 54 | 147 | 57 | 215 | 88 | 150 | 58 | 112 | 44 | 19 | 8 | 780 | 308 |  |
| 55-59 | 201 | 69 | 194 | 69 | 289 | 105 | 200 | 70 | 152 | 54 | 28 | 10 | 1,064 | 377 |  |
| 60-64 | 291 | 110 | 257 | 100 | 374 | 143 | 275 | 104 | 190 | 73 | 39 | 16 | 1,426 | 546 |  |
| 65-69 | 396 | 134 | 380 | 132 | 439 | 151 | 386 | 128 | 251 | 83 | 62 | 22 | 1,913 | 650 |  |
| 70-74 | 349 | 90 | 405 | 103 | 624 | 155 | 441 | 105 | 299 | 71 | 68 | 18 | 2,187 | 544 |  |
| 75-79 | 450 | 100 | 442 | 104 | 877 | 191 | 578 | 125 | 343 | 72 | 66 | 16 | 2,756 | 607 |  |
| 80-84 | 529 | 125 | 437 | 117 | 694 | 165 | 572 | 137 | 417 | 96 | 66 | 17 | 2,714 | 657 |  |
| 85+ | 639 | 105 | 490 | 93 | 711 | 108 | 623 | 103 | 421 | 75 | 80 | 15 | 2,964 | 497 |  |
| 50+ | 2,991 | 787 | 2,752 | 775 | 4,224 | 1,105 | 3,224 | 830 | 2,186 | 567 | 428 | 122 | 15,804 | 4,186 |  |

**2: Lifetime risk of fragility fractures**

**Background**

The number of elderly individuals is expected to increase across the European Union [10]. A larger elderly population will result in an increasing number of osteoporotic fractures, and thus greater need for improvements in treatment efficiency and effectiveness.

**Methods**

A micro-simulation model was used to estimate the life-time risk of fracture from the age of 50 years. This simulation required data on fracture incidence and mortality for each country. National data on hip incidence was available for all six countries [11-18], but information on other fractures was incomplete. Where relevant, the incidence of vertebral and other osteoporotic fractures was only available for Sweden except for UK where estimates for incidence of wrist fractures was available. The incidence of these types of fractures in the biggest 5 EU countries was imputed from the ratio of vertebral and other osteoporotic fractures to hip observed in Sweden [14]. The relative risk increase in mortality after fractures were derived from Swedish data and were applied on population mortality rates in the other countries [19-24].

**Results**

The remaining lifetime risk for women of sustaining a hip fracture at the age of 50 years vary between 9.8% in Spain to 22.8% in Sweden (Fig. A 2). Corresponding risk range for men is 6.1% (France) to 13.7% (Sweden). The highest remaining lifetime risk of a MOF is in Sweden with a 46.3 % risk for women and a 28.7 % risk for men.

The lifetime risk of hip fracture at age 50 is on the same level as the lifetime risk of a stroke for both women (20%) and men (14%) [25]. Lifetime risk of major osteoporotic fracture is comparable to that of cardiovascular disease (CVD), which affects 39% of women and 52% of men [26]. Table A 3 presents the number of estimated fractures by year and type.


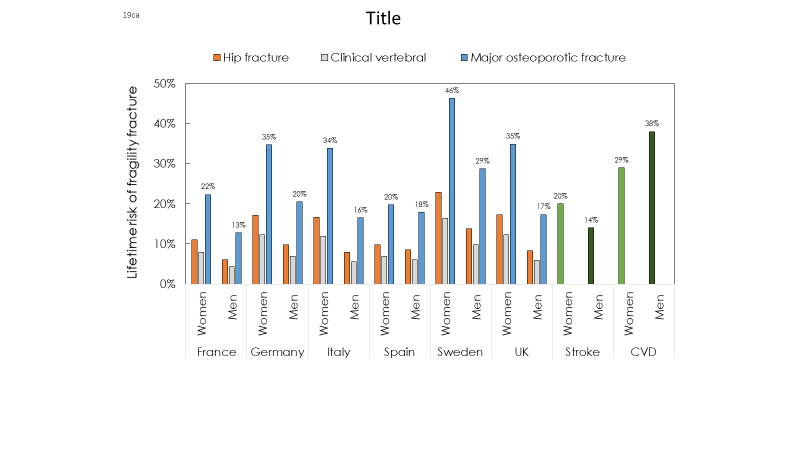


**Fig. A 2** Lifetime risk of fragility fracture from the age of 50, by country, and the equivalent risk for stroke and CVD. Source: National fracture incidences and own calculations

**Comments**

The lifetime risk of fracture varies between countries but is comparable to corresponding risk of CVD and Stroke.

**Supporting Data**

**Table A 3** Number of estimated fractures by year and type

|  | **Women** | |  |  |  | **Men** |  |  |  |
| --- | --- | --- | --- | --- | --- | --- | --- | --- | --- |
|  | 2017 | 2020 | 2025 | 2030 |  | 2017 | 2020 | 2025 | 2030 |
| **France** |  |  |  |  |  |  |  |  |  |
| Hip fractures | 58,020 | 61,081 | 65,269 | 72,325 |  | 19,208 | 20,689 | 23,254 | 26,444 |
| Vertebral fractures | 37,861 | 39,823 | 42,820 | 46,539 |  | 20,140 | 21,553 | 23,773 | 25,960 |
| MOFs | 130,317 | 137,063 | 146,887 | 160,952 |  | 63,077 | 67,442 | 74,345 | 82,260 |
| **Osteoporotic fractures** | **257,964** | **271,088** | **289,113** | **314,854** |  | **123,602** | **131,903** | **144,508** | **159,722** |
| **Germany** |  |  |  |  |  |  |  |  |  |
| Hip fractures | 106,190 | 111,480 | 118,952 | 125,421 |  | 39,682 | 43,526 | 48,854 | 53,231 |
| Vertebral fractures | 76,398 | 79,059 | 82,847 | 86,625 |  | 43,744 | 46,859 | 51,533 | 55,244 |
| MOFs | 250,982 | 261,616 | 275,902 | 289,252 |  | 135,432 | 146,299 | 160,884 | 171,860 |
| **Osteoporotic fractures** | **501,240** | **522,800** | **550,047** | **571,973** |  | **263,732** | **286,035** | **315,700** | **334,795** |
| **Italy** |  |  |  |  |  |  |  |  |  |
| Hip fractures | 82,060 | 86,007 | 91,891 | 98,539 |  | 28,875 | 31,493 | 35,602 | 39,473 |
| Vertebral fractures | 59,137 | 61,651 | 65,434 | 69,364 |  | 28,813 | 31,049 | 34,631 | 37,658 |
| MOFs | 194,132 | 202,938 | 216,078 | 230,120 |  | 90,767 | 98,085 | 109,371 | 119,582 |
| **Osteoporotic fractures** | **387,764** | **405,731** | **431,502** | **457,765** |  | **175,624** | **190,111** | **212,199** | **231,999** |
| **Spain** |  |  |  |  |  |  |  |  |  |
| Hip fractures | 47,294 | 50,447 | 54,642 | 59,589 |  | 23,394 | 25,275 | 28,431 | 31,883 |
| Vertebral fractures | 28,687 | 30,385 | 32,962 | 36,076 |  | 21,094 | 22,593 | 25,124 | 28,080 |
| MOFs | 101,897 | 108,349 | 117,529 | 128,314 |  | 68,573 | 73,499 | 82,057 | 91,798 |
| **Osteoporotic fractures** | **197,513** | **210,149** | **227,798** | **248,483** |  | **130,082** | **139,285** | **155,083** | **173,333** |
| **United Kingdom** |  |  |  |  |  |  |  |  |  |
| Hip fractures | 71,712 | 75,313 | 82,144 | 91,445 |  | 27,098 | 29,400 | 33,344 | 38,268 |
| Vertebral fractures | 52,977 | 55,789 | 59,808 | 65,026 |  | 27,817 | 29,975 | 33,001 | 36,768 |
| MOFs | 171,568 | 180,314 | 194,784 | 213,838 |  | 88,340 | 95,085 | 105,259 | 171,568 |
| **Osteoporotic fractures** | **348,472** | **365,768** | **393,955** | **429,948** |  | **170,504** | **183,335** | **202,363** | **226,824** |
| **Sweden** |  |  |  |  |  |  |  |  |  |
| Hip fractures | 16,456 | 17,020 | 18,680 | 20,856 |  | 6,481 | 7,011 | 8,196 | 9,344 |
| Vertebral fractures | 12,134 | 12,644 | 13,604 | 14,612 |  | 6,883 | 7,372 | 8,207 | 9,028 |
| MOFs | 39,236 | 40,709 | 44,201 | 48,287 |  | 21,658 | 23,163 | 26,152 | 29,041 |
| **Osteoporotic fractures** | **78,023** | **80,816** | **87,347** | **95,140** |  | **41,700** | **44,399** | **50,111** | **55,912** |

*MOFs = Major Osteoporotic Fracture (hip, spine, distal forearm and proximal humerus). Osteoporotic fractures include hip and vertebral fractures and other osteoporotic fractures.*

**3: Fracture projections**

**Background**

The number of elderly individuals is expected to increase across the European Union [10]. A larger elderly population will result in an increasing number of osteoporotic fractures, and thus greater need for improvements in treatment efficiency and effectiveness.

**Methods**

National data on fracture incidence by type and gender were combined with demographic projections over time to estimate the annual number of new fractures between 2017 and 2030. National data on hip incidence was available for all six countries [11-18] . Incidence of vertebral and other osteoporotic fractures were only available for Sweden with the exception of UK where an estimate for the incidence of wrist fractures was available. The incidence of these type of fractures in France, Germany, Italy and the UK was imputed from the ratio of vertebral and other osteoporotic fractures to hip observed in Sweden [14].

**Results**

The number of new fractures in EU6 in 2017 was estimated at 2.68 million. Of these, the majority (1 734 065 or 64.8%) were other osteoporotic fractures followed by hip (526 470 or 19.7%) and clinical vertebral fractures (415 685 or 15.5%).

Of all fractures, 50.7% were major osteoporotic fractures (MOFs) and 66.2% were sustained by women. The number of elderly individuals in all six countries is estimated to increase by 17.6 million (13.0%) between 2017 and 2030. The relative change of elderly individuals between 2017 and 2030 is highest in Spain (24.0%) and lowest in Germany (6.4%). By 2030, the total number of fractures in EU6 was estimated at 3.3 million, an increase of 23.3%. The largest percentage increase was observed in Spain (28.8%) and the lowest in Germany (18.5%). Fig. A 3 shows the estimated numbers of fractures by site in 2017 and 2030 and the percentage change, including estimates for the number of major osteoporotic fractures (MOFs). The figure also shows that Germany has the highest total of fragility fractures, due to its population size. Table A 3 presents the number of estimated fractures by year and type.

**Comments**

The number of elderly individuals is expected to increase in the EU6. Given current rates of incidence of hip, vertebral and other osteoporotic fractures, the burden of osteoporosis will continue to increase in all the countries studied. Across the six countries, the increase in incident fractures is greater than the increase in elderly individuals, indicating that the average age of elderly individuals will increase over time. Note that these projections take no account of possible changes in age- and sex-specific fracture rates with time.


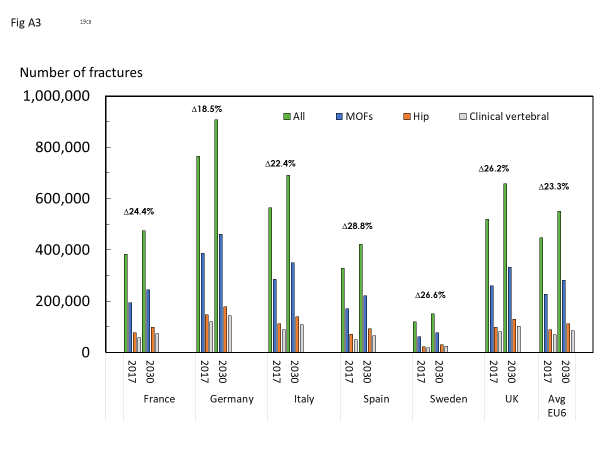


**Fig. A 3** Estimated number of fractures by site and country in 2017 and 2030 and percentage change

**4: Imminent fracture risk**

**Background**

Individuals who have already suffered a fragility fracture are at a greater risk for additional fractures both at the same bone site and at other sites. This additional risk of refracture, which is commonly termed imminent risk, is highest immediately after a fracture [28]. In this analysis, the imminent risk is illustrated by estimating the hazard ratio (HR) of a subsequent MOF and estimating the number of individuals exposed to the imminent risk of a recurrent fracture.

**Methods**

To show the number of patients exposed to an imminent risk of refracture in Sweden, patients age 50 years or older with an osteoporotic fracture of any type between 2006 and 2013 (Inclusion period) were identified from the Swedish National Patient Register (NPR). The date of first fracture during the inclusion period is set as the index date. Separate analysis was conducted for each fracture type, implying that one patient could have more than one index date. Fracture types were based on ICD-10 codes, which can be found in Table A 4. The 2-year risk of a subsequent fracture from index date was estimated using competing risk analysis. Death was considered the competing risk. Death dates were extracted from the Swedish Cause of Death Register. The 2-year risk of subsequent fracture was then estimated for each fracture type. Patients with a diagnosis of Paget’s disease or malignancies were excluded from the analysis. Vertebral fractures were limited to clinically vertebral fractures diagnosed within specialized inpatient or outpatient care.

In a separate analysis, the Hazard Ratio (HR) of a subsequent MOF in women with an index fracture at any site was estimated using a separate sample population who had not yet experienced a fragility fracture (fracture-naïve) as a reference case.

**Table A 4** Fracture codes by hip, vertebral, non-hip-non-vertebral, MOF and non-MOF

| Fracture site | ICD-10 | Hip | Vertebral | Non-hip-non-vertebral | MOF | Non-MOF | Comment |
| --- | --- | --- | --- | --- | --- | --- | --- |
| Neck | S12.x |  | S12.0x-S12.2x, S12.7x |  | S12.0x-S12.2x, S12.7x |  |  |
| Rib, sternum, thoracic spine | S22.x |  | S22.0x-S22.1x | S22.2x-S22.5x | S22.0x-S22.1x | S22.2x-S22.5x |  |
| Lumbar spine, pelvis | S32.x |  | S32.0x | S32.1x- S32.8x | S32.0x, S32.7x, S32.8x | S32.1x-S32.6x |  |
| Shoulder, upper arm | S42.x |  |  | S42.0x-S42.4x, S42.7x-S42.9x | S42.2x - S42.3x | S42.0x, S42.1x S42.4x-S42.9x |  |
| Forearm | S52.x |  |  | S52.x | S52.5x-S52.7x | S52.0x-S52.4x, S52.8x, S52.9x |  |
| Wrist, Hand | S62.0x, S62.8x |  |  |  |  |  | Not included in analyses |
| Femur (including hip) | S72.x | S72.0x-S72.2x |  | S72.3x-S72.9x | S72.ox- S72.2x | S72.3x-S72.9x |  |
| Lower leg (including ankle*) | S82.x |  |  | S82.1x S82.4x |  | S82.1x, S82.2x, S82.3x, S82.4x | Women only |
| Collapsed vertebra, not elsewhere specified | M48.5x |  | M48.5x |  | M48.5x |  |  |
| Osteoporosis with pathological fracture | M80.A, M80.B, M80.F, M80.J, M80.K | M80.F | M80.0A, M80.0J, M80.K | M80.B |  | M80.A, M80.B, M80.F, M80.J, |  |

**Results**

A total of 357,948 individuals with osteoporotic fractures were identified during the inclusion period. Most of these individuals (71%) were women. Available evidence suggest that these individuals face an increased risk of subsequent fractures within the following two years.

The separate analysis shows that the estimated HR is highest within the first six months after fracture. Furthermore, individuals with a previous fracture continue to have an excess risk of a subsequent fracture up until five years after fracture compared to a fracture-naïve population (Fig. A 4). Table A 5 shows the two-year cumulative subsequent fracture incidence by fracture type in Sweden, all ages.

**Comments**

This analysis finds a similar pattern as other studies in which the risk of a recurrent fracture is highest in the immediate aftermath of a fracture. This should be seen as an opportunity to improve fracture prevention care. Given the size of the osteoporotic population in Sweden, this can have a significant impact on the total burden of osteoporosis.


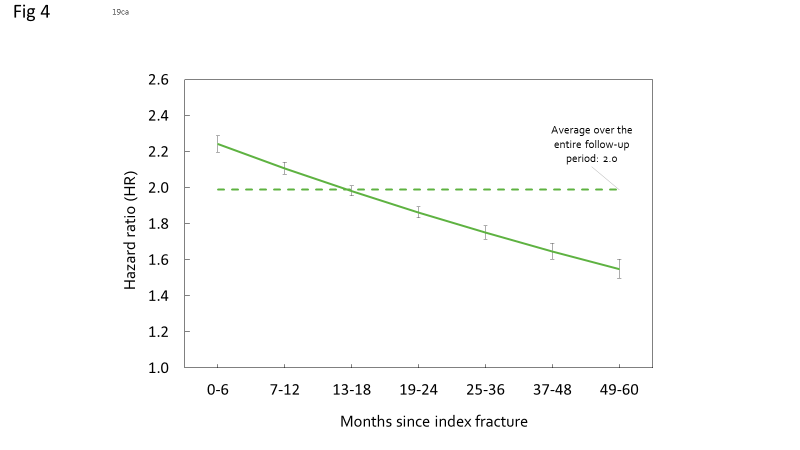


**Fig. A 4** Hazard ratio of a subsequent MOF in women with index fracture at any site versus non-fracture controls [28]

**Table A 5** Two-year cumulative subsequent fracture incidence by fracture type in Sweden, all ages

| **Fracture site by gender** | **No at risk at t=0** | **Cumulative incidence (%) at 1 year** | **Cumulative incidence (%) at 2 years** |
| --- | --- | --- | --- |
| **Women** |  |  |  |
| Hip fractures | 74,066 | 1.42 | 3.98 |
| Vertebral fractures | 23,794 | 1.46 | 3.27 |
| MOF | 195,533 | 4.57 | 8.28 |
| Osteoporotic fractures | 253,637 | 6.15 | 10.48 |
| **Men** |  |  |  |
| Hip fractures | 30,289 | 1.08 | 2.88 |
| Vertebral fractures | 11,187 | 1.39 | 2.45 |
| MOF | 64,255 | 3.38 | 5.96 |
| Osteoporotic fractures | 104,311 | 4.69 | 7.50 |

**5: Length of hospital stay**

**Background**

Fragility fractures incur increased costs to the health care sector and society. The costs to the health care sector are known to peak the year following fracture and the length of hospital stay is an important cost-component [29-31]. It is therefore of interest to study the length of hospital stay the following year after fracture.

**Methods**

Patients age 50 years or older with an incident fragility fracture that required inpatient care between 2010 and 2014 were identified from the Swedish National Patient Register (NPR). The total number of days of inpatient care during the following year after fracture was determined. The number of days admitted was categorized as “fracture-related” if the main diagnosis code for the admission was fracture related. The ICD-10 codes used to define an admission as fracture-related is listed in (see Table A 4). The analysis was conducted by fracture site. Patients with a diagnosis of Paget’s disease or malignancy anytime during the study period (2010-2015) were excluded from the analysis.

**Results**

Table A 6 shows the descriptive statistics of length of hospital stay for all fracture sites that required inpatient care in Sweden between 2010 and 2014. On average, a patient was hospitalized between 6- and 8-days following fracture. Table A 7 shows the descriptive statistics of the number of fracture related days hospitalized in the year following fracture, for all fractures sites that required inpatient care in Sweden between 2010 and 2014. This estimate is comprised of two components; the length-of-hospital stay in relation to the incident fracture (shown in Table A6) and readmissions. On average, a patient was hospitalized between 10 to 12 days due to fracture related causes including the fracture event (Table A 7). Hip fractures were associated with a higher number of days (12) compared to clinical vertebral fractures (10-11 days). Osteoporotic fractures required between 10 and 11 days of hospitalization. The number of days hospitalized was similar between the two genders.

Table A 8 shows the descriptive statistics of total days hospitalized following fracture. This analysis shows that on average, a patient with an incident fracture that requires inpatient care is in total hospitalized the following year between 15 to 20 days. Vertebral fractures are associated with a higher number of hospitalization days (20 days) compared to hip fractures (18-19 days) and osteoporotic fractures (17 days).

**Comments**

A substantial proportion (65-71%) of the fracture related hospitalization days the year after fracture was directly related to the length of stay in connection with the fracture. Moreover, of the total hospitalization days the year following fracture, a majority (53-69%) was fracture related.

**Supporting data**

**Table A 6** Number of days hospitalized in direct relation with incident fracture between 2010 and 2014 in Sweden

| **Fracture site by gender** | **N** | **Mean** | **SD** | **Q1** | **Median** | **Q3** |
| --- | --- | --- | --- | --- | --- | --- |
| **Women** |  |  |  |  |  |  |
| Hip fractures | 49,572 | 8.24 | 5.71 | 4 | 7 | 11 |
| Vertebral fractures | 10,367 | 7.69 | 5.91 | 3 | 7 | 10 |
| MOF | 75,333 | 7.87 | 5.84 | 4 | 7 | 11 |
| Osteoporotic fractures | 99,303 | 7.72 | 5.90 | 4 | 7 | 10 |
| **Men** |  |  |  |  |  |  |
| Hip fractures | 20,486 | 8.30 | 7.49 | 4 | 7 | 11 |
| Vertebral fractures | 5,090 | 6.70 | 6.23 | 2 | 5 | 9 |
| MOF | 29,464 | 7.73 | 7.16 | 3 | 6 | 10 |
| Osteoporotic fractures | 42,367 | 7.21 | 6.93 | 3 | 6 | 10 |

**Table A 7** Number of fracture-related days hospitalized the year following fracture between 2010 and 2014 in Sweden

|  | **N** | **Mean** | **SD** | **Q1** | **Median** | **Q3** |
| --- | --- | --- | --- | --- | --- | --- |
| **Women** |  |  |  |  |  |  |
| Hip fractures | 49,572 | 12.38 | 9.46 | 6 | 10 | 16 |
| Vertebral fractures | 10,367 | 11.35 | 10.15 | 5 | 9 | 15 |
| MOF | 75,333 | 11.56 | 9.49 | 5 | 9 | 15 |
| Osteoporotic fractures | 99,303 | 11.26 | 9.54 | 5 | 9 | 14 |
| **Men** |  |  |  |  |  |  |
| Hip fractures | 20,486 | 12.30 | 10.34 | 6 | 10 | 16 |
| Vertebral fractures | 5,090 | 10.35 | 10.98 | 3 | 7 | 13 |
| MOF | 29,464 | 11.49 | 10.42 | 5 | 9 | 15 |
| Osteoporotic fractures | 42,367 | 10.58 | 10.29 | 4 | 8 | 14 |

**Table A 8** Number of days hospitalized (all-causes) the year following fracture between 2010 and 2014 in Sweden

|  | **N** | **Mean** | **SD** | **Q1** | **Median** | **Q3** |
| --- | --- | --- | --- | --- | --- | --- |
| **Women** |  |  |  |  |  |  |
| Hip fractures | 49,572 | 17.84 | 17.02 | 7 | 13 | 22 |
| Vertebral fractures | 10,367 | 19.86 | 20.12 | 7 | 13 | 26 |
| MOF | 75,333 | 17.25 | 17.29 | 7 | 12 | 22 |
| Osteoporotic fractures | 99,303 | 16.84 | 17.24 | 6 | 12 | 21 |
| **Men** |  |  |  |  |  |  |
| Hip fractures | 20,486 | 19.31 | 19.55 | 7 | 13 | 24 |
| Vertebral fractures | 5,090 | 19.67 | 23.78 | 5 | 12 | 25 |
| MOF | 29,464 | 18.78 | 20.40 | 6 | 12 | 24 |
| Osteoporotic fractures | 42,367 | 17.38 | 19.89 | 5 | 11 | 22 |

**6: Fracture-related costs**

**Background**

Fragility fractures incur long-term consequences both in terms of costs and morbidity. However, the costs related to fracture is at its peak the period immediately following a fracture. The fracture costs in the first year following a fracture differ between fracture types, and to some extent reflect the severity of fracture. Hip fractures are the most severe fracture type, and almost always lead to hospitalization. The length of hospital stay is an important cost driver, and has also been shown to have implications on patient outcomes [30].

**Methods**

The mean length of stay following hip fracture, and fracture costs in the year after the fracture (reported in € 2017) (hip, vert and wrist) were retrieved from the scientific literature. All inputs are country specific. Age and gender specific data (where available) are shown in Table A 9. A literature search was undertaken utilizing PubMed as well as an ad-hoc google search.

**Results**

The mean length of stay following a hip fracture (Table A 10) was the highest in the UK (20.5 days), followed by Italy (19.0 days). Sweden had the shortest mean length of stay, 11.6 days, which was very similar to Spain where the corresponding was 11.8 days. As there were no large gender differences, the results are presented in total for both genders. The standard deviations were notably high in all countries except Germany, implying that there are considerable individual variations in length of hospital stay.

The fracture costs differed substantially over countries and fractures (Table A 11). Cost estimates for other fracture types than hip, vertebral and wrist fracture is very scarce and therefore not included. Hip fractures are the costliest fracture type in all countries, while wrist fractures are the least costly. Fracture costs are generally higher in Sweden and Germany, and the lowest in Spain. Table A 12 presents the sources for the average cost of fracture.

**Table A 9** Age and gender-specific costs (€ 2017)

|  |  | |  | **France** | | **Germany** | | **Italy** | | **Spain** | | **Sweden** | | **UK** | |
| --- | --- | --- | --- | --- | --- | --- | --- | --- | --- | --- | --- | --- | --- | --- | --- |
|  |  | | *Age* | *Male* | *Female* | *Male* | *Female* | *Male* | *Female* | *Male* | *Female* | *Male* | *Female* | *Male* | *Female* |
| **Hip** |  | | 50-64 | 12,856 | | 20,884 | | 21,307 | | 9,197 | 9,881 | 15,426 | 11,628 | 21,620 | 20,324 |
|  |  | | 65-74 |  |  |  |  |  |  |  |  | 14,070 | 13,553 |  |  |
|  |  | | 75-84 |  |  |  |  |  |  |  |  | 19,059 | 14,408 |  |  |
|  |  | | 85+ |  |  |  |  |  |  |  |  | 13,196 | 21,414 |  |  |
|  | | **Vertebral** | | 3,205 | | 11,824 | 11,803 | 4,713 | | 1,928 | | 14,474 | | 4,028 | |
|  | | **Wrist** | | 1,468 | | 1,275 | | 1,301 | | 533 | | 2,477 | | 2,568 | |

**Table A 10** Length of hospital stay following hip fracture, days, mean (SD)

| **Country** | ***Mean*** | ***SD*** | ***Source*** |
| --- | --- | --- | --- |
| **France** | 12.0 | 8.0 | [32] |
| **Germany** | 14.5 | 2.6 | [33] |
| **Italy** | 19.0 | 25.3 | [34] |
| **Spain** | 11.8 | 7.9 | [35] |
| **Sweden** | 11.6 | 8.7 | [30] |
| **UK** | 20.5 | 20.0 | [36] |

**Table A 11** Mean cost of fracture (€ 2017), during year after fracture occurrence

| Fracture | France | Germany | Italy | Spain | Sweden | UK |
| --- | --- | --- | --- | --- | --- | --- |
| **Hip** | 12,856 | 20,884 | 21,307 | 9,724 | 16,409 | 20,650 |
| **Vertebral** | 3,205 | 11,805 | 4,713 | 1,928 | 14,474 | 4,028 |
| **Wrist** | 1,468 | 1,275 | 1,301 | 533 | 2,477 | 2,568 |

**Table A 12** Sources for the average cost of fracture

|  | France | Germany | Italy | Spain | Sweden | UK |
| --- | --- | --- | --- | --- | --- | --- |
| **Hip** | Adapted from, [37]; original sources: [38, 39] | [40] | [41] | [35] | [42] | [36] |
| **Vertebral** |  | [43] |  | [44] |  | [45] |
| **Wrist** |  | [40] |  |  |  |  |

**7: Annual cost of fractures**

**Background**

The societal burden of a disease can be divided into the economic burden and the health burden. The economic burden comprises the monetary costs to society related to the presence of the disease. The cost of illness can play an important role in understanding the implications of a disease and aid decisions concerning allocation of societal resources.

**Methods**

Fracture related costs, risks and mortality with fracture in each country were retrieved from the literature [37, 42, 45-49]. The projected annual costs related to osteoporotic fractures between years 2017-2030 were estimated accounting for both the short-term and long-term costs (due to fractures that arose in previous years) of fractures as well as the costs of residing in nursing homes. A model previously used to estimate the burden of osteoporosis in Sweden [50] was adapted to the countries included in the present study [50].The costs incurred by patients with a history of more than one fracture (subsequent fractures) was calculated from the differences between the risk of fracture (including those with a prior fracture) and risk of first fracture [14].

**Results**

The total monetary cost was €37.5 billion in 2017. Hip fracture represented 56% of the total fracture related costs across EU6 whilst other osteoporotic fractures and vertebral fractures accounted for 35 and 8%, respectively. Whereas hip fractures accounted for 20% of all fragility fractures, they accounted for 56 % of costs (Fig. A 5).


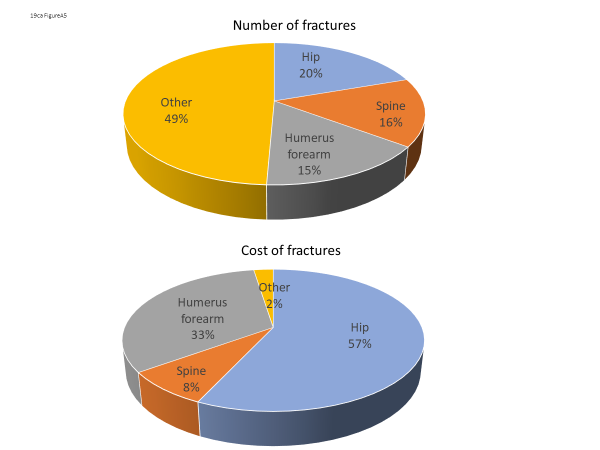


**Fig. A 5** Fracture related costs (panel A, % and billion €) and number of fractures (panel B, n and %) in 2017 by fracture type, EU6.

Fig. A 6 shows the cost incurred by patients with a history of more than one fracture and the cost incurred by patients with only one fracture in 2017 in all six countries by fracture type. Of the total fracture related cost, on average 15.7% (13.1 to 8.5% depending on fracture type) is accounted for patients with more than one fracture.


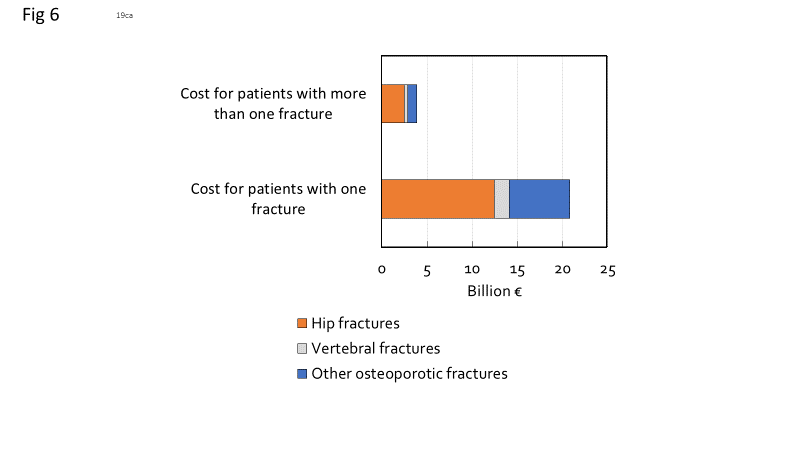


**Fig. A 6** Total fracture cost by fracture history in 2017, EU6


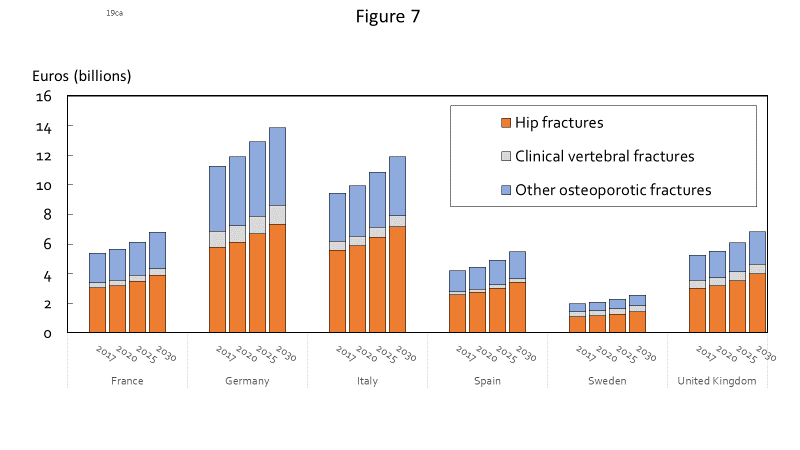
The fracture related costs in EU6 are projected to increase from €37.5 billion in year 2017 to €47.4 billion in year 2030, an increase of 26.6% (Fig. A 7). Mainly due to different population sizes, the burden is the highest in Germany and lowest in Sweden. On a per capita basis, Sweden ranked first and UK last. However, when fracture costs are considered in relation to health care spending, Italy could be interpreted as the country with the highest economic fracture burden (Fig. A 8) whereas France and the UK appear to have the lowest fracture burden. Table A 13 presents the fracture related costs in 2017. The fracture-related costs in terms of health care spending and population are presented in Table A 14. Table A 15 presents the annual fracture-related cost projections.

**Fig. A 7** Estimated annual fracture related costs (billion euro) by type and country in 2017 and 2030 and percentage change


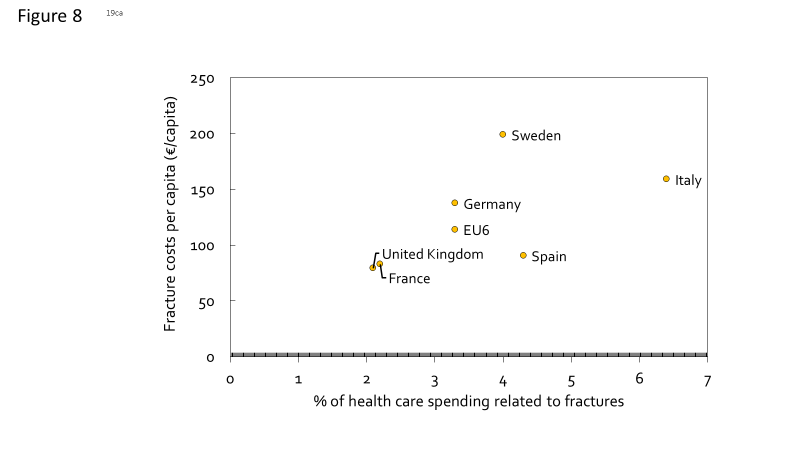


**Fig. A 8** Fracture cost per capita and health care spending related to fractures

**Comments**

The number of elderly individuals is expected to increase in the EU6. Given current rates of incidence of hip vertebral and other osteoporotic fractures, the monetary burden of osteoporosis will increase in all countries studied. The relative change in monetary burden between 2017 and 2030 is similar to the burden in terms of QALYs.

**Supporting data**

**Table A 13** Fracture related costs in 2017 (million euro)

| Country | Incident fractures | Prevalent fractures | Institutional care | Total |
| --- | --- | --- | --- | --- |
| France | 3,748 | 219 | 1,404 | 5,371 |
| Germany | 8,176 | 414 | 2,680 | 11,270 |
| Italy | 5,951 | 299 | 3,179 | 9,429 |
| Spain | 2,150 | 137 | 1,915 | 4,202 |
| United Kingdom | 2,955 | 372 | 1,919 | 5,246 |
| Sweden | 1,199 | 81 | 690 | 1,970 |

**Table A 14** Fracture related costs in terms of health care spending and population

| Country | *% of health care spending on fractures* | *Burden per capita (€)* |
| --- | --- | --- |
| **France** | 2.2% | 83 |
| **Germany** | 3.3% | 137 |
| **Italy** | 6.4% | 159 |
| **Spain** | 4.3% | 91 |
| **United Kingdom** | 2.1% | 79 |
| **Sweden** | 4.0% | 199 |

*Estimates from 2015 [51]

**Table A 15** Fracture related costs by year, million euro

|  | 2017 | 2020 | 2025 | 2030 |
| --- | --- | --- | --- | --- |
| **France** |  |  |  |  |
| Incident costs | 3,748 | 3,965 | 4,259 | 4,675 |
| Prevalent costs | 219 | 228 | 251 | 281 |
| Institutional care costs | 1,404 | 1,467 | 1,620 | 1,832 |
| **Total** | **5,371** | **5,660** | **6,130** | **6,788** |
| **Germany** |  |  |  |  |
| Incident costs | 8,176 | 8,672 | 9,340 | 9,847 |
| Prevalent costs | 414 | 430 | 468 | 519 |
| Institutional care costs | 2,680 | 2,802 | 3,101 | 3,519 |
| **Total** | **11,270** | **11,904** | **12,909** | **13,885** |
| **Italy** |  |  |  |  |
| Incident costs | 5,951 | 6,309 | 6,840 | 7,367 |
| Prevalent costs | 299 | 311 | 339 | 377 |
| Institutional care costs | 3,179 | 3,322 | 3,671 | 4,158 |
| **Total** | **9,429** | **9,942** | **10,850** | **11,902** |
| **Spain** |  |  |  |  |
| Incident costs | 2,150 | 2,296 | 2,518 | 2,776 |
| Prevalent costs | 137 | 144 | 158 | 179 |
| Institutional care costs | 1,915 | 2,004 | 2,224 | 2,532 |
| **Total** | **4,202** | **4,444** | **4,900** | **5,487** |
| **United Kingdom** | |  |  |  |
| Incident costs | 2,955 | 3,140 | 3,437 | 3,822 |
| Prevalent costs | 372 | 388 | 427 | 481 |
| Institutional care costs | 1,919 | 2,007 | 2,225 | 2,529 |
| **Total** | **5,246** | **5,535** | **6,088** | **6,832** |
| **Sweden** |  |  |  |  |
| Incident costs | 1,199 | 1,255 | 1,385 | 1,538 |
| Prevalent costs | 81 | 85 | 93 | 104 |
| Institutional care costs | 690 | 721 | 799 | 907 |
| **Total** | **1,970** | **2,061** | **2,276** | **2,549** |

**8: Quality-Adjusted Life-Years**

**Background**

The societal burden of a disease can be differentiated into the economic burden and the health burden. The economic burden concerns the actual costs inflicted on society related to the presence of the disease. The health burden concerns the impact that the disease has on the patient in terms of quality of life (QoL) and reduced survival. The most common approach to estimate the health burden is to use the Quality-Adjusted Life-Years (QALYs) [52].

**Methods**

Quality of life values for the general normal population was combined with fracture related disutility multipliers to estimate the total QALY loss in each country [53, 54]. All QoL data used in the calculations was based the EQ-5D questionnaire [54]. Increased risk of mortality after fractures was derived from the literature [55]. The health burden, e.g. QALYs lost, associated with fragility fractures was estimated from year 2017 up to year 2030 based on projected demographic changes during this period.

**Results**

The total health burden in 2017 due to fragility fractures in the six countries is estimated at 1.02 million QALYs. 66% of the QALY loss is related to fractures occurring in women. The total health burden is expected to increase by 26% by 2030 (Fig. A 9). The QALY loss in absolute numbers was highest in Germany due to the size of the population combined with comparatively high risk of fractures. The lowest QALY loss was observed in Sweden due to the small population size compared to the other countries. However, on a per capita basis, Sweden was associated with the largest burden (4.22 lost QALYs per 1,000 population) and France the lowest (2.11 lost QALYs per 1,000 capita). The differences are driven, in large part, by the differences in the risk of fractures between the countries (Fig. A 10) and the age-distribution among elderly individuals. Table A 16 -Table A 20 present the supporting data for the burden of fragility fractures.


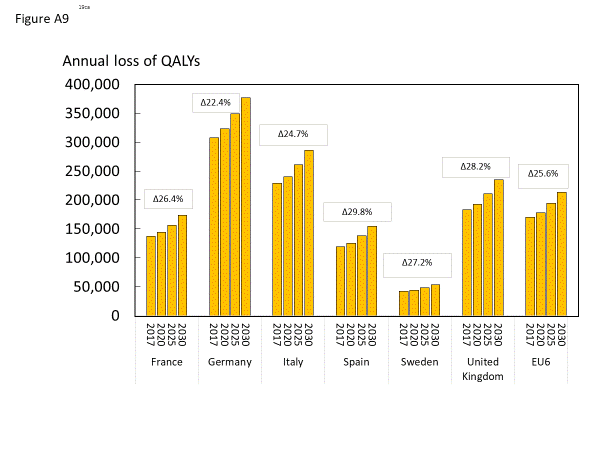


**Fig. A 9** Total annual loss of QALYs by country in 2017 and 2030 and percentage change


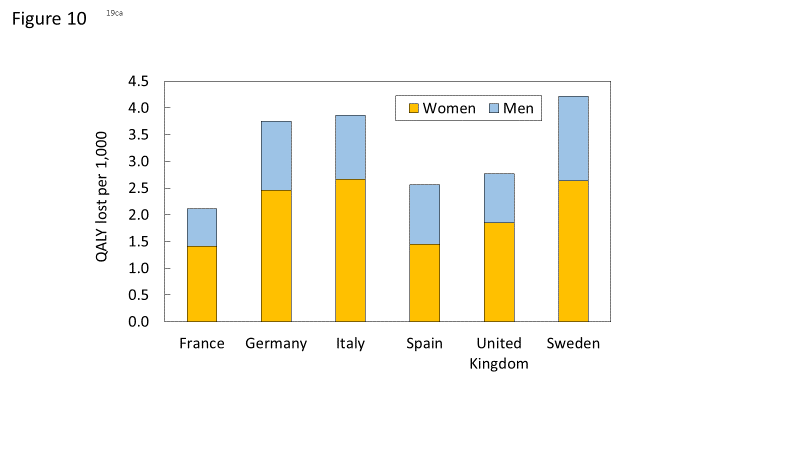


**Fig. A 10** QALYs lost due to fragility fractures per 1,000 in 2017 by gender and country

**Comments**

Because of the increasingly elderly population the health burden (in terms of lost QALYs) related to fractures will continue to rise over the next decade. Even though there are some variation between countries due to demographic differences this pattern is apparent in all countries.

**Supporting data**

**Table A 16** Estimated loss of QALYs per 1,000 population in 2017, both sexes

|  | **France** | **Germany** | **Italy** | **Spain** | **United Kingdom** | **Sweden** |
| --- | --- | --- | --- | --- | --- | --- |
| **QALY loss due to morbidity** |  |  |  |  |  |  |
| *Incident hip fractures* | 0.297 | 0.500 | 0.529 | 0.401 | 0.366 | 0.562 |
| *Incident vertebral fractures* | 0.180 | 0.329 | 0.336 | 0.229 | 0.239 | 0.380 |
| *Incident MOFs* | 0.488 | 0.880 | 0.885 | 0.605 | 0.645 | 0.989 |
| *Prevalent hip fractures* | 0.408 | 0.664 | 0.731 | 0.471 | 0.477 | 0.745 |
| *Prevalent vertebral fractures* | 0.500 | 0.873 | 0.934 | 0.564 | 0.608 | 1.023 |
| *Prevalent MOFs* | 0.119 | 0.224 | 0.217 | 0.132 | 0.250 | 0.242 |
| **Total QALY loss due to morbidity** | **1.993** | **3.471** | **3.632** | **2.401** | **2.585** | **3.941** |
| **QALY loss due to mortality** |  |  |  |  |  |  |
| *Mortality hip fractures* | 0.051 | 0.114 | 0.098 | 0.071 | 0.077 | 0.113 |
| *Mortality vertebral fractures* | 0.067 | 0.162 | 0.126 | 0.083 | 0.104 | 0.160 |
| *Mortality MOFs* | 0.003 | 0.006 | 0.006 | 0.004 | 0.004 | 0.006 |
| **Total QALY loss due to mortality** | **0.121** | **0.282** | **0.230** | **0.158** | **0.186** | **0.279** |
| **Total QALY loss due to**  **osteoporotic fractures** | **2.114** | **3.753** | **3.862** | **2.560** | **2.770** | **4.220** |

**Table A 17** Estimated total QALY loss of fractures in 2017, female population

|  | **France** | **Germany** | **Italy** | **Spain** | **United Kingdom** | **Sweden** |
| --- | --- | --- | --- | --- | --- | --- |
| **QALY loss due to morbidity** |  |  |  |  |  |  |
| *Incident hip fractures* | 14,225 | 29,425 | 22,871 | 11,819 | 17,399 | 3,844 |
| *Incident vertebral fractures* | 7,476 | 16,975 | 13,194 | 5,798 | 10,360 | 2,316 |
| *Incident MOFs* | 20,199 | 45,071 | 34,758 | 15,422 | 27,853 | 5,958 |
| *Prevalent hip fractures* | 18,996 | 38,238 | 31,667 | 13,340 | 22,217 | 4,724 |
| *Prevalent vertebral fractures* | 21,935 | 47,545 | 39,066 | 14,670 | 27,519 | 6,283 |
| *Prevalent MOFs* | 4,853 | 11,303 | 8,668 | 3,139 | 9,643 | 1,422 |
| **Total QALY loss due to morbidity** | **87,683** | **188,557** | **150,223** | **64,188** | **114,991** | **24,546** |
| **QALY loss due to mortality** |  |  |  |  |  |  |
| *Mortality hip fractures* | 2,020 | 5,701 | 3,669 | 1,681 | 3,264 | 666 |
| *Mortality vertebral fractures* | 2,031 | 6,629 | 4,171 | 1,401 | 3,899 | 805 |
| *Mortality MOFs* | 169 | 411 | 286 | 147 | 228 | 50 |
| **Total QALY loss due to mortality** | **4,221** | **12,741** | **8,125** | **3,229** | **7,391** | **1,521** |
| **Total QALY loss due to**  **osteoporotic fractures** | **91,904** | **201,298** | **158,349** | **67,418** | **122,382** | **26,067** |

**Table A 18** Estimated burden of fractures in 2017, male population

|  | **France** | **Germany** | **Italy** | **Spain** | **United Kingdom** | **Sweden** |
| --- | --- | --- | --- | --- | --- | --- |
| **QALY loss due to morbidity** |  |  |  |  |  |  |
| *Incident hip fractures* | 5,064 | 11,615 | 8,535 | 6,776 | 6,804 | 1,722 |
| *Incident vertebral fractures* | 4,237 | 10,056 | 6,735 | 4,823 | 5,480 | 1,445 |
| *Incident MOFs* | 11,492 | 27,113 | 17,797 | 12,661 | 14,803 | 3,842 |
| *Prevalent hip fractures* | 7,529 | 16,273 | 11,693 | 8,537 | 9,352 | 2,659 |
| *Prevalent vertebral fractures* | 10,570 | 24,076 | 16,353 | 11,513 | 12,724 | 3,851 |
| *Prevalent MOFs* | 2,887 | 7,067 | 4,221 | 2,970 | 6,877 | 975 |
| **Total QALY loss due to morbidity** | **41,778** | **96,199** | **65,335** | **47,280** | **56,040** | **14,493** |
| **QALY loss due to mortality** |  |  |  |  |  |  |
| *Mortality hip fractures* | 1,301 | 3,665 | 2,141 | 1,633 | 1,855 | 453 |
| *Mortality vertebral fractures* | 2,324 | 6,656 | 3,319 | 2,452 | 2,988 | 778 |
| *Mortality MOFs* | 39 | 91 | 64 | 42 | 46 | 11 |
| **Total QALY loss due to mortality** | **3,663** | **10,412** | **5,524** | **4,127** | **4,889** | **1,242** |
| **Total QALY loss due to**  **osteoporotic fractures** | **45,442** | **106,611** | **70,859** | **51,408** | **60,929** | **15,735** |

**Table A 19** Estimated burden of fractures in 2030, female population

|  | **France** | **Germany** | **Italy** | **Spain** | **United Kingdom** | **Sweden** |
| --- | --- | --- | --- | --- | --- | --- |
| **QALY loss due to morbidity** |  |  |  |  |  |  |
| *Incident hip fractures* | 17,692 | 34,711 | 27,425 | 14,899 | 22,121 | 4,833 |
| *Incident vertebral fractures* | 9,134 | 19,206 | 15,447 | 7,300 | 12,651 | 2,757 |
| *Incident MOFs* | 24,255 | 50,674 | 40,731 | 19,390 | 33,846 | 7,116 |
| *Prevalent hip fractures* | 24,056 | 47,584 | 39,659 | 17,273 | 28,338 | 5,903 |
| *Prevalent vertebral fractures* | 27,641 | 57,881 | 48,115 | 18,771 | 34,801 | 7,794 |
| *Prevalent MOFs* | 6,116 | 13,744 | 10,664 | 4,007 | 12,182 | 1,773 |
| **Total QALY loss due to morbidity** | **108,894** | **223,798** | **182,041** | **81,640** | **143,938** | **30,177** |
| **QALY loss due to mortality** |  |  |  |  |  |  |
| *Mortality hip fractures* | 2,549 | 6,762 | 4,442 | 2,117 | 4,201 | 855 |
| *Mortality vertebral fractures* | 2,550 | 7,671 | 4,950 | 1,738 | 4,899 | 1,003 |
| *Mortality MOFs* | 211 | 508 | 359 | 191 | 290 | 63 |
| **Total QALY loss due to mortality** | **5,310** | **14,941** | **9,750** | **4,046** | **9,391** | **1,921** |
| **Total QALY loss due to**  **osteoporotic fractures** | **114,204** | **238,739** | **191,791** | **85,686** | **153,329** | **32,098** |

**Table A 20** Estimated burden of fractures in 2030, male population

|  | **France** | **Germany** | **Italy** | **Spain** | **United Kingdom** | **Sweden** |
| --- | --- | --- | --- | --- | --- | --- |
| **QALY loss due to morbidity** |  |  |  |  |  |  |
| *Incident hip fractures* | 6,908 | 15,525 | 11,623 | 9,234 | 9,574 | 2,453 |
| *Incident vertebral fractures* | 5,408 | 12,674 | 8,777 | 6,422 | 7,225 | 1,875 |
| *Incident MOFs* | 14,476 | 33,884 | 23,263 | 16,767 | 19,361 | 5,038 |
| *Prevalent hip fractures* | 10,170 | 22,280 | 15,989 | 11,472 | 12,883 | 3,652 |
| *Prevalent vertebral fractures* | 13,884 | 31,559 | 21,584 | 15,287 | 17,049 | 5,076 |
| *Prevalent MOFs* | 3,810 | 9,217 | 5,556 | 3,929 | 9,105 | 1,297 |
| **Total QALY loss due to morbidity** | **54,656** | **125,139** | **86,792** | **63,112** | **75,197** | **19,392** |
| **QALY loss due to mortality** |  |  |  |  |  |  |
| *Mortality hip fractures* | 1,774 | 4,787 | 2,875 | 2,193 | 2,580 | 649 |
| *Mortality vertebral fractures* | 2,947 | 8,199 | 4,167 | 3,221 | 3,869 | 1,008 |
| *Mortality MOFs* | 55 | 141 | 98 | 58 | 68 | 17 |
| **Total QALY loss due to mortality** | **4,776** | **13,127** | **7,140** | **5,471** | **6,517** | **1,673** |
| **Total QALY loss due to**  **osteoporotic fractures** | **59,432** | **138,266** | **93,932** | **68,583** | **81,714** | **21,065** |

**9:** **Disability-Adjusted Life-Years (DALYs)**

**Background**

The DALY (or Disability Adjusted Life Year) is a method of measuring the burden of a disease, which allows for comparison across diseases. A single DALY can be thought of as one lost year of “healthy life” and summing the DALYs across an entire population provides the gap between the current health status of a population and an ideal disease-free population, i.e. the burden [56]. Including this measure of burden allows for both comparison of the burden between populations, and comparison with other diseases.

**Methods**

Calculation of DALYs for fragility fractures follows the current method used and provided by the WHO. DALYs are the sum of years of life lost (YLL) and the years lost due to disability (YLD) [56]. YLL was calculated by multiplying the total incidence by age and sex for each fracture by the change in life expectancy due to that fracture. YLD was calculated by multiplying the number of fractures with the remaining life years for a person with an osteoporotic fracture at a given age and gender, and a disability weight for living with that osteoporotic fracture.

Data were assembled for the largest 5 EU countries as well as Sweden (total population: 128,699,382), to calculate the per capita and total DALYs in men and women from the age of 50 to 100 years, for hip fractures, vertebral fractures and a weighted average of other fractures associated with osteoporosis (rib, sternum, pelvis, shoulder, forearm, wrist, and other femoral fractures [57, 58]), which are referred to as Non-hip and non-vertebral fractures (NHNV). Estimates for fracture incidence rates were from Svedbom et al 2013 [6] and relative fracture mortality rates from Jönsson et al 2011 [47]. Population by age and sex, as well as normal mortality rates, were obtained through the statistics bureaus of each country [59-64], and WHO standardized fracture disutility weights were used in the calculation [65].

Based on Kanis et al 2003 and Kanis 2004 [66, 67], it was assumed that 30% of the excess mortality associated with hip and vertebral fractures can be causally related to the fracture itself. This proportion was also applied to the NHNV fractures [58]. In accord with current WHO standards, age weighting was not used in the estimation [68].

**Results**

The total DALYs estimated in year 2016 for people in all six countries combined from the ages of 50 to 100 years shows that more than 2.6 million life years are lost due to fragility fracture. The DALY totals for each country vary greatly due to differences in population (Fig. A 11). The average DALY loss per 1000 individuals was estimated to be 21 DALYs, with Sweden showing the highest rate (32 DALYs) and Spain showing the lowest (12 DALYs), as seen in Fig. A 12. Average YLD’s per 1000 people (15.1) far exceeded the YLL’s per 1000 (5.5), indicating that living with a disability due to fracture drives DALY loss in osteoporosis. DALYs rate varied by country, age, gender and fracture type. In all countries, women had higher DALY burden than men, with averages of 23.8 and 17.0 DALYs, respectively. The fracture category with the highest total DALY loss was “NHNV fractures” (Fig. A 13). This dominance can be explained by the combination of a high incidence at early ages, and the large number of years spent with disability due the less life-threatening nature of NHNV fractures. This is shown in Fig. A 14 and Fig. A 15, where the age distribution of YLLs and YLDs by gender for two fracture types are shown.

For hip fractures in women, the YLLs peaked at the age of 77 years, whilst the YLDs peaked at age 81 years, reflecting that most hip fractures occur around 77 years. For “NHNV fractures” in the male population, the peak is early, with very low YLLs, indicating that prevalence of NHNV fractures is fairly high. Table A 21 and Table A 22 present the supporting data for DALYs and DALYs per 1000.


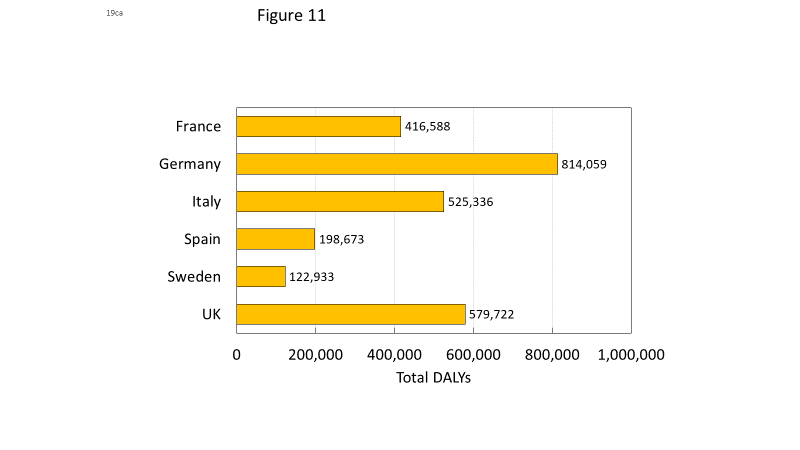


**Fig. A 11** Total DALYs by Country

**
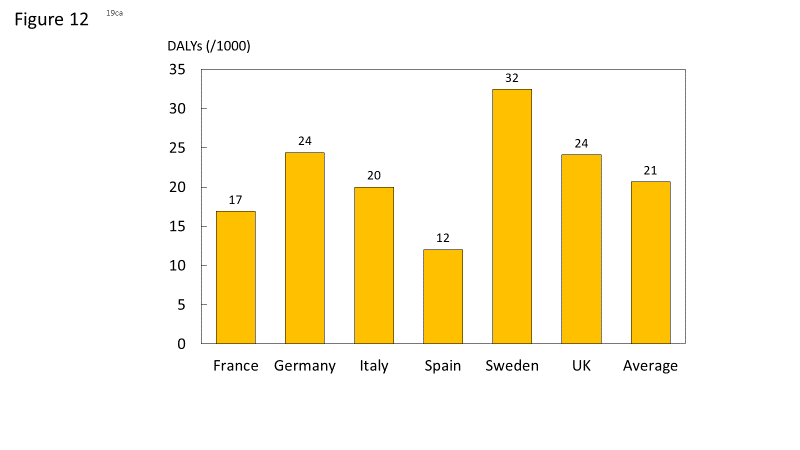
**

**Fig. A 12** DALYs per 1000 individuals by country

**
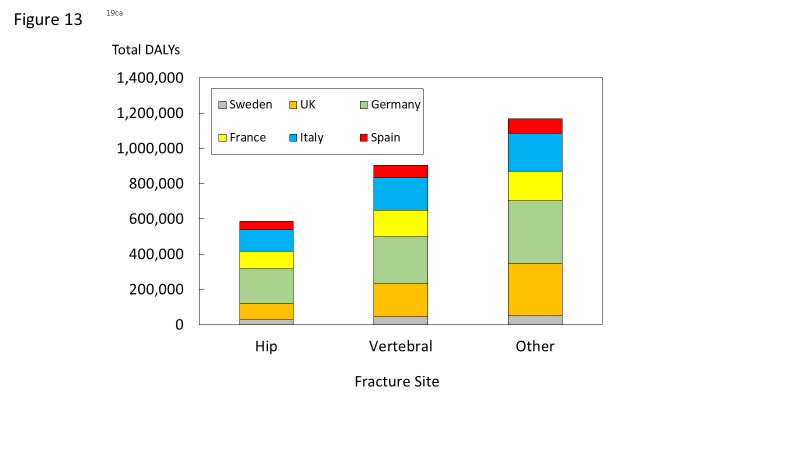
**

**Fig. A 13** Total DALY Distribution by fracture site

**
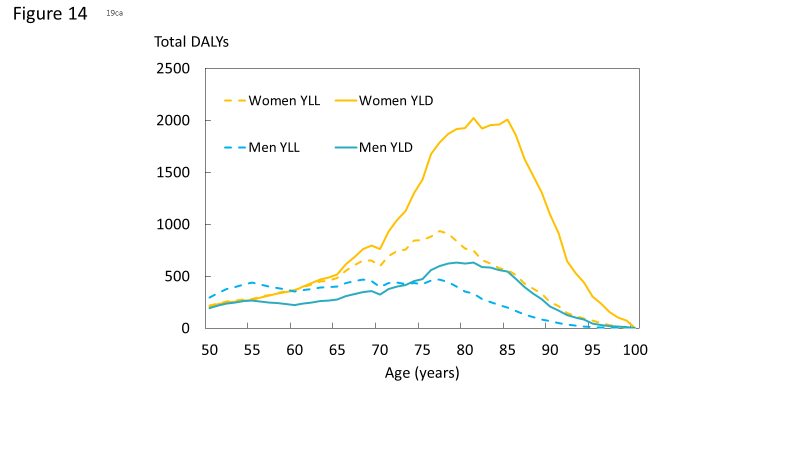
**

**Fig. A 14** Total DALYs by age for hip fractures


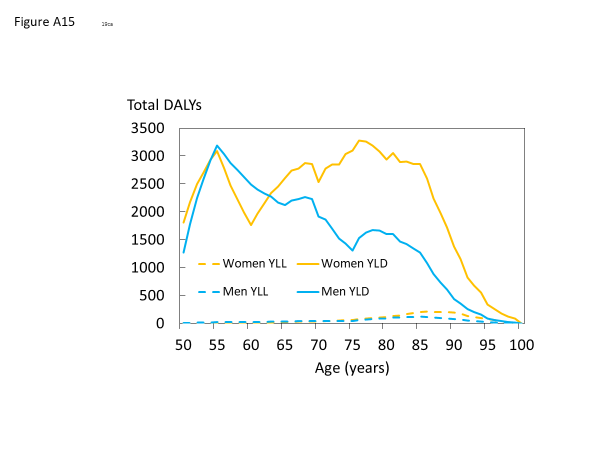


**Fig. A 15** Total DALYs by age for non-hip non-vertebral fractures

**Comments**

From a national perspective, the DALY loss rate can be an important measure for motivating policy decisions and the prioritization of funds towards osteoporosis treatment. From an international perspective, the high values suggest a need for better treatment policy and practice. The assumption that 30% of deaths are causally linked to all fracture types in this study comes with the caveat that NHNV fractures, and vertebral fractures to a lesser degree, have a much lower relative mortality, which compensates for the constant percentage applied to causally related deaths.

**Supporting data**

**Table A 21** YLLs and YLDs lost, by fracture type, country and gender

|  | **Total DALYS** | | | | | | | | | | | |  |
| --- | --- | --- | --- | --- | --- | --- | --- | --- | --- | --- | --- | --- | --- |
|  | Hip Fractures | | | | Vert Fractures | | | | NHNV Fractures | | | |  |
|  | *YLL* | | *YLD* | | *YLL* | | *YLD* | | *YLL* | | *YLD* | |  |
|  | *Women* | *Men* | *Women* | *Men* | *Women* | *Men* | *Women* | *Men* | *Women* | *Men* | *Women* | *Men* | *Totals* |
| Sweden | 6,343 | 4,265 | 11,907 | 5,046 | 12,326 | 10,180 | 15,183 | 8,013 | 796 | 602 | 26,342 | 21,931 | 122933 |
| UK | 20,803 | 18,710 | 34,656 | 19,941 | 52,142 | 46,287 | 56,713 | 31,250 | 4,989 | 3,757 | 159,474 | 131,002 | 579722 |
| Germany | 47,632 | 29,744 | 89,173 | 28,077 | 70,777 | 68,765 | 84,973 | 41,353 | 6,449 | 3,957 | 209,247 | 133,910 | 814059 |
| France | 23,824 | 13,561 | 45,969 | 15,590 | 43,716 | 32,867 | 49,858 | 22,903 | 3,637 | 2,178 | 93,449 | 69,035 | 416588 |
| Italy | 26,996 | 15,375 | 61,660 | 19,814 | 49,010 | 34,823 | 74,017 | 28,901 | 4,035 | 2,681 | 127,618 | 80,405 | 525336 |
| Spain | 9,678 | 6,233 | 24,994 | 7,439 | 16,296 | 13,781 | 28,271 | 10,412 | 1,706 | 1,127 | 48,038 | 30,698 | 198673 |
| **Total** | 135,277 | 87,888 | 268,358 | 95,906 | 244,267 | 206,703 | 309,015 | 142,831 | 21,614 | 14,302 | 664,169 | 466,981 | 2,657,311 |

**Table A 22** YLLs and YLDs per 1000 people, by fracture type, country and gender

|  | **DALYs per 1000** | | | | | | | | | | | | | |  |
| --- | --- | --- | --- | --- | --- | --- | --- | --- | --- | --- | --- | --- | --- | --- | --- |
|  | *Hip Fractures* | | | | *Vert Fractures* | | | | | *NHNV Fractures* | | | | |  |
|  | *YLL* | | *YLD* | | *YLL* | | *YLD* | | | *YLL* | | | *YLD* | |  |
|  | Women | Men | Women | Men | Women | Men | Women | Men | Women | | Men | Women | | Men | Average |
| Sweden | 3.23277 | 2.33641 | 6.0684 | 2.76432 | 6.28184 | 5.57727 | 7.73806 | 4.38979 | 0.40592 | | 0.32963 | 13.42551 | | 12.01514 | 32.45845 |
| UK | 1.64171 | 1.64467 | 2.73491 | 1.75287 | 4.11485 | 4.06876 | 4.4756 | 2.74697 | 0.39375 | | 0.33022 | 12.58515 | | 11.51552 | 24.10719 |
| Germany | 2.66006 | 1.91983 | 4.97992 | 1.81227 | 3.95261 | 4.43852 | 4.7454 | 2.66914 | 0.36017 | | 0.25543 | 11.68555 | | 8.64335 | 24.3735 |
| France | 1.77705 | 1.2037 | 3.42891 | 1.3838 | 3.26085 | 2.91743 | 3.71898 | 2.03299 | 0.27132 | | 0.19334 | 6.97052 | | 6.12776 | 16.88489 |
| Italy | 1.89768 | 1.27527 | 4.33432 | 1.64341 | 3.44514 | 2.88833 | 5.20297 | 2.39716 | 0.28362 | | 0.22239 | 8.9708 | | 6.6691 | 19.98818 |
| Spain | 1.08678 | 0.81962 | 2.80667 | 0.97817 | 1.82995 | 1.81198 | 3.17465 | 1.36899 | 0.19161 | | 0.14816 | 5.39446 | | 4.03648 | 12.03322 |
| **Average** | 1.95833 | 1.47409 | 3.88488 | 1.60858 | 3.53613 | 3.46691 | 4.47345 | 2.39562 | 0.31289 | | 0.23988 | 9.61482 | | 7.8324 | 20.64743 |

**10:** **DALY Comparison across diseases**

**Background**

Disability Adjusted Life Years (DALYs) are a measure of disease burden which allows for comparison between diseases. Comparing the burden of osteoporosis (fragility fractures) to the burden of other diseases gives policy makers the knowledge to allocate resources appropriately with regards to treatment and prevention.

**Methods**

The osteoporosis DALY estimates from section 1p were combined with corresponding 2016 estimates from the Institute for Health Metrics and Evaluation (IHME) for 16 selected diseases by country and sex [69].

**Results**

The osteoporosis estimates place the burden between that of lung cancer and chronic obstructive pulmonary disease (COPD), which are two of the most burdensome diseases in these European countries (Fig. A 16). Germany makes up the greatest portion of DALY burden in each of the disease categories, followed by Italy, though total DALYs correlate strongly with population size.

Converting the total DALYs by disease into DALYs per 1,000 individuals is shown in Fig. A 17. The DALYs lost per 1 000 individuals due to fragility fractures were estimated at 20.7 years, with Sweden showing the highest rate (32 DALYS) and Spain showing the lowest (12 DALYS). In this case, osteoporosis is estimated to fall between lung cancer and COPD. The DALY burden varies between countries due to differences in age distributions, risk of fracture and death. In Sweden, for example, the DALY burden of fractures is higher than that for dementia whereas in Spain the burden related to dementia, lung cancer and COPD surpasses that for fractures. Table A 23 and Table A 24 present the supporting data for DALY comparison.

**
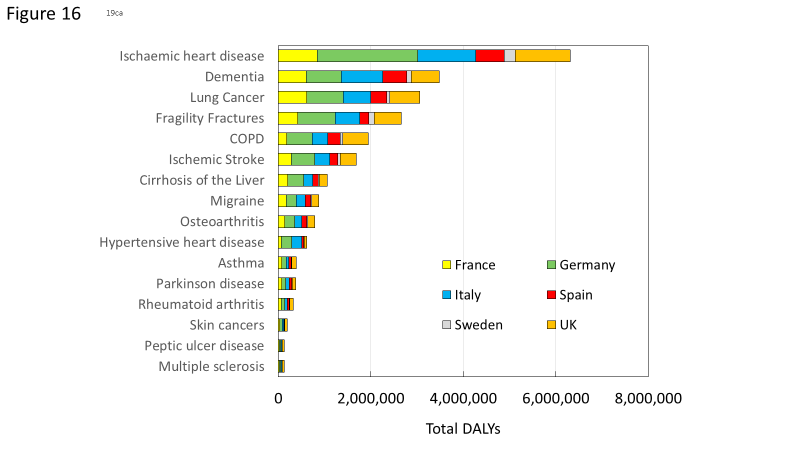
**

**Fig. A 16** Country Contribution to Total DALYs by Disease

**
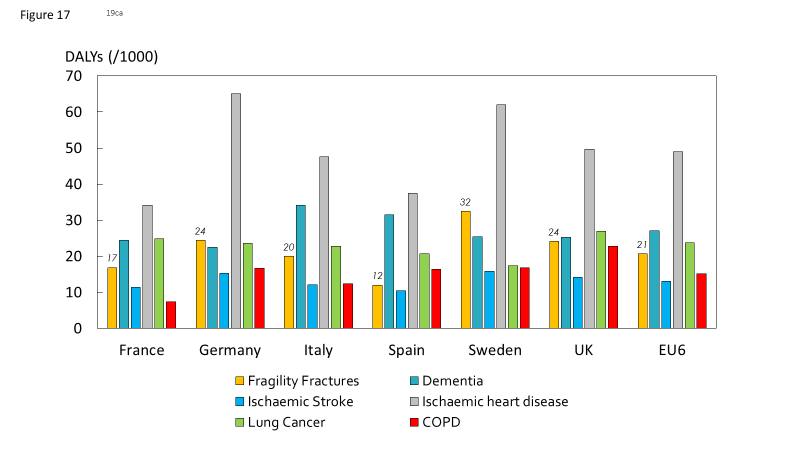
**

**Fig. A 17** Country Comparison - DALYs per 1000 People by Disease

**Comments**

These results are in line with the previous DALY comparison estimates from Johnell et al 2006 [70], and show that the osteoporosis burden has grown significantly over the past 10 years. The data for the figures shown is provided below.

**Supporting data**

**Table A 23** Total DALYs – Both Genders

| **Disease** | **France** | **Germany** | **Italy** | **Spain** | **Sweden** | **UK** | **Total** |
| --- | --- | --- | --- | --- | --- | --- | --- |
| Multiple sclerosis | 19,127 | 35,331 | 17,886 | 8,890 | 5,362 | 38,181 | 124,777 |
| Peptic ulcer disease | 14,873 | 41,260 | 16,372 | 9,428 | 4,951 | 40,704 | 127,589 |
| Melanoma and other skin cancers | 33,195 | 50,218 | 32,244 | 16,391 | 10,031 | 44,900 | 186,979 |
| Rheumatoid arthritis | 60,201 | 75,209 | 60,483 | 41,305 | 9,299 | 77,425 | 323,921 |
| Parkinson disease | 69,177 | 87,849 | 82,511 | 49,744 | 12,017 | 70,112 | 371,409 |
| Asthma | 77,558 | 94,331 | 56,518 | 50,177 | 13,327 | 91,344 | 383,255 |
| Hypertensive heart disease | 59,759 | 225,050 | 215,324 | 46,498 | 9,695 | 56,834 | 613,160 |
| Osteoarthritis | 142,405 | 212,026 | 152,026 | 105,817 | 21,262 | 153,251 | 786,788 |
| Migraine | 173,153 | 212,722 | 191,854 | 121,083 | 21,054 | 157,503 | 877,369 |
| Cirrhosis of the Liver | 204,023 | 340,499 | 197,945 | 120,945 | 19,454 | 175,447 | 1,058,313 |
| Ischemic Stroke | 280,063 | 508,748 | 318,263 | 172,317 | 59,861 | 341,093 | 1,680,345 |
| COPD | 181,832 | 554,887 | 325,924 | 269,982 | 63,712 | 548,267 | 1,944,604 |
| Fragility Fractures | 416,588 | 814,059 | 525,336 | 198,670 | 122,933 | 579,722 | 2,657,308 |
| Lung Cancer | 612,608 | 787,281 | 597,633 | 342,673 | 65,612 | 646,353 | 3,052,161 |
| Dementia | 604,054 | 751,188 | 897,210 | 520,414 | 96,455 | 606,875 | 3,476,197 |
| Ischemic heart disease | 841,046 | 2,172,490 | 1,249,338 | 618,672 | 234,596 | 1,195,073 | 6,311,215 |
| All Causes | 11,134,742 | 16,761,088 | 12,189,447 | 7,164,753 | 1,823,641 | 11,869,537 | 60,943,207 |

**Table A 24** DALYs per 100,000 – Both Genders

| **Diseases** | **France** | **Germany** | **Italy** | **Spain** | **Sweden** | **UK** | **EU6** |
| --- | --- | --- | --- | --- | --- | --- | --- |
| All Causes | 451 | 502 | 464 | 434 | 482 | 494 | 474 |
| Fragility Fractures | 17 | 24 | 20 | 12 | 32 | 24 | 21 |
| Melanoma and other skin cancers | 1 | 2 | 1 | 1 | 3 | 2 | 1 |
| Dementia | 24 | 22 | 34 | 32 | 25 | 25 | 27 |
| Parkinson disease | 3 | 3 | 3 | 3 | 3 | 3 | 3 |
| Ischemic Stroke | 11 | 15 | 12 | 10 | 16 | 14 | 13 |
| Multiple sclerosis | 1 | 1 | 1 | 1 | 1 | 2 | 1 |
| Migraine | 7 | 6 | 7 | 7 | 6 | 7 | 7 |
| Hypertensive heart disease | 2 | 7 | 8 | 3 | 3 | 2 | 5 |
| Ischemic heart disease | 34 | 65 | 48 | 37 | 62 | 50 | 49 |
| Lung Cancer | 25 | 24 | 23 | 21 | 17 | 27 | 24 |
| COPD | 7 | 17 | 12 | 16 | 17 | 23 | 15 |
| Asthma | 3 | 3 | 2 | 3 | 4 | 4 | 3 |
| Peptic ulcer disease | 1 | 1 | 1 | 1 | 1 | 2 | 1 |
| Cirrhosis of the liver | 8 | 10 | 8 | 7 | 5 | 7 | 8 |
| Rheumatoid arthritis | 2 | 2 | 2 | 3 | 2 | 3 | 3 |
| Osteoarthritis | 6 | 6 | 6 | 6 | 6 | 6 | 6 |

**11: Productivity loss**

**Background**

Most fractures occur in elderly retired patients. However, in Sweden about 20% of fractures occur in pre-retirement ages [14]. If a fracture patient is still employed he/she most likely would be on sick leave during the recovery phase. Work absence both impacts the individual’s income and creates a societal cost due to the loss in productivity. This metric uses survey responses from the International Costs and Utilities Related to Osteoporotic Fractures Study (ICUROS) to estimate the number of sick days in the year after an osteoporotic fracture.

**Methods**

The ICUROS collected data on fracture consequences over 18 months after fracture. The study enrolled 5,869 patients in 11 countries [53, 71, 72]. About 19% of the study population indicated they were employed before their fracture. The same study design as ICUROS was used in a Swedish study that enrolled 635 patients [42]. In these studies, patients were asked how many days of sick leave they had taken at 4, 12 and 18 months as a consequence of the fracture. For the purpose of this analysis, the results are presented as total days of sick leave the year after hip, vertebral or other (humeral or wrist) fracture. The within-country sample size is modest and only a minority of the patients were at working ages (below 66 years). Therefore, country-specific estimates were not included. Cross country estimates were calculated using data from six of the European ICUROS countries (Austria, Estonia, France, Italy, Sweden, and the UK) and dubbed ICUROS Europe. Russia and Lithuania were excluded from the European estimate because of the low hospitalization rate in relation to fracture which cannot be considered representative for a general European setting. For consistency, Lithuania and Russia were also excluded from the other metrics which used ICUROS data. Spain was dropped from this metric due to lack of data. Fracture rates for the EU6 in 2017 was derived from the burden model and multiplied with average sick days by fracture site, in order to estimate the average number of sick days taken due to fragility fracture, per 1000 people.

**Results**

Estimates of the annual sick leave by fracture type for ICUROS Europe are shown in Fig. A 18. Hip fractures account for the largest number of sick leave days (42 days) followed by vertebral fracture (20 days). Fig. A 19 shows the number of days taken due to fragility fracture, per 1000 people in each of the EU6 countries. Sick days taken in 2017, by non-retired individuals in the EU6 totalled 7,615,719 days. “Other” major osteoporotic fractures (MOFs) occur significantly more often than hip or clinical vertebral fractures, and therefore resulted in the highest estimated number of sick days taken due to fragility fracture, per 1000 people in all countries, with Sweden showing the highest estimate overall in the EU6. There were no significant differences between sick leave taken by men and women with hip fractures, nor between sick leave taken by hip fracture patients with or without previous fracture.


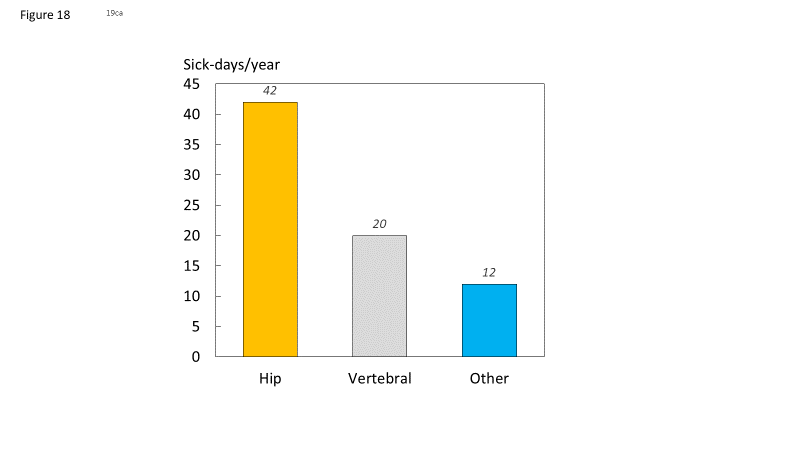


**Fig. A 18** Annual sick leave (days) by fracture type (ICUROS Europe)

*ICUROS Europe= Austria, Estonia, France, Italy, Sweden, and the UK


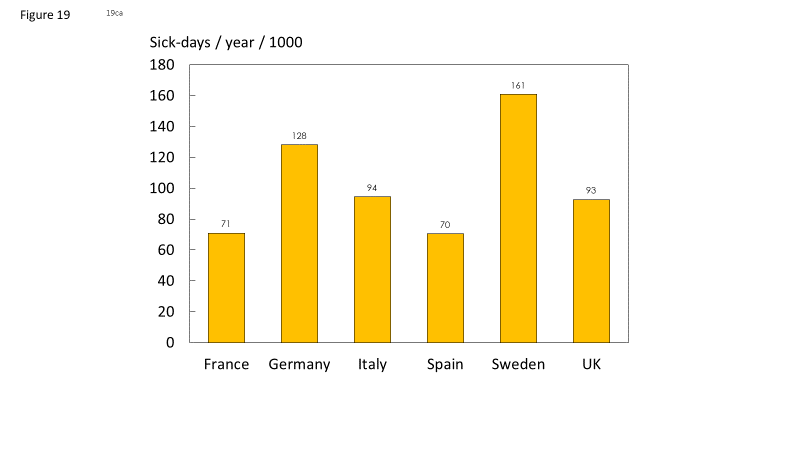


**Fig. A 19** Total sick days taken in the EU6 due to fragility fracture, in 2017

**Comments**

The data included only individuals who indicated they were employed at the time of fracture. As the age limit for retirement rises, it is likely that average productivity loss per capita will increase as people are able to live and work for longer.

**12:** **Caregiver burden**

**Background**

A significant social cost associated with chronic diseases such as osteoporosis is the burden imposed on informal caregivers such as family members. Continued care provided at home can put physical, emotional and financial strain on relatives who need to take care of osteoporotic fracture patients [37, 73]. This metric uses data collected in the International Costs and Utilities Related to Osteoporotic Fractures Study (ICUROS) [53, 71, 72] to determine the caregiver burden due to osteoporotic fracture, in terms of hours of care provided by relatives.

**Methods**

The ICUROS collected data on fracture consequences over 18 months after fracture. The study enrolled 5,869 patients in 11 countries [53, 71]. The same study design as ICUROS was used in a Swedish study that enrolled 635 patients [42]. In these studies, patients were asked how many hours per week they received care from relatives after their fracture. This question was asked at 4, 12 and 18 months. For the purpose of this analysis, the results are presented as total hours of care for the year given by relatives after an individual’s hip, vertebral or other (humeral or wrist) fracture. Cross country estimates were calculated using data from seven of the European countries from the studies (Austria, Estonia, France, Italy, Spain, Sweden, and the UK) and dubbed ICUROS Europe. To be consistent with the estimation methods across these metrics, Lithuania and Russia were dropped.

**Results**

Estimates for the total hours of care provided by relatives, per individual per year after a given fracture, are shown in Fig. A 20. These estimates represent the combined average of ICUROS Europe. The results indicate that hip fractures are associated with the largest caregiver burden (370 hours per year) among major osteoporotic fractures, followed by vertebral fractures (263 hours per year). Care from relatives for hip fractures varies greatly by country. In Fig. A 21, estimates are shown for the hours of care provided by relatives for hip fracture patients in ICUROS Europe, as well as five of the countries of interest. Within the five countries of interest, Spain and Italy have the highest caregiver burden, with averages of 744 hours and 652 hours per year spent caring for individuals with osteoporotic hip fractures, respectively. The UK (227 hours) and Sweden (115 hours) have considerably lower averages. There were no significant differences in care from relatives between men and women, nor between patients with or without a previous fracture. Fig. A 22 shows the annual hours of care provided after hip fractures per 1000 individuals. Since Italy and Spain have the highest average hours of care per individual hip fracture, this trend is also present in the hours of care provided per 1000 individuals, with 882 and 756 hours per 1000 people per year, respectively. Sweden was estimated to have more hours of care provided (191 hours per 1000 people per year) than France (138 hours per 1000 people per year), which is driven by fracture incidence and population.


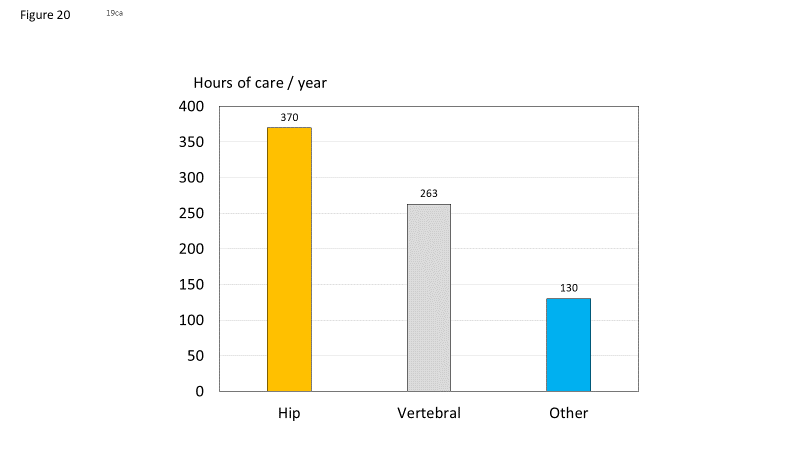


**Fig. A 20** Average Annual Hours of Care by Relatives by Fracture Type in seven EU countries (ICUROS Europe)


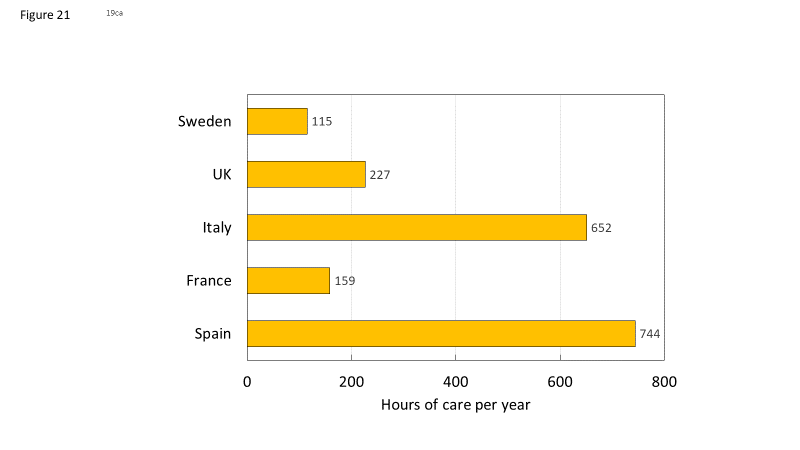


**Fig. A 21** Average Annual Hours of Care by Relatives after Hip Fracture by Country

*ICUROS Europe= Austria, Estonia, Spain, France, Italy, Sweden, and the UK*

**
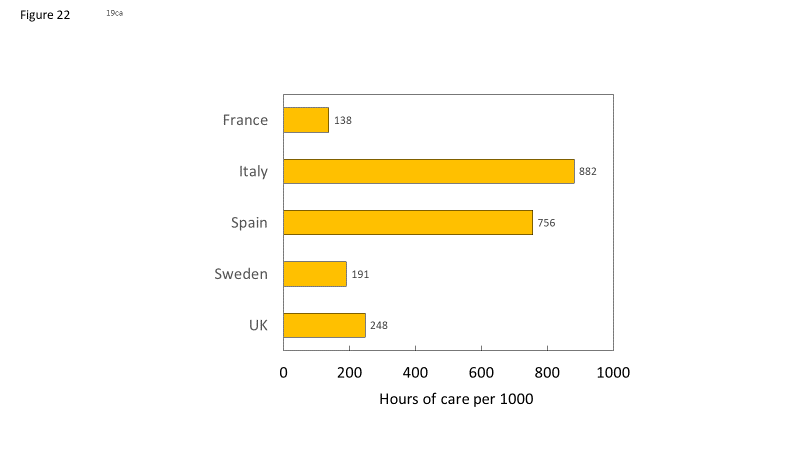
Fig. A 22** Annual hours of care provided in the year after hip fracture per 1000 people, by country

**Comments**

Care provided informally by relatives represents a significant investment of time and energy. Measuring this burden gives individual countries a better understanding of the indirect costs associated with osteoporosis.

**13:** **Independent living**

**Background**

One major potential consequence of a fragility fracture is the long-term impact on independence. The fracture can result in a loss of mobility, the ability to take care of oneself, and may require the individual to move into long-term care (LTC) or elderly care services. This metric uses data from the International Costs and Utilities Related to Osteoporotic Fractures Study (ICUROS) to estimate the proportion of patients that move to a long-term care facility after a fracture.

**Methods**

The ICUROS collected data on fracture consequences over 18 months after fracture. The study enrolled 5,869 patients in 11 countries [53, 71]. The same study design as ICUROS was used in a Swedish study that enrolled 635 patients [42]. In these studies, patients were asked if they had moved to LTC as a consequence of the fracture. The answers were used to estimate the proportion of patients that had moved from living at home to LTC at 12 months after hip, vertebral or other (humeral or wrist) fracture. Individuals who were living in LTC before their fracture were excluded. Cross country estimates were calculated using data from six of the European countries in these studies (Austria, Estonia, France, Italy, Spain and Sweden) and dubbed ICUROS Europe. To be consistent with the estimation methods across the metrics which used ICUROS, Lithuania and Russia were dropped. Due to lack of data, the UK was excluded.

**Results**

Fig. A 23 shows the proportion of patients residing in an LTC facility at 12 months after fracture by fracture type. LTC use varies greatly, depending on the fragility fracture and the age of the individual. Hip fractures correspond with the largest proportion of people moving to LTC in all six countries. Comparing LTC use after a hip fracture for different countries (Fig. A 24) shows that Italy has a large percentage (24.9) of hip fracture patients who enter long term care by 12 months, while the average for ICUROS Europe is lower (9.1%). Fig. A 25 shows that female hip fracture patients had a higher probability (12.0%) of moving to LTC compared to male patients (2.3%). There was no significant difference in the use of LTC between fracture patients with or without a previous fracture. Fig. A 26 shows the percentage of individuals in LTC following a hip fracture, based on 10-year age groups. The percentage in LTC 12 months after a hip fracture increases significantly by age group.

**
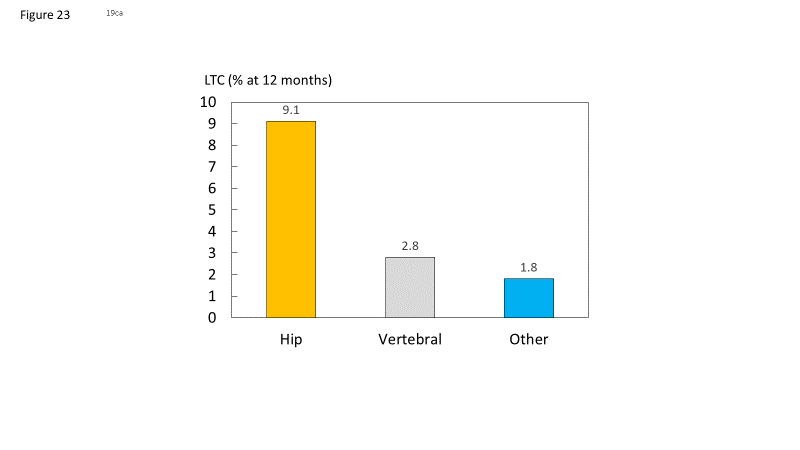
**

**Fig. A 23** Percentage (%) in long term care (LTC) at 12 months by fracture site in 6 EU countries (ICUROS Europe)


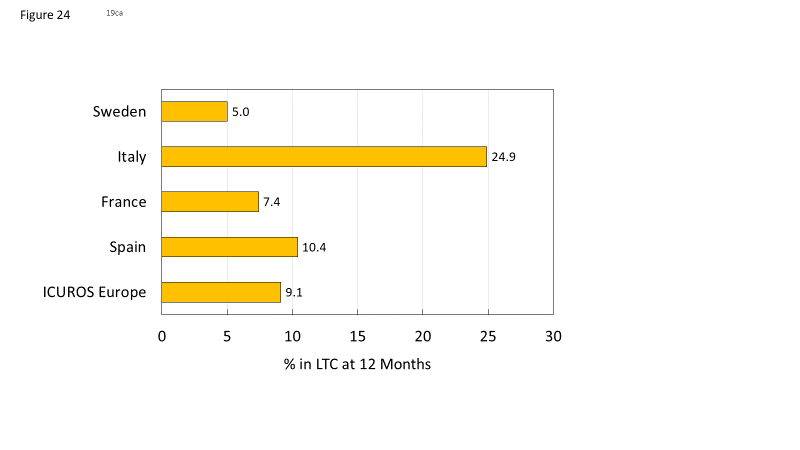


**Fig. A 24** Percentage (%) in long term care (LTC) with hip fracture at 12 months by country (ICUROS Europe = Austria, Estonia, Spain, France, Italy, Sweden)


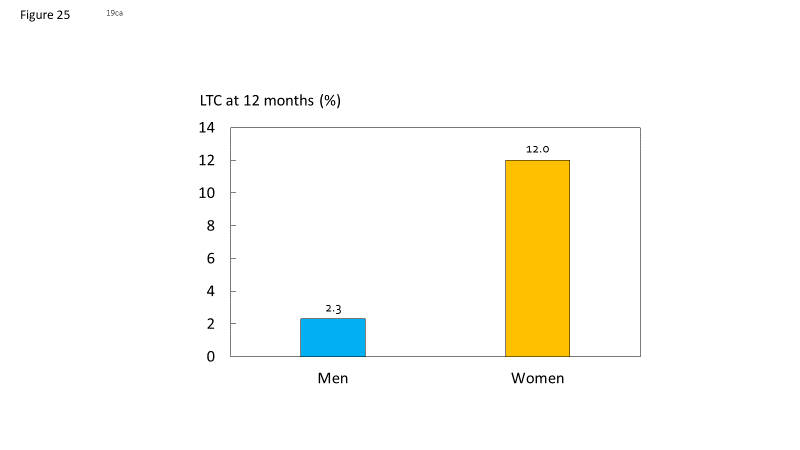


**Fig. A 25** Percentage (%) in long term care (LTC) at 12 months following a hip fracture by sex in five ICUROS countries (Austria, Estonia, Spain, France, Italy).

**
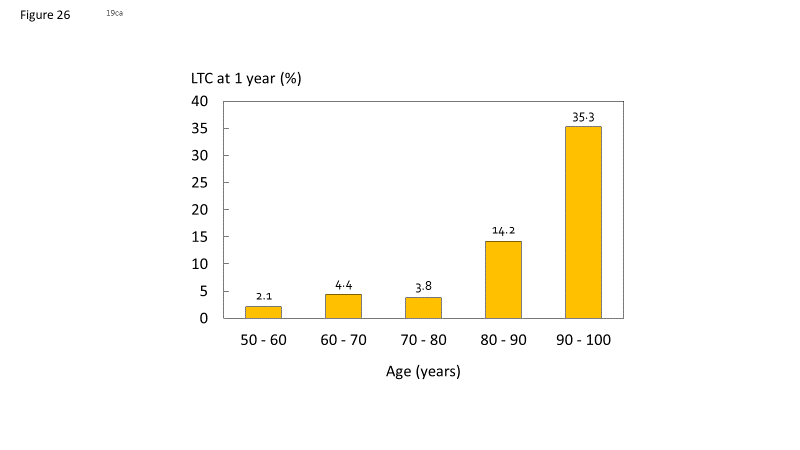
Fig. A 26** Percentage in LTC 1 year after hip fracture, by age group

**Comments**

Long-term care represents a large direct non-medical cost for a social health care system. Furthermore, each country provides these services at varying levels. Being able to measure the impact that osteoporotic fractures have on the budget for long term or elderly care can help policy makers in their decision making.

**14: Pharmacological treatment gap**

**Background**

The treatment gap in osteoporosis has been estimated previously for the EU6 using IMS Health drug sales data up to the year 2010 [37]. This metric provides an update of the treatment gap in 2017.

**Methods**

National data on number of patients treated are currently not readily available in many European countries. In the absence of more granular data an alternate approach using international sales data on volume (standard units) and price (€) from IMS Health have previously been used to estimate the treatment gap in osteoporosis [8, 37].

The same method was applied for this analysis. In brief, the method includes the following steps to estimate treatment gap.

1. Using IMS sales data to estimate how many treatment years the sales volume, by year, cover.
2. Adjusting for low adherence, i.e., the average time on treatment per patient over a year is less than 365 days. The corrective factor was derived from Swedish register data and was 77%. After this correction the number of patients potentially treated during a year is obtained.
3. The proportion of the population eligible for treatment was determined using an intervention threshold based on data from FRAX of the 10-year probability of a major osteoporotic fracture of women in the EU6 countries.
4. The intervention threshold set was a 10-year probability of a major osteoporotic fracture equivalent to an age-matched female with a fragility fracture [74]
5. The treatment gap is obtained by dividing the patients potentially treated with the number of individuals considered to be eligible for treatment.

Also, because the IMS sales data do not differentiate by sex or age, it was assumed that 85% of sales were given to women (based on Swedish data) and all osteoporotic drugs were only given to patients age 50 years or older.

The analysis included data on sales related to osteoporosis drugs for the year 2017. These included: bisphosphonates, denosumab, parathyroid hormones (PTHs), selective oestrogen receptor modulators (SERMs) and strontium ranelate. Note that hormone replacement therapy was not included.

IMS sales data is the best source of comparative data on sales of pharmaceuticals at an international level that allows this type of analysis. However, it is also associated with some shortcomings. For example, previously it was shown that compared to Swedish prescription data the sales data seem to yield a 20% higher treatment uptake implying an underestimation of the treatment gap. [8].

**Results**

Fig. A 27 shows the number of patients that could be treated in 2017 and 2010 based on IMS sales data, compared to the remaining number of persons eligible for treatment. The average treatment gap (Table A 25 and Table A 26) in EU6 in year 2017 is estimated to be 73% for women and 63% for men. Compared to the analysis from year 2010 this is marked increase (17% and 16% points) in the treatment gap.

The treatment gap varies between countries and the highest treatment gap for women can be found in Germany where less than 20% of the population eligible for treatment receives an osteoporotic drug. Germany also shows the highest treatment gap for men. UK has the smallest treatment gap (64%) in women and men (43%).

**
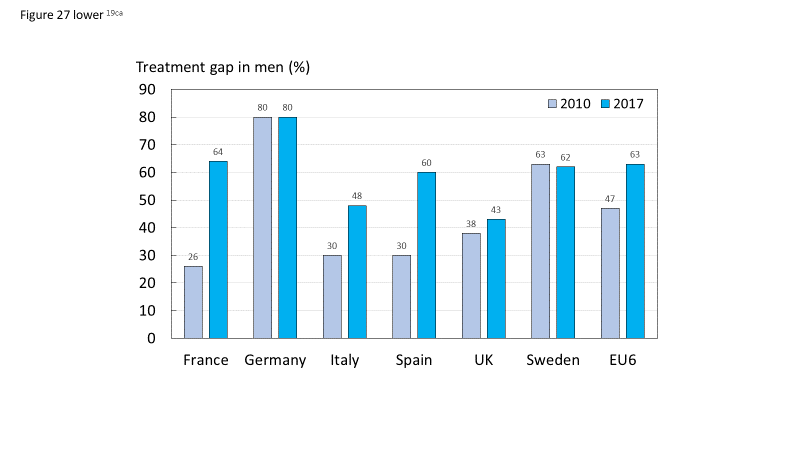
**
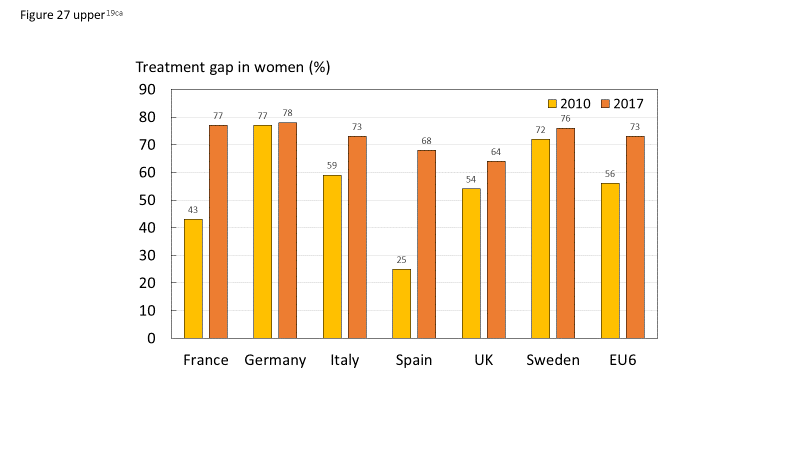


**Fig. A 27** The treatment gap in women (upper panel) and men (lower panel) based on potential number of patients treated relative to eligible number of individuals above the intervention threshold.

Note: There was an error in the 2010 estimate for men in the UK [37], which is corrected in the figure above

**Table A 25** Number of women eligible for treatment, treated and treatment gap in 2017

| **Country** | **Number potentially treated (1000s)** | **Number exceeding fracture risk threshold (1000s)** | **Difference (1000s)** | **Treatment gap (%)** | **Treatment gap, 2010 (%) [37]** |
| --- | --- | --- | --- | --- | --- |
| France | 635 | 2,758 | 2,123 | 77 | 43 |
| Germany | 692 | 3,542 | 2,849 | 80 | 77 |
| Italy | 784 | 2,944 | 2,160 | 73 | 59 |
| Spain | 647 | 2,013 | 1,366 | 68 | 25 |
| UK | 971 | 2,690 | 1,720 | 64 | 54 |
| Sweden | 95 | 392 | 296 | 76 | 72 |
| EU6 | 3,825 | 14,340 | 10,515 | 73 | 57 |

**Table A 26** Number of men eligible for treatment, treated and treatment gap in 2017

| **Country** | **Number potentially treated (1000s)** | **Number exceeding fracture risk threshold (1000s)** | **Difference (1000s)** | **Treatment gap (%)** | **Treatment gap, 2010 (%) [37]** |
| --- | --- | --- | --- | --- | --- |
| France | 112 | 312 | 200 | 64 | 26 |
| Germany | 122 | 619 | 497 | 80 | 80 |
| Italy | 138 | 265 | 127 | 48 | 30 |
| Spain | 114 | 289 | 175 | 60 | 20 |
| UK | 171 | 301 | 130 | 43 | 38* |
| Sweden | 17 | 45 | 28 | 62 | 63 |
| EU6 | 675 | 1,669 | 994 | 60 | 49 |

** Note: There was an error in the 2010 estimate for men in the UK leading to a negative estimate. This is corrected in the table above*

**Comments**

This analysis shows that treatment of osteoporosis is poor in the EU6 countries and less than 30% of women and 40% of men receives the treatment that they need. Lack of osteoporosis treatment could lead to increased risk of fracture, increased morbidity and increased costs to society. Note that this analysis assumes that interventions are directed only to individuals at high risk which is unlikely to be invariable. Thus, the estimate of the treatment gap is conservative.

**15: Fracture treatment gap**

**Background**

An alternative approach to assessing the treatment gap with fragility fractures is to estimate the proportion of patients starting a pharmacological treatment after a fracture. National estimates can vary greatly, and to conduct such analysis, real world data on fractures and drug use is required for each country. There is a small but growing body of literature that focuses on the post-fracture treatment gap.

**Methods**

A structured review of published literature was conducted to find country-specific estimates for the gap in treatment following an osteoporotic fracture. Estimates for both hip and all osteoporotic fractures were found for select countries from multiple sources. Available estimates were gathered from a mix of literature, public reports (France [75] and the UK [76]), data on file at UCB (Spain) and data on file at Quantify Research (Sweden). No estimate which met the criteria could be found for Italy or Germany.

**Results**

The percentage of women starting an osteoporosis-specific pharmacological treatment within a year of an osteoporotic fracture or a hip fragility fracture is shown in Fig. A 28. The analytic methods vary between the estimates making direct comparisons difficult. However, the post-fracture treatment gap can be considered large irrespective of country. In the category of “all osteoporotic fractures,” France has the highest gap in treatment, with 85% of individuals not receiving osteoporosis medications after an osteoporotic fracture. Spain has the lowest treatment gap overall, with 72% of patients not receiving treatment. Hip fractures in Sweden are met with the highest gap in treatment (84%) while in the UK, only 49% of hip fracture victims do not receive treatment after their fracture. The treatment gap is also likely to vary between gender, fracture types and treatment history. Such detailed information is not currently available for all the EU6 countries. Table A 27 presents the treatment gaps after hip or any osteoporotic fracture, by country.


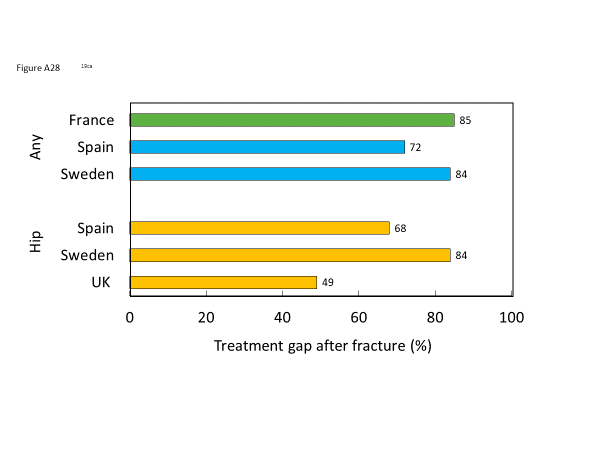


**Fig. A 28** Treatment gaps after hip fracture or any osteoporotic fracture, by country

**Table A 27** Treatment gaps after hip or any osteoporotic fracture, by country

| **Country** | **type** | **gap** | **uptake** | **year** |
| --- | --- | --- | --- | --- |
| *UK* | *hip* | 49% | 51% | 2015 |
| *Sweden* |  | 84% | 16% | 2013/2014 |
| *Spain* |  | 89% | 11% | 2015 |
|  | *any* |  |  |  |
| *Spain* |  | 95% | 5% | 2015 |
| *France* |  | 85% | 15% | 2016 |
| *Sweden* |  | 84% | 16% | 2013/2014 |

**Comments**

The estimates provided include only the literature/data, which met the definition of receiving treatment up to one year after fracture. Other literature sources which did not meet the criteria of the question were excluded.

**16: Treatment gap by fracture type**

**Background**

Pharmacological intervention is an important approach to prevent fragility fractures. Yet, this is another area within osteoporosis where a significant gap (patients receiving treatment compared to those eligible for treatment) exists [77]. Previous studies have however relied on sales data rather than drawing on national claims and prescription data.

**Methods**

Patients with a fracture in 2013 and 2014 were identified from the Swedish National Patient Register (NPR). Their treatment history was extracted from the Swedish Prescribed Drug Register. Patients were defined as treatment-experienced if they had filled at least one prescription for any osteoporosis drug (including HRT) during the three years prior to the fracture. See Table A 28 for a list of products and corresponding ATC codes which defined treatment exposure. If no prescription had been filled, the patient was defined as treatment naïve. The number of patients who were treated (at least one prescription for any osteoporosis drug excluding HRT) the following year after fracture was reported together with the number of deaths. See Table A 29 for a list of products and corresponding ATC codes which defined patients as treated following fracture. Patients with a diagnosis of Paget’s disease or malignancies anytime during the study period (2010-2015) were excluded from the analysis.

**Table A 28** Treatments and ATC codes that defined treatment exposure

| **Treatment** | **ATC codes** | **Products** |
| --- | --- | --- |
| *Alendronate* | M05BA04 | All |
| *Alendronate + cholecalciferol* | M05BB03 | All |
| *Denosumab* | M05BX04 | Prolia only |
| *Ibandronate* | M05BA06 | Bonviva only |
| *Risedronate* | M05BA07 | All |
| *Risedronate + calcium* | M05BB02 | All (no longer on the market) |
| *Risedronate + calcium + cholecalciferol* | M05BB04 | All (no longer on the market) |
| *Zoledronate* | M05BA08 | Aclasta only (prescriptions only) |
| *Etidronate* | M05BA01 | All (no longer on the market) |
| *Etidronate + calcium* | M05BB01 | All (no longer on the market) |
| *Strontium ranelate* | M05BX03 | All (no longer on the market) |
| *Raloxifene* | G03XC01 | All |
| *Bazedoxifene* | G03XC02 | All |
| *Teriparatide* | H05AA02 | All |
| *Parathyroid hormone 1-84* | H05AA03 | All (no longer on the market) |
| *Hormone replacement therapy* | G03CA03, G03CA04, G03FA01, G03FA12, G03FA17, G03FB05, G03FB06 | Systemic HRT with indication for osteoporosis only, i.e.: Progynon; Femanest; Estradot 50/75/100 µg/h; Evopad 50/75/100µg/h; Evorel 50/75/100µg/h; Livial; Tibolon Orifarm; Activelle; Kliogest; Cliovelle; Femanor; Estalis; Duova; Indivina; Angemin; Novofem; Trisekvens; Femasekvens; Sequidot; Divina Plus; Trivina, |

**Table A 29** Treatments and ATC codes that defined post-fracture patients as treated

| **Treatment** | **ATC codes** | **Products** |
| --- | --- | --- |
| *Alendronate* | M05BA04 | All |
| *Alendronate + cholecalciferol* | M05BB03 | All |
| *Ibandronate* | M05BA06 | Bonviva only |
| *Risedronate* | M05BA07 | All |
| *Risedronate + calcium* | M05BB02 | All (no longer on the market) |
| *Risedronate + calcium + cholecalciferol* | M05BB04 | All (no longer on the market) |
| *Etidronate* | M05BA01 | All (no longer on the market) |
| *Etidronate + calcium* | M05BB01 | All (no longer on the market) |
| *Zoledronate* | M05BA08 | Aclasta only (prescriptions only) |
| *Denosumab* | M05BX04 | Prolia only |
| *Strontium Ranelate* | M05BX03 | All (i.e. Protelos) |
| *Raloxifene* | G03XC01 | All |
| *Teriparatide* | H05AA02 | All (i.e. Forsteo) |

**Results**

At the time of fracture, a majority of women (75-88%) and men (95-98%) were treatment naïve. Fig. A 29 and Fig. A 30 show the treatment gap following fracture by treatment experience in women and men, respectively. The treatment gap in treatment naïve women within a year following a hip, a MOF or any osteoporotic fracture was around 90%. The treatment gap following a vertebral fracture was 74%. A similar pattern was observed in the male population although treatment gaps were in general higher. Treatment gap in treatment naïve men following a hip, MOF or any osteoporotic fracture was around 95%. The corresponding treatment gap following vertebral fracture was 89%. Fig. A 31 shows the treatment gap following fracture independent of treatment history. In women, the treatment gap was around 84% within one year following fracture with the exception of vertebral fractures (67%). A similar pattern was observed in the male population. Complete results can be found in Table A 30.

**
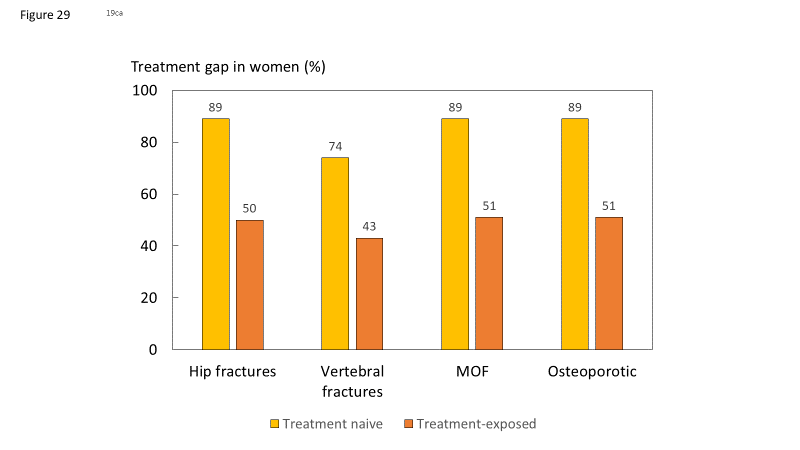
**

**Fig. A 29** Treatment gap in women within one year from fracture in Sweden, by fracture site and treatment history


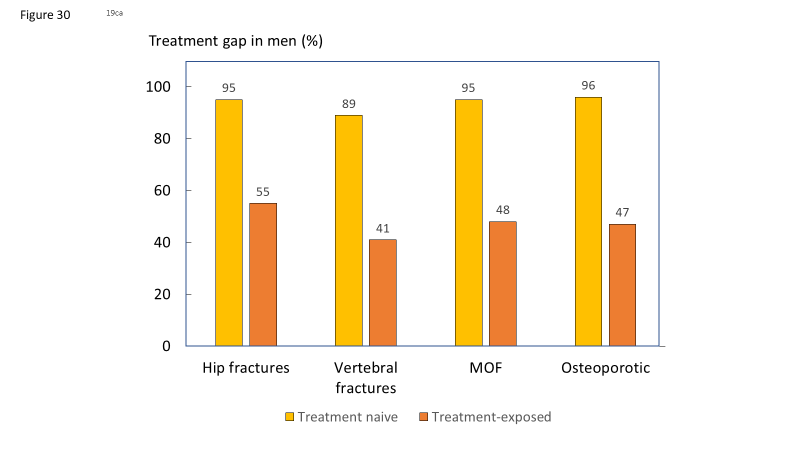


**Fig. A 30** Treatment gap in men within one year from fracture in Sweden, by fracture site and treatment history


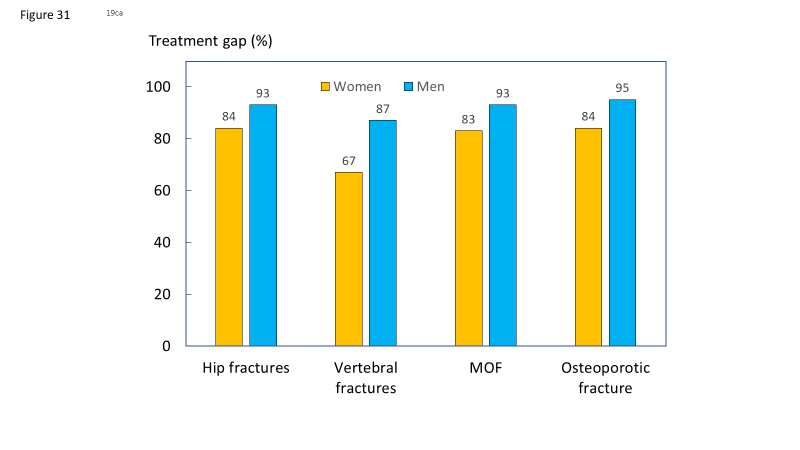


**Fig. A 31** Treatment gap (independent of treatment history) within one year from fracture in Sweden, by fracture site and gender

**Table A 30** Number of patients treated following fracture (by type) independent of treatment history, in 2013 and 2014, in Sweden

| **Category** | **N** | **No treated within a year** | **%** | **No of deaths within a year** | **No (and %) that started treatment and died within a year** |
| --- | --- | --- | --- | --- | --- |
| **Women** |  |  |  |  |  |
| **Hip fractures** | 19,550 | 3,086 | 15.8% | 4,164 | 200, 4.8% |
| **Vertebral fractures** | 6,702 | 2,239 | 33.4% | 775 | 120, 15.5% |
| **MOF** | 56,609 | 9,701 | 17.1% | 6,573 | 416, 6.3% |
| **Osteoporotic fractures** | 76,974 | 12,426 | 16.1% | 8,193 | 147, 7.4% |
| **Men** |  |  |  |  |  |
| **Hip fractures** | 8,100 | 534 | 6.6% | 2,296 | 45, 2.0% |
| **Vertebral fractures** | 3,470 | 460 | 13.3% | 480 | 44, 9.2% |
| **MOF** | 18,734 | 1,260 | 6.7% | 3,302 | 530, 6.5% |
| **Osteoporotic fractures** | 32,027 | 1,661 | 5.2% | 4,183 | 122, 2.9% |

**Comments**

This analysis shows that the post fracture treatment gap in Sweden is large. That is, few patients with a fragility fracture is prescribed a drug for reducing the risk of further fractures. Treatment initiation is more common following a vertebral fracture compared to other fracture types (hip, NHNS and MOF) in both women and men.

**17: Fracture risk assessment**

**Background**

In order to identify individuals for treatment who have the highest risk of fragility fracture, some form of fracture risk assessment is required. With the introduction of Bone Mineral Density (BMD) measurements, the first methods of assessing bone fragility in the elderly became available to medical professionals. However, there are a number of factors that are associated with an increased risk of fracture over and above that provided by BMD. This has led to the development of risk models, which incorporate several risk factors in order to improve the identification of patients at high risk of fracture in primary care and elsewhere [78]. This metric examines the availability of risk models in the six countries of interest, and whether official national guidelines are present for the use of fragility fracture risk assessments.

There are several existing models for risk assessment in Europe, however the most prominently used is FRAX. FRAX is a computer-based algorithm, developed in 2008, that calculates the probability of fracture in individuals using age, body mass index, BMD and dichotomized risk factors such as prior fragility fracture, parental history of hip fracture, tobacco usage, alcohol usage, rheumatoid arthritis and other causes of secondary osteoporosis [74]. The use of BMD measurements in the FRAX model is recommended but optional [79].

**Methods**

FRAX models are currently available for 64 countries. Country-specific FRAX models can be found on the official FRAX website [79] and are a software option for densitometers and other devices. Other risk assessment models, and guidelines for the use of risk models in each of the countries of interest were obtained from their respective health departments’ websites (sources listed in Table A 31).

**Results**

Table A 31 provides a summary of the access to FRAX and other risk assessment models and their guidelines in the EU6. Country-specific FRAX models exist in all 6 countries. Alternative assessment models are also recommended in Germany, Italy and the UK. The German DVO model, developed in 2006, is a Germany-specific risk assessment model which requires the use of BMD measurements [80, 81]. DeFra is an Italy-specific extension of the FRAX model, which allows for comparison of the BMD in different fracture sites and the inclusion of more data [82]. QFracture in the UK was developed in 2009 and uses variables which are readily available through healthcare records in the UK. It also utilizes a younger age range than FRAX [83].

Specific guidelines for the use of FRAX and other risk models were found on official national health service websites for all countries except for Italy. The Italian Ministry of Health did not recommend specific risk models but suggested that risk models could be useful in assessing the probability of fragility fracture. Other organizations like the Italian Society for Orthopedics and Traumatology recommended FRAX or DeFra specifically.

**Table A 31** Risk models & guidelines available in the countries of interest

| **Countries** | **FRAX Model available** | **Other Models** | **National Guidance** | **Comments** | **Source** |
| --- | --- | --- | --- | --- | --- |
| *France* | ✓ | - | ✓ |  | [84] |
| *Germany* | ✓ | DVO Model | ✓ |  | [80] |
| *Italy* | ✓ | FRAHS, DeFra | ✓ | FRAHS: FRAX-based | [85-87] |
| *Spain* | ✓ | - | ✓ |  | [88] |
| *Sweden* | ✓ | - | ✓ |  | [89] |
| *UK* | ✓ | QFracture | ✓ |  | [90] |

**Comments**

Fracture risk assessment models and guidelines for their use are readily available for the 5 EU countries and Sweden. Risk assessment models based on the FRAX model are available for 23 of the 28 EU member states, and 64 countries worldwide.

**18: Use of FRAX**

**Background**

FRAX was launched in 2008 in all EU6 countries. In 2011, Hernlund et al. [37] summarized the use of FRAX in the European Union and this report provides an update of FRAX use in 2017.

**Methods**

Number of calculations per year was retrieved from the FRAX website and was thereafter combined with population statistics to determine the number of calculations per million in the general population per year. The statistics is based on all usage since the data do not provide the reason for use (e.g. patient assessment, education or research).

**Results**

The web-based usage of FRAX is shown in Fig. A 32. The UK and Sweden had the highest usage of FRAX, whereas the lowest uptake was seen in Germany. Considering all countries in the EU6 the usage of FRAX increased by almost 75% in 2017 compared to 2011. The highest increase was seen in the UK, France and Sweden (~100%), whereas in both Germany and Italy the usage of FRAX decreased in 2017 compared to 2011. Table A 32 presents the FRAX calculations by country of origin in the EU6.

**
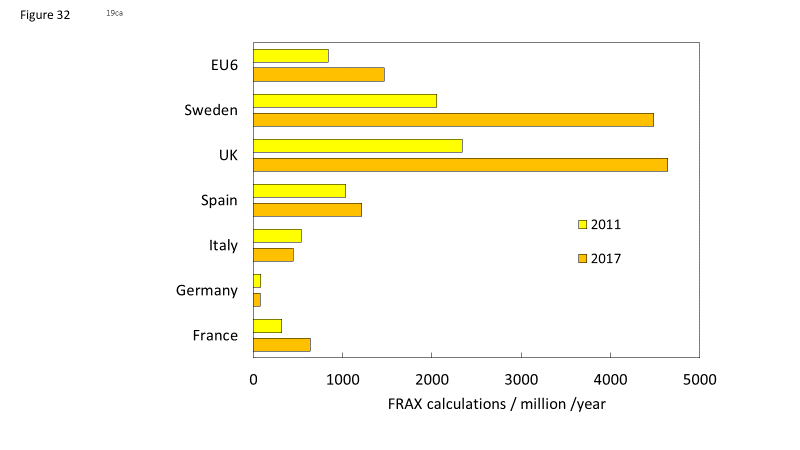
**

**Fig. A 32** FRAX calculations by URL source per million in the general population Nov 2010-2011 and April 2017-2018

**Comments**

The webpage usage of FRAX has increased in the EU6 in 2017 compared to 2011. That the usage of FRAX has decreased in both Italy and Germany may relate to the availability of other risk models such as the German specific DVO model and DeFra in Italy.

Note that these data do not take account of FRAX calculations made by densitometers, iPhones and hand-held calculators.

**Supporting data**

**Table A 32** FRAX calculations by country of origin in the EU6

| **Country** | **Calculations/year** | **Population (000)** | **Calculations/million/yr (2017)** | **Calculations/million/yr (2011) [37]** | **Difference (%)** |
| --- | --- | --- | --- | --- | --- |
| France | 42,727 | 67,059 | 637 | 318 | 100 |
| Germany | 6,454 | 84,312 | 77 | 82 | -7 |
| Italy | 27,537 | 61,420 | 448 | 535 | -16 |
| Spain | 57,985 | 47,876 | 1,211 | 1,034 | 17 |
| UK | 315,051 | 67,842 | 4,644 | 2,337 | 99 |
| Sweden | 45,588 | 10,167 | 4,484 | 2,052 | 119 |
| EU6 | 495,342 | 338,677 | 1,463 | 838 | 74 |

**19:** **Fracture Liaison Service impact**

**Background**

Fracture Liaison Services (FLS) function as a first line of defence against repeat osteoporotic fractures by identifying at-risk patients and referring them to specialists for assessment and preventative treatment. The FLS model was first introduced in teaching hospitals in Scotland and has grown in popularity around the world due to its effectiveness in preventing secondary fractures, and the lower need for doctors in the administration of post-fracture preventative services [91]. A growing body of evidence suggests that FLS is a cost-effective organizational form that reduces the risk of fracture, mortality and increases adherence to treatment [92-98]. This metric summarizes current evidence of FLS patient identification and evaluation, treatment use, adherence, fracture risk, and mortality as well as economic impact.

**Methods**

The evidence summary was based on published systematic literature reviews evaluating FLS programs. A pragmatic search identified three recent relevant large-scale systematic literature reviews. Wu et al conducted a meta-analysis including 159 publications to estimate the impact of FLS on select patient outcomes: the rate of BMD testing, treatment initiation, adherence to treatment, re-fracture and mortality [99]. Another study by Wu et al focused on the economic impact of FLS [100]. A study by Walters et al reviewed the evidence of different model types (A to D) of FLS on fracture risk, DXA referrals, and other patient outcomes [98].

**Results**

The main results of the first study by Wu et al 2018 are shown in Table A 33. In the meta-analysis, introduction of FLS was associated with greater than 20% improvements, on average, in the rate of individuals getting BMD tests, starting treatment and adhering to treatment. There was also a small but significant average reduction in the re-fracture and mortality rates.

Estimates from Sweden and the UK for the economic impact of FLS are shown in Table A 34. The Swedish study focused on a hypothetical cohort of 1000 elderly women over 10 years and found that the FLS treatment group saved 19 QALYs or 40 life years in total, resulting in an incremental cost effectiveness ratio (the cost per QALY gained) of €14,029 per QALY gained. In the UK, a study involving an NHS hospital found that FLS were cost effective for both male and female patients, with ICERs of £19,955 (about €22,700) per QALY and £20,421 (about €26,600) per QALY gained, respectively. Another UK study looked at the cost savings of introducing an FLS in an area with 1000 hypothetical patients and found that the FLS saved £21,000 (about €23,800) in total over their lifetimes, with 18 subsequent fractures estimated to be prevented, using the least favourable efficiency data.

Even though the meta-analysis showed an overall positive impact of FLSs, it did not consider that there are several types of FLS models which are likely to be associated with different outcomes. For example, some FLS only identify patients and inform them without taking any further actions whereas other more complete FLS identify, investigate, treat and monitor the patient. There has been no direct quantitative comparison between the different models made, but an analysis by Walters et al 2017 [98] indicated that the FLS models which take full responsibility for investigation and treatment of fragility fractures achieved the best outcomes in terms of cost effectiveness. The re-fracture risk (hazard ratio [HR] 0.18–0.67 over 2–4 years), reduced mortality (HR 0.65 over 2 years), increased assessment of BMD (relative risk [RR] 2–3), increased treatment initiation (RR 1.5–4.25) and adherence to treatment (65%–88% at 1 year).

**Comments**

Because of the large scale of these review studies, and the lack of country-specific impact estimates for all patient outcomes, this metric focuses mostly on literature which estimates the impact of introducing FLS. The large variation between different types of FLSs and their evaluation complicates the assessment of the overall benefits of FLS and merits of a specific FLS model. Initiatives that promote standardised outcome frameworks for assessing FLS and increased collaboration between providers include the Capture the Fracture® and the UK FLS-Database Audit.

| **Outcome measure** | **Effect of FLS (absolute change)** | **95% CI** | **Duration of follow-up (months)** | **# studies** | **Source** |
| --- | --- | --- | --- | --- | --- |
| *BMD Testing* | +24% | (0.18 to 0.29) | 3-26 | 37 | [99] |
| *Treatment initiation* | +20% | (0.16 to 0.25) | 3-72 | 46 |  |
| *Adherence* | +22% | (0.13 to 0.31) | 3-48 | 9 |  |
| *Re-fracture* | -5% | (-0.08 to -0.03) | 6-72 | 11 |  |
| *Mortality* | -3% | (-0.05 to -0.01) | 6-72 | 15 |  |

**Table A 33** Meta-analysis results for patient outcomes

**Table A 34** Country-specific studies on the economic impact of FLS

| Country | Type | Estimate | Source |
| --- | --- | --- | --- |
| *Sweden* | ICER (cost-eff) | € 14,029 (per QALY gained) | [101] |
| *UK (Hip patients)* | ICER (cost-eff) | €22,700-€26,600 (per QALY gained) | [102] |
| *UK* | Cost Savings | €23,800/lifetime/1,000 patients | [95] |

**20:** **Capture the Fracture (Fracture Liaison Services)**

**Background**

Fracture Liaison Services (FLS) function as the first line of defence against repeat fragility fractures by identifying at-risk patients and referring them to specialists for assessment and preventative treatment. FLS have been shown to reduce the risk of fracture, mortality and increase adherence to treatment, which raises their quality of life and reduces societal costs[92, 94, 96-98].

However, there are currently no official international standards for, nor much collaboration between FLS providers in different countries. This gives rise to varying standards and practices on a national and sometimes local basis. One endeavour to encourage cooperation between FLS providers is *Capture the Fracture*, a global initiative to “facilitate the implementation of coordinated, multi-disciplinary models of care for secondary fracture prevention” [94].

**Methods**

Capture the Fracture (CtF) has created a set of internationally endorsed standards and guides for best practice and has assembled the largest network of individual FLS providers in the world. The program is organized by a steering committee of medical and academic professionals from a variety of backgrounds.

CtF provides resources, tools and educational programmes to bridge the gap between FLS providers and aids in the establishment of new FLS. They connect experienced FLS professionals with prospective providers to offer auditing, consulting and can then guide the assembly of a team to oversee FLS in the interested party’s area. This ensures that the new FLS will also match the guidelines CtF promotes.

**Results**

This growing network of FLS providers is mapped (Fig. A 33) on their website and provides a rating of the existing service providers in a given area. To be included in this network, the provider must undergo CtF’s audit in order to determine the quality of their services.

A gold, silver or bronze is awarded to the FLS provider according to the average rating of each of the services they provide. Green stars represent providers currently under review and blue stars are providers that are just getting started. Table A 35 shows the star ratings for registered FLS providers in the countries of interest. A value of 4, 3 and 2 was applied to gold, silver and bronze, respectively and a 1 to providers currently under review or just getting started. However, because the CtF does not include all FLS it is difficult to draw any conclusion regarding the quality of FLS provision on a national level.

To date, Spain has by far the most registered FLS and gold rated providers, and Sweden has the highest average overall FLS rating. One crucial factor to note is the relatively low numbers of registered FLS providers in Sweden and Germany. As discussed in other metrics, both these countries have higher than average incidence of fragility fractures, and have, therefore, a greater need for FLS providers.

**Comments**


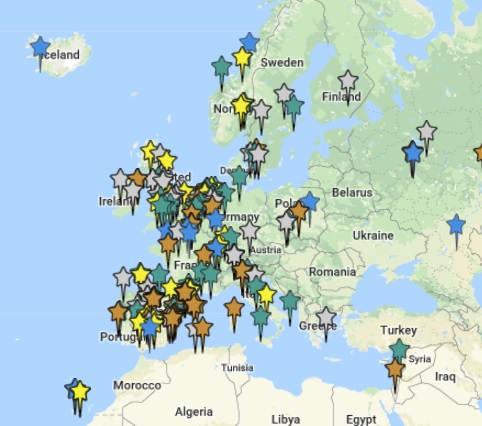
This a survey of the responsiveness of countries to the IOF Capture the Fracture initiative, and not a survey of the FLS themselves. Capture the Fracture not only helps to standardize Fracture Liaison Services but encourages cooperation between FLS providers. This in turn can allow for improved sharing of best practices and data that will promote a more efficient delivery of FLS.

**Fig. A 33** Capture the Fracture's map of best practice

**Table A 35 FLS** ratings by country.

| **Country** | **Total** | **Gold** | **Silver** | **Bronze** | **Green** | **Blue** | **Score** |
| --- | --- | --- | --- | --- | --- | --- | --- |
| *France* | 20 | 0 | 3 | 9 | 1 | 7 | 35 |
| *Germany* | 2 | 0 | 1 | 0 | 1 | 0 | 4 |
| *Italy* | 13 | 1 | 3 | 2 | 3 | 4 | 24 |
| *Spain* | 65 | 13 | 13 | 22 | 9 | 8 | 152 |
| *Sweden* | 5 | 0 | 4 | 1 | 0 | 0 | 14 |
| *UK* | 25 | 6 | 11 | 1 | 7 | 0 | 66 |
| *All* | 130 | 20 | 35 | 35 | 2 | 19 | 295 |

**References**

1. Kanis JA, Melton LJ, 3rd, Christiansen C, Johnston CC, Khaltaev N (1994) The diagnosis of osteoporosis. J Bone Miner Res 9:1137-1141

2. Looker AC, Wahner HW, Dunn WL, Calvo MS, Harris TB, Heyse SP, Johnston CC, Jr., Lindsay R (1998) Updated data on proximal femur bone mineral levels of US adults. Osteoporos Int 8:468-489

3. Kanis JA, Johnell O, Oden A, Jonsson B, De Laet C, Dawson A (2000) Risk of hip fracture according to the World Health Organization criteria for osteopenia and osteoporosis. Bone 27:585-590

4. Cawston H, Maravic M, Fardellone P, Gauthier A, Kanis JA, Compston J, Borgstrom F, Cooper C, McCloskey E (2012) Epidemiological burden of postmenopausal osteoporosis in France from 2010 to 2020: estimations from a disease model. Arch Osteoporos 7:237-246

5. Gauthier A, Kanis JA, Jiang Y, Dreinhofer K, Martin M, Compston J, Borgstrom F, Cooper C, McCloskey E (2012) Burden of postmenopausal osteoporosis in Germany: estimations from a disease model. Arch Osteoporos 7:209-218

6. Svedbom A, Hernlund E, Ivergard M, Compston J, Cooper C, Stenmark J, McCloskey EV, Jonsson B, Kanis JA (2013) Osteoporosis in the European Union: a compendium of country-specific reports. Arch Osteoporos 8:137

7. Oden A, McCloskey EV, Johansson H, Kanis JA (2013) Assessing the impact of osteoporosis on the burden of hip fractures. Calcif Tissue Int 92:42-49

8. Strom O, Borgstrom F, Kanis JA, Compston J, Cooper C, McCloskey EV, Jonsson B (2011) Osteoporosis: burden, health care provision and opportunities in the EU: a report prepared in collaboration with the International Osteoporosis Foundation (IOF) and the European Federation of Pharmaceutical Industry Associations (EFPIA). Arch Osteoporos 6:59-155

9. United Nations DESA / Population Division. (2017) World Population Prospects 2017. https://esa.un.org/unpd/wpp/Download/Standard/Population/.

10. Eurostat (2015) People in the EU: who are we and how do we live?

11. Icks A, Haastert B, Wildner M, Becker C, Meyer G (2008) Trend of hip fracture incidence in Germany 1995–2004: a population-based study. Osteoporosis international 19:1139-1145

12. Diez A, Puig J, Martinez MT, Diez JL, Aubia J, Vivancos J (1989) Epidemiology of fractures of the proximal femur associated with osteoporosis in Barcelona, Spain. Calcified tissue international 44:382-386

13. Elffors I, Allander E, Kanis J, Gullberg B, Johnell O, Dequeker J, Dilsen G, Gennari C, Vaz AL, Lyritis G (1994) The variable incidence of hip fracture in southern Europe: the MEDOS Study. Osteoporosis international 4:253-263

14. Kanis J, Johnell O, Oden A, Sernbo I, Redlund-Johnell I, Dawson A, De Laet C, Jonsson B (2000) Long-term risk of osteoporotic fracture in Malmö. Osteoporosis international 11:669-674

15. Piscitelli P, Chitano G, Johannson H, Brandi ML, Kanis JA, Black D (2013) Updated fracture incidence rates for the Italian version of FRAX®. Osteoporosis International 24:859-866

16. Izquierdo MS, Ochoa CS, Sánchez IB, Hidalgo MP, del Valle Lozano F, Martín TG (1997) Epidemiology of osteoporotic hip fractures in the province of Zamora (1993). Revista espanola de salud publica 71:357-367

17. Sosa M, Segarra M, Hernández D, González A, Limiñana J, Betancor P (1993) Epidemiology of proximal femoral fracture in Gran Canaria (Canary Islands). Age and ageing 22:285-288

18. Curtis EM, van der Velde R, Moon RJ, van den Bergh JP, Geusens P, de Vries F, van Staa TP, Cooper C, Harvey NC (2016) Epidemiology of fractures in the United Kingdom 1988–2012: variation with age, sex, geography, ethnicity and socioeconomic status. Bone 87:19-26

19. Institut national de la statistique et des études économiques (INSEE) (2015) Quotient de mortalité Q(x, x+1) pour 100 000 survivants à l'âge x (âge en années révolues) [Mortality Quotient Q (x, x + 1) per 100,000 survivors at age x (age in completed years)]. https://www.insee.fr/fr/statistiques/2851503?sommaire=2851587

20. Statistisches Bundesamt (DESTATIS) (2014) Life table (period life table): Germany, years, sex, completed age. Probability of death [q(x)]. https://www-genesis.destatis.de/genesis/online/data;jsessionid=250F248D9F4C7473F40C9067A9774254.tomcat_GO_2_1?operation=abruftabelleBearbeiten&levelindex=2&levelid=1517322385357&auswahloperation=abruftabelleAuspraegungAuswaehlen&auswahlverzeichnis=ordnungsstruktur&auswahlziel=werteabruf&selectionname=12621-0001&auswahltext=%23Z-01.01.2014%23SGES-

21. Instituto Nacional de Estadistica (INE) (2016) Tablas de mortalidad de la población de España por año, sexo, edad y funciones [Tables of mortality of the population of Spain by year, sex, age and functions]. http://www.ine.es/jaxi/Datos.htm?path=/t20/p319a/serie/p02/l0/&file=02001.px

22. Istituto Nazionale di Statistica (ISTAT) (2016) Probabilità di morte (per 1000) [Probability of death (per 1000)]. http://dati.istat.it/Index.aspx?lang=it&SubSessionId=46db532d-c21a-48a4-8745-b5611c62e6c8&themetreeid=21#

23. Statistiska Centralbyrån (SCB) (2016) Ettårig livslängdstabell, dödsrisker (promille) efter kön, ålder och år [One-year life-table, deaths (per 1000 ) by sex, age and year]. http://www.statistikdatabasen.scb.se/sq/44505

24. Office for National Statistics (ONS) (2014-2016) National life tables: Great Britain. Mortality rate. https://www.ons.gov.uk/peoplepopulationandcommunity/birthsdeathsandmarriages/lifeexpectancies/datasets/nationallifetablesgreatbritainreferencetables

25. World Health Organization (2013) Priority diseases and reasons for inclusion: Acute stroke. http://www.who.int/medicines/areas/priority_medicines/Ch6_6Stroke.pdf Accessed 04/12/2018

26. Lloyd-Jones DM, Leip EP, Larson MG, D'Agostino RB, Beiser A, Wilson PW, Wolf PA, Levy D (2006) Prediction of lifetime risk for cardiovascular disease by risk factor burden at 50 years of age. Circulation 113:791-798

27. (SCB) SS (2017) Population statistics. https://www.scb.se/en/finding-statistics/statistics-by-subject-area/population/population-composition/population-statistics/#_TablesintheStatisticalDatabase Accessed 2018-03-14 2018

28. Johansson H, Siggeirsdottir K, Harvey NC, Oden A, Gudnason V, McCloskey E, Sigurdsson G, Kanis JA (2017) Imminent risk of fracture after fracture. Osteoporos Int 28:775-780

29. Bonafede M, Shi N, Barron R, Li X, Crittenden DB, Chandler D (2016) Predicting imminent risk for fracture in patients aged 50 or older with osteoporosis using US claims data. Arch Osteoporos 11:26

30. Nordström P, Gustafson Y, Michaëlsson K, Nordström A (2015) Length of hospital stay after hip fracture and short term risk of death after discharge: a total cohort study in Sweden. Bmj 350:h696

31. Roux C, Briot K (2017) Imminent fracture risk. Osteoporos Int 28:1765-1769

32. Maravic M, Jouaneton B, Vainchtock A, Tochon V (2012) Economic burden of osteoporosis in women: data from the 2008 French hospital database (PMSI). Clin Exp Rheumatol 30:222-227

33. Bassgen K, Westphal T, Haar P, Kundt G, Mittlmeier T, Schober HC (2013) Population-based prospective study on the incidence of osteoporosis-associated fractures in a German population of 200,413 inhabitants. J Public Health (Oxf) 35:255-261

34. Carnevale V, Nieddu L, Romagnoli E, Bona E, Piemonte S, Scillitani A, Minisola S (2006) Osteoporosis intervention in ambulatory patients with previous hip fracture: a multicentric, nationwide Italian survey. Osteoporos Int 17:478-483

35. Caeiro JR, Bartra A, Mesa-Ramos M, Etxebarria I, Montejo J, Carpintero P, Sorio F, Gatell S, Farre A, Canals L (2017) Burden of First Osteoporotic Hip Fracture in Spain: A Prospective, 12-Month, Observational Study. Calcif Tissue Int 100:29-39

36. Leal J, Gray A, Prieto-Alhambra D, Arden NK, Cooper C, Javaid M, Judge A, group Rs (2016) Impact of hip fracture on hospital care costs: a population-based study. Osteoporosis International 27:549-558

37. Hernlund E, Svedbom A, Ivergård M, Compston J, Cooper C, Stenmark J, McCloskey EV, Jönsson B, Kanis JA (2013) Osteoporosis in the European Union: medical management, epidemiology and economic burden. Archives of osteoporosis 8:136

38. Stevenson M, Davis S (2006) Analyses of the cost-effectiveness of pooled alendronate and risedronate, compared with strontium ranelate, raloxifene, etidronate and teriparatide. The University of Sheffield, School of Health and Related Research

39. Stevenson M, Davis S, Kanis J (2006) The hospitalisation costs and out-patient costs of fragility fractures. Women's Health Medicine 3:149-151

40. Brecht J, Kruse H, Felsenberg D, Möhrke W, Oestreich A, Huppertz E (2003) Pharmacoeconomic analysis of osteoporosis treatment with risedronate. International journal of clinical pharmacology research 23:93-105

41. Visentin P, Ciravegna R, Fabris F (1997) Estimating the cost per avoided hip fracture by osteoporosis treatment in Italy. Maturitas 26:185-192

42. Borgström F, Zethraeus N, Johnell O, Lidgren L, Ponzer S, Svensson O, Abdon P, Ornstein E, Lunsjö K, Thorngren KG (2006) Costs and quality of life associated with osteoporosis-related fractures in Sweden. Osteoporosis international 17:637-650

43. Lange A, Zeidler J, Braun S (2014) One-year disease-related health care costs of incident vertebral fractures in osteoporotic patients. Osteoporosis International 25:2435-2443

44. Hart W, Rubio-Terres C, Burrell A, Aristegui I, Escobar-Jiménez F (2002) Análisis farmacoeconómico del tratamiento de la osteoporosis postmenopáusica con risedronato o alendronato. Revista Española de Enfermedades Metabólicas Óseas 11:97-104

45. Gutiérrez L, Roskell N, Castellsague J, Beard S, Rycroft C, Abeysinghe S, Shannon P, Gitlin M, Robbins S (2012) Clinical burden and incremental cost of fractures in postmenopausal women in the United Kingdom. Bone 51:324-331

46. Ström O, Borgström F, Sen S, Boonen S, Haentjens P, Johnell O, Kanis J (2007) Cost-effectiveness of alendronate in the treatment of postmenopausal women in 9 European countries-an economic evaluation based on the fracture intervention trial. Osteoporosis international 18:1047-1061

47. Jönsson B, Ström O, Eisman JA, Papaioannou A, Siris E, Tosteson A, Kanis JA (2011) Cost-effectiveness of denosumab for the treatment of postmenopausal osteoporosis. Osteoporosis international 22:967-982

48. Gutiérrez L, Roskell N, Castellsague J, Beard S, Rycroft C, Abeysinghe S, Shannon P, Robbins S, Gitlin M (2011) Study of the incremental cost and clinical burden of hip fractures in postmenopausal women in the United Kingdom. Journal of medical economics 14:99-107

49. Davis S, Martyn-St James M, Sanderson J, Stevens J, Goka E, Rawdin A, Sadler S, Wong R, Campbell F, Stevenson M, Strong M, Selby P, Gittoes N (2015) Bisphosphonates for preventing osteoporotic fragility fractures (including a partial update of NICE technology appraisal guidance 160 and 161). Technology Assessment Report: Final report to the National Institute for Health and Care Excellence.

50. Borgstrom F, Sobocki P, Strom O, Jonsson B (2007) The societal burden of osteoporosis in Sweden. Bone 40:1602-1609

51. Eurostat (2017) Healthcare expenditure statistics. http://ec.europa.eu/eurostat/statistics-explained/index.php/Healthcare_expenditure_statistics

52. Sassi F (2006) Calculating QALYs, comparing QALY and DALY calculations. Health Policy Plan 21:402-408

53. Svedbom A, Borgstrom F, Hernlund E, Strom O, Alekna V, Bianchi ML, Clark P, Curiel MD, Dimai HP, Jurisson M, Uuskula A, Lember M, Kallikorm R, Lesnyak O, McCloskey E, Ershova O, Sanders KM, Silverman S, Tamulaitiene M, Thomas T, Tosteson ANA, Jonsson B, Kanis JA (2017) Quality of life after hip, vertebral, and distal forearm fragility fractures measured using the EQ-5D-3L, EQ-VAS, and time-trade-off: results from the ICUROS. Qual Life Res 27(3):707-716

54. Szende A, anssen B, and Cabases J (2014) Self-reported population health: an international perspective based on EQ-5D. Dordrecht (NL): Springer

55. Johnell O, Kanis, J.A., Odén, A. et al.,, (2004) Mortality after osteoporotic fractures. Ostoporos Intl 15: 38-42

56. World Health Organization (2018) Metrics: Disability-Adjusted Life Year (DALY). http://www.who.int/healthinfo/global_burden_disease/metrics_daly/en/ Accessed 12/10/2017

57. Kanis JA, Oden A, Johnell O, Jonsson B, de Laet C, Dawson A (2001) The burden of osteoporotic fractures: a method for setting intervention thresholds. Osteoporos Int 12:417-427

58. Barrett JA, Baron JA, Beach ML (2003) Mortality and pulmonary embolism after fracture in the elderly. Osteoporos Int 14:889-894

59. Office for National Statistics (2018) Population Estimates for UK, England and Wales, Scotland and Northern Ireland. https://www.ons.gov.uk/peoplepopulationandcommunity/populationandmigration/populationestimates/datasets/populationestimatesforukenglandandwalesscotlandandnorthernireland

60. Statistiches Bundesamt (2016) Genesis: Population Tables. https://www-genesis.destatis.de/genesis/online/link/tabellen/12411*&language=en

61. Statistika Centralbyrån (2018) Folkmängden efter region, civilstånd, ålder och kön. År 1968 - 2016. http://www.statistikdatabasen.scb.se/pxweb/sv/ssd/START__BE__BE0101__BE0101A/BefolkningNy/?rxid=86abd797-7854-4564-9150-c9b06ae3ab07

62. Institut national d'études démographiques (2018) Population by age. https://www.ined.fr/en/everything_about_population/data/france/population-structure/population-age/ Accessed 01/02/2018

63. Istituto Nazionale di Statistica (2018) I.Stat: Data by Theme. http://dati.istat.it/?lang=en

64. European Statistical System (2018) Census Data by Country. https://ec.europa.eu/CensusHub2/query.do?step=selectHyperCube&qhc=false Accessed 01/02/2018

65. World Health Organization (2018) Disability weights based on patient-reported data from a multinational injury cohort. http://www.who.int/bulletin/volumes/94/11/BLT-16-172155-table-T3.html

66. Kanis JA, Oden A, Johnell O, De Laet C, Jonsson B, Oglesby AK (2003) The components of excess mortality after hip fracture. Bone 32:468-473

67. Kanis JA, Oden A, Johnell O, De Laet C, Jonsson B (2004) Excess mortality after hospitalisation for vertebral fracture. Osteoporos Int 15:108-112

68. Dept. of Information EaRW, , (2017) WHO Methods and Data Sources for Global Burden of Disease Estimates 2000-2015. http://www.who.int/healthinfo/global_burden_disease/GlobalDALYmethods_2000_2015.pdf Accessed 01/17/2017

69. Institute for Health Metrics and Evaluation (IHME) (2016) GBD Compare Data Visualization. https://vizhub.healthdata.org/gbd-compare/

70. Johnell O, Kanis JA (2006) An estimate of the worldwide prevalence and disability associated with osteoporotic fractures. Osteoporos Int 17:1726-1733

71. Borgstrom F, Lekander I, Ivergard M, Strom O, Svedbom A, Alekna V, Bianchi ML, Clark P, Curiel MD, Dimai HP, Jurisson M, Kallikorm R, Lesnyak O, McCloskey E, Nassonov E, Sanders KM, Silverman S, Tamulaitiene M, Thomas T, Tosteson AN, Jonsson B, Kanis JA (2013) The International Costs and Utilities Related to Osteoporotic Fractures Study (ICUROS)--quality of life during the first 4 months after fracture. Osteoporos Int 24:811-823

72. Svedbom A, Borgstom F, Hernlund E, Strom O, Alekna V, Bianchi ML, Clark P, Curiel MD, Dimai HP, Jurisson M, Kallikorm R, Lember M, Lesnyak O, McCloskey E, Sanders KM, Silverman S, Solodovnikov A, Tamulaitiene M, Thomas T, Toroptsova N, Uuskula A, Tosteson ANA, Jonsson B, Kanis JA (2018) Quality of life for up to 18 months after low-energy hip, vertebral, and distal forearm fractures-results from the ICUROS. Osteoporos Int 29:557-566

73. Kaffashian S, Raina P, Oremus M, Pickard L, Adachi J, Papadimitropoulos E, Papaioannou A (2011) The burden of osteoporotic fractures beyond acute care: the Canadian Multicentre Osteoporosis Study (CaMos). Age Ageing 40:602-607

74. Kanis JA, Harvey NC, Cooper C, Johansson H, Oden A, McCloskey EV (2016) A systematic review of intervention thresholds based on FRAX : A report prepared for the National Osteoporosis Guideline Group and the International Osteoporosis Foundation. Arch Osteoporos 11:25

75. l’Assurance Maladie (2016) Améliorer la qualité du système de santé et maîtriser les dépenses. Propositions de l’Assurance Maladie pour 2016, 2016 edn

76. Klop C, Gibson-Smith D, Elders PJ, Welsing PM, Leufkens HG, Harvey NC, Bijlsma JW, van Staa TP, de Vries F (2015) Anti-osteoporosis drug prescribing after hip fracture in the UK: 2000-2010. Osteoporos Int 26:1919-1928

77. Harvey NC, McCloskey EV, Mitchell PJ, Dawson-Hughes B, Pierroz DD, Reginster JY, Rizzoli R, Cooper C, Kanis JA (2017) Mind the (treatment) gap: a global perspective on current and future strategies for prevention of fragility fractures. Osteoporos Int 28:1507-1529

78. Kanis JA, Borgstrom F, Compston J, Dreinhofer K, Nolte E, Jonsson L, Lems WF, McCloskey EV, Rizzoli R, Stenmark J (2013) SCOPE: a scorecard for osteoporosis in Europe. Arch Osteoporos 8:144

79. University of Sheffield (2008) FRAX Fracture Risk Assessment Tool. https://www.sheffield.ac.uk/FRAX/

80. Deutschsprachigen Wissenschaftlichen Osteologischen (2014) DVO Leitlinie Osteoporose 2014 Kurzfassung und Langfassung http://www.dv-osteologie.org/dvo_leitlinien/osteoporose-leitlinie-2014 Accessed 03/06/2018

81. Rendl S, Lapa C, Blumel C, Bundschuh RA, Schneider P (2013) Decision making for osteoporotic treatment using FRAX or DVO risk algorithms in a clinical setting. J Musculoskelet Neuronal Interact 13:339-345

82. Bonaccorsi G, Fila E, Cervellati C, Romani A, Giganti M, Rossini M, Greco P, Massari L (2015) Assessment of Fracture Risk in A Population of Postmenopausal Italian Women: A Comparison of Two Different Tools. Calcif Tissue Int 97:50-57

83. National Clinical Guideline C (2012) National Institute for Health and Clinical Excellence: Guidance. Osteoporosis: Fragility Fracture Risk: Osteoporosis: Assessing the Risk of Fragility Fracture. Royal College of Physicians (UK) National Clinical Guideline Centre., London

84. l'Assurance Maladie (2018) Ostéoporose : diagnostic et évolution. https://www.ameli.fr/loiret/assure/sante/themes/osteoporose/diagnostic-evolution Accessed 03/06/2018

85. Tarantino U, Iolascon G, Cianferotti L, Masi L, Marcucci G, Giusti F, Marini F, Parri S, Feola M, Rao C, Piccirilli E, Zanetti EB, Cittadini N, Alvaro R, Moretti A, Calafiore D, Toro G, Gimigliano F, Resmini G, Brandi ML (2017) Clinical guidelines for the prevention and treatment of osteoporosis: summary statements and recommendations from the Italian Society for Orthopaedics and Traumatology. J Orthop Traumatol 18:3-36

86. Ministero della Salute (Italian Ministry of Health) (2011) 1.12. Prevenzione delle fratture da fragilità. http://www.rssp.salute.gov.it/rssp/paginaParagrafoRssp.jsp?sezione=risposte&capitolo=interventi&id=2745 Accessed 02/27/2018

87. Societa Italiana di Ortopedia e Traumatologia (2017) Le Linee Guida. http://www.siot.it/pagine/soci/linee-guida.html Accessed 03/13/2018

88. Ministerio de Sanidad SSeI, , (Spanish Ministry of Health) (2010) Guía de Práctica Clínica sobre Osteoporosis y Prevención de Fracturas por Fragilidad. http://www.guiasalud.es/GPC/GPC_476_Osteoporosis_AIAQS_compl.pdf Accessed 02/27/2018

89. Socialstyrelsen (The Swedish Welfare Agency) (2018) Nationella riktlinjer för rörelseorganens sjukdomar – stöd för styrning och ledning. http://www.socialstyrelsen.se/publikationer2012/2012-5-1 Accessed 02/27/2017

90. The National Institute for Health and Care Excellence (NICE) (2018) Osteoporosis: assessing the risk of fragility fracture. https://www.nice.org.uk/guidance/cg146/chapter/1-guidance Accessed 02/27/2018

91. Mitchell PJ (2011) Fracture Liaison Services: the UK experience. Osteoporos Int 22 Suppl 3:487-494

92. Eekman DA, van Helden SH, Huisman AM, Verhaar HJ, Bultink IE, Geusens PP, Lips P, Lems WF (2014) Optimizing fracture prevention: the fracture liaison service, an observational study. Osteoporos Int 25:701-709

93. Huntjens KM, van Geel TA, van den Bergh JP, van Helden S, Willems P, Winkens B, Eisman JA, Geusens PP, Brink PR (2014) Fracture liaison service: impact on subsequent nonvertebral fracture incidence and mortality. J Bone Joint Surg Am 96:e29

94. International Osteoporosis Foundation (2018) Capture the Fracture. http://www.capture-the-fracture.org/

95. McLellan AR, Wolowacz SE, Zimovetz EA, Beard SM, Lock S, McCrink L, Adekunle F, Roberts D (2011) Fracture liaison services for the evaluation and management of patients with osteoporotic fracture: a cost-effectiveness evaluation based on data collected over 8 years of service provision. Osteoporos Int 22:2083-2098

96. Nakayama A, Major G, Holliday E, Attia J, Bogduk N (2016) Evidence of effectiveness of a fracture liaison service to reduce the re-fracture rate. Osteoporos Int 27:873-879

97. Schray D, Neuerburg C, Stein J, Gosch M, Schieker M, Bocker W, Kammerlander C (2016) Value of a coordinated management of osteoporosis via Fracture Liaison Service for the treatment of orthogeriatric patients. Eur J Trauma Emerg Surg 42:559-564

98. Walters S, Khan T, Ong T, Sahota O (2017) Fracture liaison services: improving outcomes for patients with osteoporosis. Clin Interv Aging 12:117-127

99. Wu CH, Tu ST, Chang YF, Chan DC, Chien JT, Lin CH, Singh S, Dasari M, Chen JF, Tsai KS (2018) Fracture liaison services improve outcomes of patients with osteoporosis-related fractures: A systematic literature review and meta-analysis. Bone 111:92-100

100. Wu CH, Kao IJ, Hung WC, Lin SC, Liu HC, Hsieh MH, Bagga S, Achra M, Cheng TT, Yang RS (2018) Economic impact and cost-effectiveness of fracture liaison services: a systematic review of the literature. Osteoporos Int 29(6):1227-1242

101. Jonsson E, Borgström F, Ström O (2016) PHS49 - Cost Effectiveness Evaluation of Fracture Liaison Services for the Management of Osteoporosis in Sweden. Value in Health 19: A612

102. Leal J, Gray AM, Hawley S, Prieto-Alhambra D, Delmestri A, Arden NK, Cooper C, Javaid MK, Judge A (2017) Cost-Effectiveness of Orthogeriatric and Fracture Liaison Service Models of Care for Hip Fracture Patients: A Population-Based Study. J Bone Miner Res 32:203-211
